# Supplementary material for: Special Issue: Family Planning
Source: Glob Health Action. 2015 Nov 9;8:10.3402/gha.v8.30086. doi: 10.3402/gha.v8.30086 (PMC4642484; doi:10.3402/gha.v8.30086)
Supplement: Related texts

# Sub article: Meeting demand for family planning within a generation: prospects and implications at country level

**DOI:** 10.3402/gha.v8.29734

**Published:** 2015-11-09

**Authors:** Yoonjoung Choi, Madeleine Short Fabic, Sennen Hounton, Desmond Koroma

**Affiliations:** 1 United States Agency for International Development, Washington, DC, USA; 2 United Nations Population Fund, New York, NY, USA

**Keywords:** demand for family planning, modern contraception, Sustainable Development Goals

## Abstract

**Background:**

In order to track progress towards the target of universal access to sexual and reproductive health care services of the post-2015 Sustainable Development Goals (SDGs), a measure (demand for family planning satisfied with modern contraceptive methods) and a benchmark (at least 75% by 2030 in all countries) have been recommended.

**Objectives:**

The goal of this study was to assess the prospects of reaching the benchmark at the country level. Such information can facilitate strategic planning, including resource allocation at global and country levels.

**Design:**

We selected 63 countries based on their status as least developed according to the United Nations or as a priority country in global family planning initiatives. Using United Nations estimates and projections of family planning indicators between 1970 and 2030, we calculated percent demand for family planning satisfied with modern contraceptive methods for each year and country. We then calculated the annual percentage point changes between 2014 and 2030 required to meet the benchmark. The required rates of change were compared to current projections as well as estimates between 1970 and 2010.

**Results:**

To reach the benchmark on average across the 63 countries, demand satisfied with modern methods must increase by 2.2 percentage points per year between 2014 and 2030 – more than double current projections. Between 1970 and 2010, such rapid progress was observed in 24 study countries but typically spanning 5–10 years. At currently projected rates, only 9 of the 63 study countries will reach the benchmark. Meanwhile, the gap between projected and required changes is largest in the Central and West African regions, 0.9 and 3.0 percentage points per year, respectively. If the benchmark is achieved, 334 million women across the study countries will use a modern contraceptive method in 2030, compared to 226 million women in 2014.

**Conclusions:**

In order to achieve the component of the SDGs calling for universal access to sexual and reproductive health services, substantial effort is needed to accelerate rates of progress by a factor of 2 in most study countries and by a factor of 3 in Central and West African countries.

**Paper context**

Achieving universal access to sexual and reproductive health care services is a target of the post-2015 Sustainable Development Goals (SDGs). To monitor progress, a measure (demand for family planning satisfied with modern contraceptive methods) and a benchmark (at least 75% by 2030 in all countries) have been recommended. This paper assesses the prospects of reaching the benchmark at the national level in 63 selected countries. Study results show that substantial effort is needed to accelerate rates of progress, by a factor of 2 in most study countries, suggesting a need for strategic planning, including resource allocation, at the global and country levels.

Expanding access to family planning has been a key objective of health and development programmes for decades. The 2012 London Summit on Family Planning and other global and regional initiatives brought renewed emphasis on family planning's importance to health (1–3), economic wellbeing (4), empowerment, and environment (5). Accelerating family planning's progress is again a driving aim of the international community and many individual countries.

Aligning with global and local initiatives and evidence, the Sustainable Development Goals (SDGs) include the target of ‘universal access to sexual and reproductive healthcare services, including for family planning, information and education, and the integration of reproductive health into national strategies and programmes’ (6). To track the progress of this target, a measure (demand for family planning met with modern contraceptive methods) and a benchmark (at least 75% by 2030 in all countries) have been suggested (7) and have received positive feedback from the international family planning community and beyond. Recently, the United Nations Technical Support Team to the General Assembly Open Working Group on the SDGs recommended the indicator, and scores of national statistics organisations described it as ‘highly relevant’ to the SDGs (8).

The measure reflects the aim of family planning programmes – to support the rights of individuals and couples to choose whether and when to have a child by providing them with effective means to implement their decisions – and promotes voluntarism, informed choice, rights, and equity (7, 9). The proposed benchmark aligns with current levels of demand for family planning being satisfied in most developed countries and for various subpopulations in developing countries, as well as with historical experiences of formerly low-income countries (7). The benchmark is also ambitious: it will only be achieved if progress towards meeting demand for family planning is accelerated, especially in low-income countries.

It is critical to understand the progress that will be required on a country-by-country basis to best direct resources and guide strategic planning. Our study aims to understand prospects of meeting the benchmark by 2030 using data from 63 selected countries. Specific objectives were as follows: 1) to estimate the progress needed to meet the benchmark (i.e. at least 75% of demand for family planning satisfied with modern contraceptive methods) in each country and 2) to compare the required progress to current projections by United Nations as well as historic experience. Further, in order to illustrate various prospects across countries, we examine the data in detail for three countries: Burkina Faso, Ethiopia, and Nigeria.

## Methods

### Study countries

We selected 63 countries for the study to understand country-level prospects. These countries are either among the least developed countries, according to the United Nations classification (10) or priority countries for international family planning initiatives. Annex 1 in the Supplementary file presents a complete list of the study countries. Of these countries, we drew upon data from Burkina Faso, Ethiopia, and Nigeria for country-level illustration and further analysis to understand within-country variation. We opportunistically selected these three countries in order to align with other analyses included in this Special Issue. Additionally, these countries reflect diverse demographic and economic characteristics and varying levels of family planning programmes especially in recent decades, despite being in the same geographic region (Table 1). Finally, in order to understand the prospects of the 63 countries in an historic context, we analysed estimates data between 1970 and 2010 from all available 194 countries in the database, as described below.

**Table 1:** Selected current population and economic characteristics of Burkina Faso, Ethiopia, and Nigeria

	Burkina Faso	Ethiopia	Nigeria
Total fertility rate	5.9	4.1	5.6
Population (millions)	18	96	178
Rate of natural population increase (%)	3.1	2.1	2.5
Female population 15–49 years of age who have no education (%)^a^	74	51	38
Gross national income per capita, purchasing power parity (US$)	1,560	1,350	5,600

Source: World Population Data Sheet 2014 (Population Reference Bureau), www.prb.org/pdf14/2014-world-population-data-sheet_eng.pdf, unless noted.

a Burkina Faso Demographic and Health Survey (DHS) 2010, Ethiopia DHS 2011, Nigeria DHS 2013.

### Data

Data on country-specific estimates and projections of family planning indicators were from an estimates and projections database of the United Nations Population Division (11). The database includes model-based annual estimates and projections of selected family planning indicators from 1970 to 2030 for 194 countries (www.un.org/en/development/desa/population/publications/dataset/contraception/wcu2014.shtml). The indicators include contraceptive prevalence rate (CPR), modern contraceptive prevalence rate (MCPR), and unmet need for family planning among women between 15 and 49 years of age who are married or in a union. Estimates and projections are based on a Bayesian hierarchical model combined with country-specific time trends, which captures observed fluctuations around the main trends within countries. The modelling data set includes estimates of family planning indicators from a large number of population-based surveys, including country-specific national surveys – some of which were conducted as early as in the 1950s – as well as long-standing global survey programmes such as Demographic and Health Surveys, Multiple Indicator Cluster Surveys, and Reproductive Health Surveys. Detailed methods on the statistical modelling are provided elsewhere (12). For each indicator, the database presents median estimates with 80 and 95% uncertainty bounds. For this study, the median estimate was used for all analyses.

### Measurement and analysis

First, for each of the 194 countries and for the years between 1970 and 2030, we calculated the proposed SDG indicator – percent demand for family planning met with modern contraceptive methods (the ratio of MCPR to the sum of CPR and unmet need for family planning). For the 63 study countries, we then calculated the projected absolute percentage point changes per year between 2014 and 2030 for the indicator. We also calculated the absolute percentage point changes per year between 2014 and 2030 that would be needed to achieve the proposed benchmark – at least 75%, hereinafter referred to as a *benchmark scenario* – using a simple assumption of linear changes in each country. We believe that such a simple calculation is useful for the purpose of this study to provide prospects and facilitate discussion for strategic planning, even though the assumption of linear trends over 15 years was crude, especially for countries currently at a low level. The two annual changes (projected vs. required for the benchmark scenario) were compared by calculating both absolute and relative differences for each country. In addition, we calculated the absolute number of women who would use modern methods in 2030 according to current projections and the benchmark scenario. It should be noted that, under the benchmark scenario, we assume that the currently projected demand trajectory will remain even if MCPR increases rapidly. This assumption ignores a positive relationship between MCPR and demand (12) and may underestimate demand as MCPR accelerates under the benchmark scenario. Thus, our approach may underestimate the required progress as well as the amount of resources needed to respond to increasing demand under the benchmark scenario.

In order to understand the required progress under the benchmark scenario in the context of historic experience, we calculated the annual absolute percent point changes in the proposed SDG indicator over eight 5-year periods (1970–1975, 1975–1980, 1980–1985, 1985–1990, 1990–1995, 1995–2000, 2000–2005, and 2005–2010) using estimates from all 194 countries. We then compared the annual changes in the benchmark scenario for the 63 study countries with these observed, historic annual changes from 194 countries between 1970 and 2010.

Descriptive analyses were conducted using figures and summary statistics, which were unweighted averages across the study countries. To investigate regional patterns, we classified the countries into three groups: Central and West Africa (*n*=22), South and East Africa (*n*=19), and other (*n*=22) (Annex 1 in the Supplementary file). STATA 13.0 statistical software was used for all analyses (Stata Corporation, College Station, TX, USA).

## Results

On average across the 63 study countries in 2014, among women in union, 59% had demand for family planning. Of those women in union who had demand for family planning, on average 43% used modern contraceptive methods, with a large variation among countries ranging from 6% in South Sudan to 84% in Bhutan. Figure 1 presents the current levels of family planning demand satisfied with modern methods and modern contraceptive prevalence among the study countries (in red) and non-study countries (in black). In our three illustrative countries – Nigeria, Burkina Faso, and Ethiopia – 28, 40, and 54% of women who had demand for family planning used modern methods, respectively. Of note, five study countries (Bhutan, Honduras, Indonesia, South Africa, and Zimbabwe) already exceeded the 75% benchmark. Across 49 least developed countries (according to the United Nations classification), on average about 40% of women with demand for family planning used modern methods, compared to 67 and 70% in less and more developed countries. Again, there was great variation across countries, even among the 49 least developed countries, highlighted by the data from five countries (South Sudan, Somalia, Chad, Guinea, and Democratic Republic of Congo) where the level was 15% or below.

**Fig. 1:** Percentage of demand for family planning met with modern contraceptive methods and modern contraceptive prevalence rates in 194 countries in 2014. Large grey circles represent the unweighted average of countries by development classification by United Nations: least, less, and more developed countries (*n*=49, 103, and 42, respectively).

Table 2 presents annual changes between 2014 and 2030 according to the current projections and the benchmark scenario in the 63 study countries. Across our study countries, an average annual increase of 2.2 percentage points is needed to achieve the benchmark scenario, compared to the annual increase of 0.9 percentage points in current projections. The average difference between the two rates of annual change is 1.3 percentage points (additional absolute increase) or 2.5 times (relative difference).

**Table 2:** Percentage demand for family planning met with modern methods (2014 and 2030), annual rates of change (2014–2030), and the number of women using modern methods according to current projection and benchmark scenarios among 63 selected countries

	All study countries	West and Central Africa	East and South Africa	Other
Indicator	(*n*=63)	(*n*=22)	(*n*=19)	(*n*=22)
Mean family planning demand satisfied with modern methods (%)^a^				
2014	43.3	27.7	46.7	56.1
2030	56.4	42.4	63.0	64.7
Mean annual rate of change, 2014–2030 (percentage points)^a^ ^b^				
Current projection	0.9	0.9	1.1	0.6
Required by benchmark scenario	2.2	3.0	2.0	1.4
Mean difference in annual rate of changes between benchmark scenario and current projections^a^ ^b^				
Absolute difference (percentage points)	1.3	2.0	0.9	0.8
Relative difference (ratio)	2.5	3.2	1.7	2.3
Total number of women (thousands)^c^				
2014	533,808	71,199	65,419	397,190
2030	615,063	96,341	92,889	425,833
Total number of women using modern methods (thousands)^c^				
2014	225,841	7,989	21,564	196,289
2030, current projection	300,629	21,214	45,431	233,984
2030, benchmark scenario^d^	333,744	39,068	50,994	243,683

a Mean is unweighted average of national-level values across countries;

b excluding five countries where family planning demand satisfied with modern methods in 2014 was 75% or above;

c total number of women is aggregate sum across countries (*women* refers to those who are between 15 and 49 years of age and married or in a union);

d nine countries where projection is 75% or higher, the number of women according to the projected level was used.

Figure 2 illustrates differences in progress between the current projection (solid blue line) and benchmark scenario (dotted blue line) in Burkina Faso, Ethiopia, and Nigeria. In Ethiopia, the MCPR is projected to be 56% in 2030, which equates to 75% of family planning demand. Ethiopia shows little difference between the projected and required annual rates of change (Table 3). If its currently projected progress is realised, Ethiopia will achieve the family planning component of SDGs. In contrast, current projections for Burkina Faso and Nigeria indicate that only 52 and 42% of demand for family planning, respectively, will be satisfied with modern method use by 2030. Differences between projections and the benchmark scenario in these two countries are substantial (Table 3). To reach the benchmark, Burkina Faso must have an average yearly growth of 2.2 percentage points, and Nigeria must reach an average annual growth of 3.0 percentage points. Both rates of growth are much higher than those based on current projections – 0.8 and 0.9 percentage point increases per year for Burkina Faso and Nigeria, respectively.

**Fig. 2:** Demand for family planning and modern contraceptive prevalence rates in Burkina Faso, Ethiopia, and Nigeria: current estimates and projections versus progress needed to meet the benchmark of 75% by 2030. MCPR, modern contraceptive prevalence rate.

**Table 3:** Percentage of demand for family planning met with modern methods (2014 and 2030), annual rates of change (2014–2030), and the number of women using modern methods according to current projection and benchmark scenarios in Burkina Faso, Ethiopia, and Nigeria

Indicator	Burkina Faso	Ethiopia	Nigeria
Mean family planning demand satisfied with modern methods (%)			
2014	39.5	53.5	27.2
2030	51.7	74.4	41.9
Mean annual rate of change, 2014–2030 (percentage points)			
Current projection	0.8	1.3	0.9
Required by benchmark scenario	2.2	1.3	3.0
Difference in annual rate of change between the benchmark scenario and current projections			
Absolute difference (percentage points)	1.5	0.0	2.1
Relative difference (ratio)	2.9	1.0	3.2
Total number of women (thousands)^a^			
2014	3,078	13,935	26,645
2030	4,224	20,588	34,604
Total number of women using modern methods (thousands)^a^			
2014	551	4,529	2,691
2030, current projection	1,225	11,468	7,198
2030, benchmark scenario	1,777	11,565	12,873

a *Women* refers to those who are between 15 and 49 years of age and married or in a union.

As illustrated by these three country examples, vastly different prospects for achieving the benchmark are observed across all study countries. Differences between the current projection and benchmark scenario paths are largest in the Central and West African countries (*n*=22), where annual changes in the benchmark scenario were on average 3.2 times higher than those changes according to current projections (Table 2). In contrast, differences between the current projection and benchmark scenario are substantially smaller in the South and East African countries (*n*=19), where the average annual change required to meet the benchmark scenario is 1.7 times higher than current projections. In about one-quarter of our study countries, the current projection for 2030 is at or near the benchmark. Specifically, the current projection for 2030 is 75% or above in nine countries and is near the benchmark (i.e. 70–75%) in six countries (Annex 2 in Supplementary file).

Historic trends indicate that the progress required to achieve the benchmark will be challenging for the majority of the study countries. Figure 3 is a scatter plot of annual percentage point changes that were observed from 194 countries over each of the eight 5-year periods spanning the years 1970–2010. In the overwhelming majority of cases (94%), the annual change was below 2 percentage points. Further, the annual changes appeared to be higher when the initial level of demand satisfied was between 30 and 50%, as indicated by the quadratic fitted line. An annual increase of 2 percentage points or higher was observed at least once in 24 study countries and 37 non-study countries. Of the 24 study countries, 2 were in Central and West Africa (Burkina Faso, from 15% in 2000 to 26% in 2005, and Nigeria, from 13% in 1990 to 24% in 1995); 12, including Ethiopia, were in South and East Africa; and 10 were outside Africa (Table 4). Nevertheless, in most of these countries, the rapid increase was observed over only 5- or 10-year periods, suggesting that maintaining the momentum over 15 years will be challenging. When we examined average rates of change over rolling 15-year periods between 1970 and 2010, only 13 study countries experienced annual average changes by 2 percentage points or more: Bangladesh, Bhutan, Cambodia, Ethiopia, India, Indonesia, Madagascar, Malawi, Mozambique, Myanmar, Rwanda, South Africa, and Zimbabwe (results not shown).

**Fig. 3:** Scatter plot of annual rate of changes in demand for family planning satisfied with modern methods by initial level: eight 5-year periods between 1970 and 2010 from 194 countries. Solid curved line is a quadratic fitted line (*R* ^2^ 0.17). A total of 1,552 observations are specific to a 5-year period and a country. The annual percentage point changes range from −3.87 (Timor-Leste 1995–2000) to 7.81 (Rwanda 2005–2010). Only 1,520 observations between the 1st and 99th percentile of the changes are shown, ranging from −0.78 to 3.50.

**Table 4:** List of countries and periods during which the annual change in family planning demand satisfied with modern methods exceeded two percentage points

		Demand for family planning satisfied with modern methods (%)		Annual rate of change (% points)
		
Country	Period	Beginning	Ending	
Afghanistan	2000–2005	14.7	27.4	2.5
Bangladesh	1980–1985	23.6	35.1	2.3
Bangladesh	1985–1990	35.1	46.3	2.2
Bangladesh	1990–1995	46.3	58.1	2.4
Bhutan	1990–1995	30.8	41.3	2.1
Bhutan	1995–2000	41.3	53.0	2.3
Bhutan	2000–2005	53.0	69.0	3.2
Bhutan	2005–2010	69.0	81.8	2.5
Burkina Faso	2000–2005	15.1	26.2	2.2
Burundi	2005–2010	20.3	32.2	2.4
Cambodia	1995–2000	18.9	32.9	2.8
Djibouti	2000–2005	12.8	26.8	2.8
Ethiopia	2000–2005	13.7	27.7	2.8
Ethiopia	2005–2010	27.7	46.9	3.9
India	1970–1975	22.1	32.8	2.1
India	1975–1980	32.8	45.4	2.5
Indonesia	1970–1975	16.8	32.3	3.1
Indonesia	1975–1980	32.3	49.8	3.5
Indonesia	1980–1985	49.8	62.4	2.5
Kenya	1985–1990	20.0	32.2	2.4
Laos	1995–2000	35.8	47.3	2.3
Lesotho	1985–1990	20.1	30.7	2.1
Lesotho	2005–2010	55.0	65.8	2.2
Madagascar	2000–2005	25.5	37.9	2.5
Madagascar	2005–2010	37.9	49.6	2.3
Malawi	1995–2000	23.1	40.8	3.5
Mozambique	1995–2000	16.7	28.9	2.4
Myanmar	1990–1995	30.9	43.8	2.6
Myanmar	1995–2000	43.8	54.0	2.1
Nepal	1985–1990	22.9	33.1	2.0
Nigeria	1990–1995	13.3	23.7	2.1
Rwanda	2005–2010	18.4	57.4	7.8
South Africa	1970–1975	28.7	42.5	2.8
South Africa	1975–1980	42.5	54.5	2.4
Timor-Leste	2005–2010	29.6	40.7	2.2
Tanzania	1990–1995	16.8	28.4	2.3
Zimbabwe	1975–1980	18.8	31.2	2.5
Zimbabwe	1980–1985	31.2	45.8	2.9
Zimbabwe	1985–1990	45.8	57.4	2.3

An annual increase of three percentage points or more – the average level of progress needed among the Central and West African countries – was observed over a 5-year period in five study countries (Bhutan, Ethiopia, Indonesia, Malawi, and Rwanda) (Table 4) and 14 non-study countries. However, when we examined the rapid increase sustained over a 15-year period, only six countries in the world experienced annual average changes by 3 percentage points or more over 15 years or longer during the last four decades: Belgium (from 24% in 1970 to 82% in 1987), Botswana (from 3% in 1972 to 56% in 1992), Colombia (from 20% in 1970 to 65% in 1985), France (from 23% in 1970 to 77% in 1987), Indonesia (from 17% in 1970 to 69% in 1988), and Réunion (from 25% in 1970 to 76% in 1987).

Figure 3 also shows that some countries experienced negative changes (7% of total observation points). Whereas most of the negative changes were less than 1 percentage point per year, substantial reductions were often observed in the wake of serious turmoil, civil unrest, and war. These examples include Timor-Leste (from 53% in 1995 to 34% in 2000), Serbia (from 38% in 2000 to 28% in 2005), Mozambique (from 37% in 2005 to 30% in 2010), and Rwanda (from 15% in 1995 to 9% in 2000).

Lastly, we examined the absolute number of women who would use modern methods in 2030 under current projections and the benchmark scenario based on 1) population projections of women of reproductive age who are in a union; 2) demand for family planning according to current projections; and 3) demand for family planning satisfied with modern methods according to both current projections and the benchmark scenario. The examples of Burkina Faso, Ethiopia, and Nigeria (Table 3) show that the number of women who would use modern methods in 2030 based on current projections is 1.2 million, 11.5 million, and 7 million, respectively. As expected, the difference in the number of women using modern methods in 2030 between the current projection and the benchmark scenario is minimal in Ethiopia, but substantial in Burkina Faso and Nigeria: The absolute number of women who would be using modern methods was 45 and 79% higher in the benchmark scenario than the current projection, respectively. Throughout all 63 study countries, 300 million and 334 million women would be using modern methods in 2030, according to the current projection and benchmark scenario, respectively (Table 2). In 22 Central and West African countries, almost twice as many as women would be using modern methods under the benchmark scenario (39 million) compared to the current projection (21 million) (Table 2).

## Discussion

Our study examines the prospects of 63 selected countries towards achieving the family planning indicator and benchmark for the SDG target of universal access to sexual and reproductive health services. There is great variation across the study countries, but on average, a sustained increase of 2.2 percentage points per year is required over the next 15 years. Compared to current projections, the rates of change in the 63 study countries need to double in order to achieve the benchmark. In the Central and West Africa regions, progress needs to almost triple.

Examination of historic rates of progress shows that rapid annual increases by 2 percentage points or higher over 5 years have been observed in 24 of the 63 study countries. However, sustaining the rapid progress over an extended period is challenging, as shown by the fact that only 13 study countries achieved rapid progress by two percentage points or more per year over a 15-year period. Examples of annual increases by three percentage points or more over 15 years were rare. Further, only five study countries experienced an annual change of three percentage points or higher over a 5-year period and only one study country over a 10-year period. Nevertheless, examples from non-study countries, especially Botswana, where a rapid and sustained progress was made even with a very low initial level, suggest that the benchmark may be achievable.

Among our three illustrative countries, Ethiopia made remarkably rapid improvements between 2000 and 2010, thanks to political will and strategic programming (13, 14). Current projections suggest that Ethiopia is well on its way to meeting the benchmark by 2030. In Burkina Faso and Nigeria, two things should be noted. First, currently projected demand is relatively low, and the countries can achieve the benchmark of 75% with a fairly low level of MCPR. In countries that are in the beginning of fertility transition, implementing multisectoral approaches to generate demand for family planning is critical, in addition to efforts to satisfy current demand. Secondly, even with the relatively low demand that is projected currently, the two countries need to accelerate the rate of progress by about three times to meet the benchmark. Burkina Faso and Nigeria have had rapid increases historically, though only over one 5-year period each (Table 4). This historic experience suggests that, though ambitious, the benchmark can be achieved even in countries with currently low levels of demand satisfied with modern methods.

Nevertheless, there are substantial challenges to achieving the family planning benchmark in many countries. Simply to achieve current projections, which are based on assumptions of continuous and universal increases in modern contraceptive use across countries, will require continuous and intensive programme efforts. Historic data show that stagnation or even significant reversal can be observed in some countries under humanitarian crises. Thus, in order to accelerate currently projected rates of improvement, even greater amounts of resources, both financial and technical, and more efficient use of resources will be needed to stimulate demand for family planning and improve access. Indeed, a recent study showed financial resources need to almost double to fully address all current needs for sexual and reproductive health services, including modern contraception, in developing countries (15).

While our study focuses on national-level prospects, an important factor for achieving the ‘universal access’ component of the SDGs is addressing inequalities within each country. Further examination of data from Burkina Faso, Ethiopia, and Nigeria reveals within-country inequality (Fig. 4). Disaggregated data by household wealth quintile and residential area are presented for illustrative purposes across the three countries, although monitoring disaggregated data by other characteristics such as administrative unit – especially in the context of programming and/or decentralisation – is imperative. Some subpopulations such as women living in urban areas or highest wealth quintile households have made strong progress. In Ethiopia, especially, family planning demand satisfied with modern methods is already near the SDG benchmark among women in the highest wealth quintile households. In contrast, progress in disadvantaged populations is much slower. Further, in Nigeria, family planning demand satisfied with modern methods decreased among women in the lowest wealth quintile households during the last decade, although the national average increased steadily. At a more global scale, a recent study using data from 187 Demographic Health Surveys conducted in 53 countries showed that national average and within-country disparity are inversely related; as national average for demand satisfied with modern methods increases, within-country disparity decreases (16). The study also reported that within-country disparity by socio-economic background has decreased in the last two decades in most countries. Recognising the imperative of the SDGs to leave no one behind, efforts to satisfy demand for family planning must focus on bridging the inequality gaps across subpopulations.

**Fig. 4:** Trends in demand satisfied with modern methods by household wealth and residential area: Burkina Faso, Ethiopia, and Nigeria, by socio-economic and rural–urban status. Source: Demographic and Health Surveys through STATcompiler.com.

One limitation of our study is that we used an estimates and projections database that included only women who were currently married or in a union. Omission of unmarried, sexually active women in the database is the consequence of current inconsistent data availability among unmarried women at the global scale, not because of lack of importance of the population. Age at marriage has increased in many countries around the world, and there are a growing number of unmarried, sexually active women who have demand for family planning. Further, all women, regardless of their marital status – or any other characteristic such as young age – must have full and equal access to appropriate information and quality contraception services in order to be able to achieve their reproductive intentions to delay, space, or limit pregnancy. Thus, to align the indicator with the intentions of the goal and benchmark, the global database should include unmarried, sexually active women. That being said, the impact of these data on the estimate may not be substantial because this is a small population.

The data and findings presented provide baseline information to identify strategies needed at both global and country levels to achieve the family planning component of the SDGs. For example, our findings foremost show a great need to increase efforts and improve efficiency of investment in order to achieve the family planning component of the SDGs. Our identification of countries that have achieved rapid and sustained progress provides the geography of family planning policy and programmatic successes to explore and replicate as appropriate (13, 14, 17, 18). Historic examination also suggests progress may accelerate once populations reach a certain level, but until that point great efforts would be needed both to generate demand and to address existing demand. Finally, our identification of countries that have experienced declines in satisfied demand – though a small number – highlights the negative impacts conflicts and wars can have in relation to access to sexual and reproductive health services. This historic information is valuable to the international family planning community as evidence of the need to mitigate the detrimental impacts of existing and future tumult on family planning access.

Substantial variations in current levels and future prospects for demand satisfied among study countries raises questions for international and bilateral development organisations as well as countries. For example, how will both global and domestic financial and technical resources be mobilised and used over the next 15 years to achieve maximal outcomes and impact? How should development partners prioritise focal countries considering vastly different prospects even among the 63 study countries? What are approaches for monitoring at the global level, balancing achievement of the benchmark in each country versus reaching the largest absolute number of potential beneficiaries in highly populous countries?

## Conclusions

On average across the 63 study countries, family planning demand satisfied with modern contraceptive methods needs to increase by 2.2 percentage points per year for the next 15 years – more than double current projections. Yet historic experience during the last four decades shows that such rapid progress has been very infrequently achieved. More financial and technical resources and more efficient use of them, including a regional focus on Central and West Africa, are therefore critical to enabling countries to accelerate progress and achieve the ambitious benchmark scenario of at least 75% of family planning demand being satisfied with modern methods.

## Supplementary Material

Meeting demand for family planning within a generation: prospects and implications at country level Click here for additional data file.

## References

[1] Cleland J, Conde-Agudelo A, Peterson H, Ross J, Tsui A (2012). Contraception and health. Lancet.

[2] Ahmed S, Li Q, Liu L, Tsui AO (2012). Maternal deaths averted by contraceptive use: an analysis of 172 countries. Lancet.

[3] Rutstein SO (2005). Effects of preceding birth intervals on neonatal, infant and under-five years mortality and nutritional status in developing countries: evidence from the demographic and health surveys. Int J Gynecol Obstet.

[4] Canning D, Schultz TP (2012). The economic consequences of reproductive health and family planning. Lancet.

[5] UNFPA (2008). Family planning and the environment: stabilizing population would help sustain the planet.

[6] Open Working Group on Sustainable Development Goals (2014). Thirteenth session of the Open Working Group on Sustainable Development Goals. Outcome document – Introduction to the Proposal of the Open Working Group for Sustainable Development Goals.

[7] Fabic MS, Choi Y, Bongaarts J, Darroch JE, Ross JA, Stover J (2014). Meeting demand for family planning within a generation: the post-2015 agenda. Lancet.

[8] United Nations Statistical Commission (2015). Technical report by the Bureau of the United Nations Statistical Commission on the process of the development of an indicator framework for the goals and targets of the post-2015 development agenda.

[9] WHO (2014). Ensuring human rights in the provision of contraceptive information and services.

[10] United Nations, Department of Economic and Social Affairs, Population Division (2013). World population prospects: the 2012 revision, volume I: comprehensive tables (ST/ESA/SER.A/336).

[11] United Nations, Department of Economic and Social Affairs, Population Division (2014). Model-based estimates and projections of family planning indicators.

[12] Alkema L, Kantorova V, Menozzi C, Biddlecom A (2013). National, regional, and global rates and trends in contraceptive prevalence and unmet need for family planning between 1990 and 2015: a systematic and comprehensive analysis. Lancet.

[13] Olson DJ, Piller A (2013). Ethiopia: an emerging family planning success story. Stud Fam Plann.

[14] United States Agency for International Development, Ethiopia Federal Ministry of Health, Malawi Ministry of Health, Rwanda Ministry of Health (2012). Three successful Sub-Saharan Africa family planning programs: lessons for meeting the MDGs.

[15] Singh S, Darroch JE, Ashford LS (2014). Adding it up: the costs and benefits of investing in sexual and reproductive health 2014.

[16] Choi Y, Fabic MS (2015). Meeting demand for family planning: monitoring progress and equity. Report on the 2015 conference on global health post 2015 accelerating equity.

[17] Schuler SR, Hashemi SM, Jenkins HA (1995). Bangladesh's family planning success story: a gender perspective. Int Fam Plan Perspect.

[18] Habumuremyi PD, Zenawi M (2012). Making family planning a national development priority. Lancet.

# Sub article: Estimating family planning coverage from contraceptive prevalence using national household surveys

**DOI:** 10.3402/gha.v8.29735

**Published:** 2015-11-09

**Authors:** Aluisio J. D. Barros, Ties Boerma, Ahmad R. Hosseinpoor, María C. Restrepo-Méndez, Kerry L. M. Wong, Cesar G. Victora

**Affiliations:** 1 International Center for Equity in Health, Federal University of Pelotas, Pelotas, Brazil; 2 World Health Organization, Geneva, Switzerland

**Keywords:** family planning, contraception, coverage, prevalence, health indicators

## Abstract

**Background:**

Contraception is one of the most important health interventions currently available and yet, many women and couples still do not have reliable access to modern contraceptives. The best indicator for monitoring family planning is the proportion of women using contraception among those who need it. This indicator is frequently called *demand for family planning satisfied* and we argue that it should be called *family planning coverage* (FPC). This indicator is complex to calculate and requires a considerable number of questions to be included in a household survey.

**Objectives:**

We propose a model that can predict FPC from a much simpler indicator – contraceptive use prevalence – for situations where it cannot be derived directly.

**Design:**

Using 197 Multiple Indicator Cluster Surveys and Demographic and Health Surveys from 82 countries, we explored least-squares regression models that could be used to predict FPC. Non-linearity was expected in this situation and we used a fractional polynomial approach to find the best fitting model. We also explored the effect of calendar time and of wealth on the models explored.

**Results:**

Given the high correlation between the variables involved in FPC, we managed to derive a relatively simple model that depends only on contraceptive use prevalence but explains 95% of the variability of the outcome, with high precision for the estimated regression line. We also show that the relationship between the two variables has not changed with time. A concordance analysis showed agreement between observed and fitted results within a range of ±9 percentage points.

**Conclusions:**

We show that it is possible to obtain fairly good estimates of FPC using only contraceptive prevalence as a predictor, a strategy that is useful in situations where it is not possible to estimate FPC directly.

**Paper context**

Measuring progress towards universal health coverage involves producing estimates of health intervention coverage. For that purpose, coverage indicators that are accurate and easy to collect are needed. The existing indicator to estimate contraceptive use among women who need it (family planning coverage, or FPC) is complicated because it depends on several items of information that are difficult to ascertain. We propose, for situations where this complex indicator is not available, a way to estimate it from a much simpler indicator, namely the proportion of women using contraception in the population. This estimate is precise enough to be used as a substitute for FPC where it is not possible to estimate the latter directly.

Family planning is one of the most important health interventions currently available, and the global reduction in family size has contributed substantially to decreases in maternal and under-five mortality (1, 2). One review, including a simulation of the impact of different levels of investment in health, concluded that expanding access to modern contraceptives was the most cost-effective intervention, with great potential for reducing child and maternal deaths (3). It has also been argued that important social and economic benefits of increased access to family planning, such as improved women's earnings and more investments in schools, are expected. Economic growth was also linked to reduction in fertility rates because of increased participation of women in the labour force and reduction of young dependents in the households (4). Yet many women and couples still do not have reliable access to modern contraceptives. Recently, a global partnership, Family Planning 2020 (5), was established to support the rights of women and girls to decide, freely and for themselves, whether, when, and how many children they want to have.

The monitoring of progress towards the Millennium Development Goal target for achieving universal access to reproductive health (also known as *Target 5B*) involves two indicators related to contraception: contraceptive use prevalence [contraceptive prevalence rate (CPR)] and unmet need for family planning. The Commission on Information and Accountability for Women's and Children's Health proposed an alternative indicator, percent of demand for family planning satisfied (FPS) (6).

In addition to demographic and health impact, progress monitoring should be based on intervention coverage indicators, which are defined as the proportion of people receiving the services they need. This is justified because coverage indicators respond more promptly to programme changes than impact indicators, thus representing an invaluable tool for programme evaluation and consequent correction (7). CPR is a simple current status indicator to compute and understand: the percentage of women (15–49 years of age, currently married or in union) who are using any type of contraception. It is, however, not a true coverage indicator as not all women are in need of contraceptives, making it difficult to set meaningful targets. Unmet need indicators are more complex and have been frequently misunderstood (8), both in terms of their interpretation and in terms of their mathematical interrelationships. There is especially a tendency to suppose that percentage met need is 100 minus unmet need, which is not true given that the denominator of both indicators include women who do not need contraception. Recently, a target of 75% for an FPC indicator – percent of demand satisfied – has been proposed in the context of measuring progress towards the goals of family planning for 2020 and beyond (9).

In this paper, we first argue that family planning demand satisfied is the most useful indicator of coverage and propose that it should be referred to as *FPC*. The data requirements for the FPC indicator are quite considerable and it is less commonly measured in surveys than CPR, hampering comparisons between populations, equity analyses, and trend assessments. Therefore, our analysis aims to show how data on CPR can be used to estimate the FPC indicator in different settings.

## Methods

### Indicators

The calculation of either unmet need or FPC requires dividing women of fertile age into groups of need for contraception. The first group includes women who are infecund or menopausal, women who are pregnant or in postpartum amenorrhoea who wanted the pregnancy, and women who want another child in the next 2 years (Fig. 1, groups in blue). In this group, we have women with no need for contraception. The second group includes women who do need contraception, because they want no more children, want to wait, or did not want the current or recent pregnancy. These women can be further divided into those using contraceptives (Fig. 1, green), and those not using contraceptives (Fig. 1, orange). Unmet need for family planning is defined as the proportion of women with unmet need in relation to all women [orange/(blue+green+orange)]. This is probably the most common indicator used in the literature, but it is important to note that the denominator includes women who do not need contraception and thus, like CPR, it is not a coverage indicator.

**Fig. 1:** Groups of women in terms of need for contraception and its use.

FPC is a ‘positive’ (in the sense that higher is better) alternative to unmet need and the only true coverage indicator, being defined as the proportion of women that need contraception who are actually using contraceptives [green/(green+orange)]. As with all coverage indicators, it is easily interpretable in the sense that it can actually vary from 0 to 100%. From here on, we will refer to this indicator as *FPC*.

Given that the denominators of these two indicators are different, it is easy to see that FPC is not one minus unmet need, as is sometimes commonly believed.

All current definitions refer to women who are married or in union. It is, however, possible to simply extend the computations to include all sexually active women, including those who are not married or in a union. Another alternative to the standard survey definition is to estimate CPR and FPC only for modern contraceptives.

Details for the definitions of women that are infecund or in postpartum amenorrhoea are presented in a revision for unmet need definition published by the Demographic and Health Survey (DHS) programme (10). Despite the simplification proposed in the definitions of *unmet need* and *FPC*, the classification of women into the groups needed to estimate the indicators is complex and requires the inclusion of a large number of questions in a survey, some of them very subjective. Following Fig. 1, FPC (the proportion of women using contraception among those who need it) and CPR (the proportion of women using contraception) can be written as

$$\text{FPC}=\frac{\text{women using contraception}(\text{green})}{\text{women in need of contraception}(\text{green}+\text{orange})}$$

$$\text{CPR}=\frac{\text{women using contraception}(\text{green})}{\text{all women}(\text{blue}+\text{green}+\text{orange})}$$

Dividing both the numerator and denominator of FPC by the total number of women aged 15–49 years (blue+green+orange) we get

FPC=CPR/%women in need of contraception(green+orange)

and it therefore follows that

FPC=CPR/(1-% women not in need of contraception (blue))

Therefore, FPC depends on CPR and the proportion of women not in need of contraception.

Many surveys do not include the full array of questions required to obtain a direct estimate of unmet need or FPC. An estimate of coverage may however be needed, given the importance of FPC when not only CPR changes but also the desire for children, the need to assess trends over time using different surveys, or the use of FPC in combined health intervention coverage indicators such as the composite coverage indicator (11). The strong correlation between FPC and CPR has already been recognised in the literature (12) and used to predict FPC in surveys where only CPR was available.

In this work, we take this approach further exploring the relationship between FPC and its defining variables (CPR, proportion of women currently wanting children, proportion of women pregnant or in postpartum amenorrhea, and proportion of infecund or menopausal women) and proposing a predictive model for FPC.

### Data and analytical methods

We used DHS and Multiple Indicator Cluster Surveys (MICS) undertaken since the 1990s to explore the relationship between FPC and CPR and determine a predictive model that may be used as a simpler method to estimate FPC where it is not possible to estimate it directly. It may be useful in small surveys where there is interest in FPC but the number of questions involved is too large or for some DHS and MICS surveys where not all relevant questions were included. It may be also relevant when we want to estimate the composite coverage index, an indicator that involves an estimate for FPC along with other seven coverage indicators (13).

From a set of 238 DHS and MICS surveys, FPC was available for 173 DHS and for 24 MICS, totalling 197 surveys used for the modelling exercise, from 82 countries. Survey years ranged from 1993 to 2012. A full list of countries and respective surveys is presented in the Supplementary file.

All reproductive estimates used in this analysis refer to women aged 15–49 years who were married or in union at the time of the survey. CPR was defined as the prevalence of contraceptive use, either modern or traditional.

A revised definition of FPC was presented in 2012 (10) by a working group whose main objective was to make the indicator simpler and dependent upon fewer survey questions. In our analysis, however, we used the pre-2012 definition, because this was the one readily available from most surveys. In numerical terms, the difference between the two definitions was very small for the 21 surveys where both were available. The average difference between old and new definitions was 1.3 percentage points (95% limits of agreement –1.1 to 3.7 percentage points). A similar result was found for unmet need, with an average difference of 1.7 percentage points (10).

We explored the Pearson correlations between FPC, CPR, and the proportion of women wanting/having more children or who were infecund or menopausal. We also calculated the semi-partial correlations between these variables to estimate the percentage of CPR variability explained by both predictors after controlling for the others. Modelling was done using linear regression on a logit transform of FPC to avoid predicted values outside the [0–1] interval. We explored a range of models, using a fractional polynomial approach (14), in order to find the best performing one. In such models, we corrected the standard errors by taking into account repeated surveys for a particular country as a cluster. We also explored the relationship between FPC and CPR using estimates stratified by wealth quintiles. Similar analyses were performed and standard errors corrected by clusters of surveys.

The predicted values for FPC were back-transformed to its original scale in order to make results easier to understand.

## Results

Table 1 shows the distribution of our study variables: FPC, CPR, and the proportion of women wanting/having more children or who were infecund or menopausal. Both FPC and CPR varied widely – FPC from 12 to 94% and CPR from 3 to 79%. The percentage of women wanting/having more children varied from 4 to 53%, and the percentage of infecund or menopausal women varied from 5 to 37%.

**Table 1:** Mean, minimum, maximum, median, and 10th and 90th percentiles of the percentages of family planning coverage (FPC), contraceptive prevalence (CPR), women who want more children, and women who are infecund or menopausal

Variable	Mean	Minimum	10th percentile	Median	90th percentile	Maximum
FPC	61.7	11.8	30.0	62.8	89.3	94.3
CPR	40.6	2.8	12.6	40.0	70.5	79.0
% wanting/having more children	17.7	3.8	5.0	15.6	34.2	52.5
% infecund or menopausal	13.0	4.5	7.2	12.6	18.4	37.0

Source: DHS and MICS: 197 surveys from 1993 to 2012.

The correlations between FPC and its potential predictors (CPR, percentage of women wanting/having more children, and proportion of infecund or menopausal women) were 0.97, −0.76, and −0.61, respectively. We also observed strong negative correlation between CPR and the percentages of women wanting/having more children and those infecund or menopausal (−0.84 and −0.69, respectively).

The semi-partial squared correlations for percentages of women wanting/having more children and those infecund or menopausal adjusting for each other and for CPR were 0.8 and 0.6 percentage points, respectively, indicating that the addition of both variables in a predictive model where CPR was already included would add less than 1.5 percent points in its R^2^. This is explained by the strong correlation that exists between these predictors and CPR. Therefore,it is clear that we can model FPC using only CPR as a predictor, since the addition of the other predictors would improve any model only marginally.

Using the fractional polynomial strategy to find the best way to fit CPR in a model to predict the logit of FPC resulted in a model where CPR appears twice with powers one and two. This model can be written as follows:

$$\text{logit}(\text{FPC})=0\text{.61}+\text{0.68 log}(\text{CPR})+\text{3.57 CP}{\text{R}}^{\text{2}}$$

Compared to the simpler linear model, this alternative is better both in terms of reducing the deviance (*p=*0.028) and visually (Fig. 2). The results suggest that the association between the logit of FPC and CPR is not linear, especially when CPR is low. The proposed model explains 94.7% of the FPC variation (Table 2).

**Fig. 2:** Scatter plots plus linear and fractional polynomial regressions at national level and by wealth quintiles, showing the relationship between logit family planning coverage (FPC) and contraceptive prevalence rate (CPR). Source: DHS and MICS: 197 surveys from 1993 to 2012.

**Table 2:** Fractional polynomial model for predicting family planning coverage (FPC) from contraceptive prevalence rate (CPR)

Variable	Coefficient	*p*	95% CI			
National level						
Intercept	0.61	<0.001	0.38	0.84	*N=*197	*R* ^2^=94.7%
Log(CPR)	0.68	<0.001	0.55	0.80		
CPR^2^	3.57	<0.001	3.15	3.99		
Wealth quintiles						
Intercept	0.66	<0.001	0.51	0.80	*N=*959	*R* ^2^=95.5%
Log(CPR)	0.75	<0.001	0.67	0.82		
CPR^2^	3.58	<0.001	3.27	3.89		

Source: DHS and MICS, 197 surveys from 1993 to 2012.

Our next step was to assess whether this relationship changed over time. For that, we added the year of the survey to the previous model and found no effect (*p*=0.348). We also tested the interaction between year and CPR and again found no effect (*p*=0.328).

Finally, we explored whether wealth was an effect modifier of the association between FPC and CPR. For that, we used estimates of FPC and CPR stratified by wealth quintiles, from the same surveys described above. Figure 2 also shows the linear and fractional polynomial models for the data stratified by wealth quintiles, prior to adjustment by wealth. The results obtained are very similar to those at the national level (Table 2), and now with more data points at the low end of the CPR it is clear that the inflection of the curve fits the data well.

We found that there is an independent effect of wealth quintiles (*p*<0.001), but not effect modification (*p*=0.375). The results show that, for the same level of CPR, FPC increases slightly with wealth. The maximum difference between the poorest and the richest quintiles was observed when CPR was around 30%. At this point, FPC for the richest was only three percentage points higher than for the poorest (see Fig. A1 in the Supplementary file).

Given that we found no important effect of either time or wealth, our predictive model of FPC using CPR as the sole predictor is warranted. The analysis of the residuals suggested that the fit is good (details in the Supplementary file). We show in Fig. 3 the prediction curve with 95% confidence interval for the predicted average. A concordance analysis between predicted and observed values of FPC showed that the 95% limits of agreements were ±9.3 percentage points (see Fig. A4 in the Supplementary file).

**Fig. 3:** Predictive model for family planning coverage (FPC) based on contraceptive prevalence rate.

We also present, in Table 3, predicted values for FPC at selected levels of CPR. The predictions in the original FPC scale were obtained by back-transforming the results in the logit scale.

**Table 3:** Predicted values of family planning coverage (FPC) for a series of contraceptive prevalence (CPR) levels

CPR coverage (%)	Estimated FPC (%)	CPR coverage (%)	Estimated FPC (%)	CPR coverage (%)	Estimated FPC (%)
1^a^	8^a^	35	58	70	89
5	20	40	64	75	92
10	29	45	69	80	94
15	36	50	74	85^a^	96^a^
20	42	55	78	90^a^	97^a^
25	47	60	82	95^a^	98^a^
30	53	65	86	99^a^	98^a^

a Predictions outside the data range for CPR (2–79%).

## Discussion

FPC can be computed from surveys and is the most suitable indicator for the monitoring of progress of family planning programmes. However, the estimation of FPC involves a large number of questions in a survey and it is rather complex to calculate. The 2012 revised definition of *unmet need* tried to simplify its calculation and make it less dependent of unreliable questions (10). Still, around 15 questions are needed to establish all the defining items of this indicator, including whether 1) the woman is pregnant; 2) the woman is fecund; 3) she wants another baby within 2 years; 4) she is using contraception. A full list of the questions used in a DHS survey is presented in the DHS report ‘Revising unmet need for family planning’ (Appendix A in Ref. 10). CPR is a much simpler indicator and involves just asking whether the woman is using a contraceptive and, if yes, which one. However, it has several limitations. Most importantly, it is not a coverage indicator, as it will not (and should not) reach 100%, and there is no clear indication as to what is the desired prevalence for a given country.

Our analysis shows that it is possible to obtain a reliable and precise estimate of FPC from CPR alone. Using a large number of surveys covering a wide period (1993–2012) and different statistical models, we found a very strong association between FPC and CPR, yielding a predictive model that could explain 95% of the FPC variability. We also used estimates by wealth quintile to check whether the observed association at the country level would be different for wealth groups. The approach used to fit the models, fractional polynomials, was selected in order to allow for non-linearity in the association, while offering a large family of curves to choose from (14).

At a first look, being able to predict FPC from CPR alone may be a surprise given all the complexity involved in the definitions of FPC and unmet need, which include asserting women's current status in relation to fecundity, pregnancy, desire for more children, and so on. The answer lies in the empirical evidence that the defining variables of FPC, as we have shown, are highly correlated. Therefore, it is possible to make precise estimates of FPC without the need to take into account the other variables used in the calculation of FPC.

The increased sample size and variability of CPR offered by the analysis by wealth quintiles strengthened the results found at the country level. Specifically, it was possible to assess in more detail the shape of the association when CPR was very low, below 10%, which was more common for the poorest groups. It was also possible to show that there was no effect modification in the association between FPC and CPR by wealth group. The implication is that there is no need for adjustment for wealth or for group-specific predictions.

As expected, the proportion of women who want a child within 2 years is associated with FPC at the country level, but it makes very little difference to the final predictive model and can be ignored for all practical purposes.

The FPC indicator, as well as the conventional CPR, is limited to women who are married or in union. This is a drawback, as the use of family planning methods is at least as important for those not living in a union and exposed to the risk of pregnancy as for those in union (9). Many surveys report the proportion of sexually active unmarried women using contraception as well. Technically, a good additional FPC indicator would include all sexually active women of reproductive ages (i.e. exposed to the risk of pregnancy). This shift is beyond the scope of this paper, but the methods used here can be applied in the same way to find the best way to predict FPC among all sexually active women from CPR among all sexually active women at reproductive ages.

It has been proposed to focus the coverage indicator on modern contraceptives only (9). Here, we used both modern and traditional methods, but the method could easily be adapted to estimate coverage with modern contraceptives from the modern CPR.

Household surveys are the predominant source of data on contraceptive prevalence. Health facility data on current users and estimates of the population at risk (married women or sexually active women) could also provide an estimate of the CPR, which could then be converted into a coverage indicator. Currently, little use is made of such approaches because of data quality issues with facility data on family planning, such as incomplete reporting and double counting (see, e.g. www.cpc.unc.edu/measure/prh/rh_indicators/specific/fp/cpr).

As many countries embark on universal health coverage, monitoring progress becomes increasingly important. The WHO/World Bank Universal Health Coverage monitoring framework proposes a focus on a set of indicators of intervention coverage and financial protection (15). FPC is considered a good indicator for all countries and could be used as a tracer indicator (7). The statistical model proposed in this paper thus contributes to comparable monitoring of universal health coverage by improving the availability of data on FPC by socio-economic and other characteristics.

## Conclusions

FPC, also known as *demand for FPS*, is an important indicator for monitoring and policy making. It is also proposed as an indicator for monitoring one of the health targets of the sustainable development goals. In low- and middle-income countries, the FPC indicator is usually estimated from household surveys. However, it requires asking many additional questions, including some regarding exposure to the risk of pregnancy, fecundity, and desire for children. Many surveys only provide data on contraceptive prevalence and do not have adequate information to directly estimate FPC. Thus, we developed a predictive model that is at the same time simple and precise, making it possible to produce a very credible estimate of FPC using only contraceptive prevalence, which is an indicator obtained much more easily from surveys. Given that direct measurement of FPC takes into account several aspects such as whether women are fecund, want more children soon, and so on, along with their use of contraceptives, it may be surprising that we successfully tested such a simple model. The explanation is that all of these variables are highly correlated with each other, making such a model possible. FPC can reliably be predicted from contraceptive prevalence data using a simple mathematical model, which permits analysis of FPC trends and differentials using different types of surveys and allows the use of FPC in, for instance, analysis of progress towards universal health coverage.

## Supplementary Material

Estimating family planning coverage from contraceptive prevalence using national household surveys Click here for additional data file.

## References

[1] Ahmed S, Li Q, Liu L, Tsui AO (2012). Maternal deaths averted by contraceptive use: an analysis of 172 countries. Lancet.

[2] Cleland J, Conde-Agudelo A, Peterson H, Ross J, Tsui A (2012). Contraception and health. Lancet.

[3] Stenberg K, Axelson H, Sheehan P, Anderson I, Gülmezoglu AM, Temmerman M (2014). Advancing social and economic development by investing in women's and children's health: a new Global Investment Framework. Lancet.

[4] Canning D, Schultz TP (2012). The economic consequences of reproductive health and family planning. Lancet.

[5] Family Planning 2020 [Internet].

[6] Countdown to 2015 and the Health Metrics Network (2011). Monitoring maternal, newborn and child health: understanding key progress indicators.

[7] Boerma T, AbouZahr C, Evans D, Evans T (2014). Monitoring intervention coverage in the context of universal health coverage. PLoS Med.

[8] Bradley SEK, Casterline JB (2014). Understanding unmet need: history, theory, and measurement. Stud Fam Plann.

[9] Fabic MS, Choi Y, Bongaarts J, Darroch JE, Ross JA, Stover J (2014). Meeting demand for family planning within a generation: the post-2015 agenda. Lancet.

[10] Bradley SEK, Croft TN, Fishel JD, Westoff CF (2012). Revising unmet need for family planning. DHS Analytical Studies No. 25.

[11] Barros AJD, Victora CG (2013). Measuring coverage in MNCH: determining and interpreting inequalities in coverage of maternal, newborn, and child health interventions. PLoS Med.

[12] Boerma JT, Bryce J, Kinfu Y, Axelson H, Victora CG (2008). Mind the gap: equity and trends in coverage of maternal, newborn, and child health services in 54 Countdown countries. Lancet.

[13] Barros AJ, Ronsmans C, Axelson H, Loaiza E, Bertoldi AD, Franca GV (2012). Equity in maternal, newborn, and child health interventions in Countdown to 2015: a retrospective review of survey data from 54 countries. Lancet.

[14] Royston P, Sauerbrei W (2008). Multivariable model-building: a pragmatic approach to regression analysis based on fractional polynomials for modelling continuous variables [Internet].

[15] WHO (World Health Organization), WBG (World Bank Group) (2014). Monitoring progress towards universal health coverage at country and global levels: framework, measures and targets. Report No.: WHO/HIS/HIA/14.1.

# Sub article: Trends and patterns of modern contraceptive use and relationships with high-risk births and child mortality in Burkina Faso

**DOI:** 10.3402/gha.v8.29736

**Published:** 2015-11-09

**Authors:** Abdoulaye Maïga, Sennen Hounton, Agbessi Amouzou, Akanni Akinyemi, Solomon Shiferaw, Banza Baya, Dalomi Bahan, Aluisio J. D. Barros, Neff Walker, Howard Friedman

**Affiliations:** 1 Centre for Demographic Research, Université Catholique de Louvain, Louvain-la-Neuve, Belgium; 2 Institut Supérieur des Sciences de la Population, Ouagadougou University, Ouagadougou, Burkina Faso; 3 United Nations Population Fund, New York, NY, USA; 4 United Nations Children's Fund, New York, NY, USA; 5 Demographic and Social Statistics Department, Obafemi Awolowo University, Ife, Nigeria; 6 School of Public Health, Addis Ababa University, Addis Ababa, Ethiopia; 7 Institut National de la Statistique et de la Démographie, Ouagadougou, Burkina Faso; 8 United Nations Population Fund, Ouagadougou, Burkina Faso; 9 International Center for Equity in Health, Federal University of Pelotas, Pelotas, Brazil; 10 Institute for International Programs, Bloomberg School of Public Health, Johns Hopkins University, Baltimore, MD, USA

**Keywords:** modern contraceptive use, high-risk births, fertility, under-five child mortality, Burkina Faso

## Abstract

**Background:**

In sub-Saharan Africa, few studies have stressed the importance of spatial heterogeneity analysis in modern contraceptive use and the relationships with high-risk births.

**Objective:**

This paper aims to analyse the association between modern contraceptive use, distribution of birth risk, and under-five child mortality at both national and regional levels in Burkina Faso.

**Design:**

The last three Demographic and Health Surveys – conducted in Burkina Faso in 1998, 2003, and 2010 – enabled descriptions of differentials, trends, and associations between modern contraceptive use, total fertility rates (TFR), and factors associated with high-risk births and under-five child mortality. Multivariate models, adjusted by covariates of cultural and socio-economic background and contact with health system, were used to investigate the relationship between birth risk factors and modern contraceptive prevalence rates (mCPR).

**Results:**

Overall, Burkina Faso's modern contraception level remains low (15.4% in 2010), despite significant increases during the last decade. However, there are substantial variations in mCPR by region, and health facility contact was positively associated with mCPR increase. Women's fertility history and cultural and socio-economic background were also significant factors in predicting use of modern contraception. Low modern contraceptive use is associated with higher birth risks and increased child mortality. This association is stronger in the Sahel, Est, and Sud-Ouest regions. Even though all factors in high-risk births were associated with under-five mortality, it should be stressed that short birth spacing ranked as the highest risk in relation to mortality of children.

**Conclusions:**

Programmes that target sub-national differentials and leverage women's health system contacts to inform women about family planning opportunities may be effective in improving coverage, quality, and equity of modern contraceptive use. Improving the demand satisfied for modern contraception may result in a reduction in the percentage of women experiencing high-risk births and may also reduce child mortality.

**Paper context**

Modern contraceptive use is one of the most studied issues in reproductive health, but few things are known about differentials at the sub-national level and their association with risk-high birth and child mortality. Despite a low modern contraceptive use rate in Burkina Faso, regional disparities can reflect an issue of equity in terms of family planning (FP) coverage. However, women's contact with the health system may represent a critical opportunity for promoting and increasing contraceptive use. Furthermore, improving the modern contraceptive use can result in greater birth spacing and may contribute to a reduction in the child mortality rate.

Despite some notable advances in the past few decades in improving access to and involvement in FP, the percentage of demand for modern contraception satisfied remains low in most sub-Saharan countries, along with high rates of maternal, neonatal, and child mortality. The average total fertility rate (TFR) in Burkina Faso declined slightly from 6.8 children per woman (1998) to 6.0 (2010), but remains one of the highest in the world. Although the maternal mortality ratio has nearly halved since 1990, from 770 maternal deaths per 100,000 live births (1990) to 400 (2013), the ratio is still high (1).

Even though child mortality rates have dropped significantly in recent decades, the country is still suffering the burden of high childhood mortality. The under-five mortality rate in Burkina Faso was 129 per 1,000 births between 2005 and 2010 (2), while the most common causes of mortality in 2013 included malaria, neonatal causes, pneumonia, and diarrhoea (3). Mortality rates have been linked to mothers’ fertility characteristics, such as birth spacing, parity, or mothers’ age at childbirth (4, 5).

The sub-Saharan African region is characterised by a comparatively strong demand for larger families (6), and it has experienced less fertility decline than other regions of the world (7, 8). One of the key proximate determinants of fertility (9) is low contraceptive use, especially the use of modern contraceptive methods (6). Overall, about 15% of women in a union used modern contraceptive methods and 23.8% had an unmet need for FP in 2010 in Burkina Faso (2). This low contraception use is characteristic of many countries in sub-Saharan Africa, where there are estimated to be approximately 14 million unplanned pregnancies annually (10). However, there is substantial variation in contraceptive use both between and within countries. For instance, contraceptive prevalence was, on average, 15% in western Africa, while the rate exceeded 33% in some eastern African countries (11). At the sub-national level, contraceptive prevalence rates are generally lower in rural areas as compared to urban areas, generally resulting in higher fertility rates being observed in rural areas (12).

In addition, low contraceptive use may result in increased mortality risks for both mothers and children. Indeed, an increased contraceptive use would have reduced by 40 percentage points the risk of maternal death within the last 20 years and improved child survival by reducing the risks of prematurity or low weight at birth as a result of lengthening the interval between births in developing countries (13). The association between contraceptive use and mortality is indirectly observed through fertility factors such as early births (mother's age at birth <18 years), late births (mother's age at birth >34 years), short birth spacing (<24 months between births), and higher birth order or parity (>3). Births in which any (or a combination) of these indicators are present are referred to as *high-risk births* (14, 15), that is, births that represent a potential increased mortality risk to both mothers and children.

The low contraceptive use and subsequent difficulties in fertility regulation are explained in the literature by various barriers. Contraceptive method–related reasons have been identified and may relate to geographic and financial impediments to contraceptive access or FP services, including a limited choice of methods or experiencing side effects (16–18). There are medical barriers, such as the quality of services, providers’ biases, or medical and legal restrictions regarding access to certain methods (17–19). In some contexts, there are network barriers through social myths and misconceptions around contraception and partner and family approval of contraceptive decisions (20–22). Similarly, women's status in traditional society, place of residence, religious influence, social taboos, and cultural beliefs about high fertility represent potential sociocultural factors affecting modern contraceptive use (18, 22, 23). At the individual level, there are barriers related to women's background and around the quality of information about FP, the fear of adverse effects or the shame associated with affordability of particular methods, contact with health facilities, and women's sociodemographic characteristics (age, education, employment, marital status, parity, etc.) (16, 17, 21, 22).

Sociodemographics are the most studied of all factors explaining modern contraceptive use or non-use, as required variables are available in most data sources such as the Demographic and Health Surveys (DHS). Other barriers are often analysed through studies using mainly quantitative data from other specific cross-sectional surveys (20), and from qualitative data collection (23, 21).

Even though many investigations of modern contraceptive issues have been undertaken, few studies have shown an interest in exploring the relationship of birth risk and spatial heterogeneity. In this regard, the objective of this paper is to investigate the association between modern contraceptive use, distribution of birth risk, and under-five child mortality both nationally and regionally in Burkina Faso. Specifically, we describe differences in modern contraceptive use, TFR, and high-risk births over the time period of 1998–2010. The paper also investigates the association between modern contraceptive use, categories of high-risk births, and under-five child mortality. Lastly, the determinants or factors likely to be predictive of modern contraceptive use are explored at the national and sub-national levels.

## Methods

### Data and variables

The analyses were performed using data from the last three DHS, conducted in Burkina Faso in 1998, 2003, and 2010, which were representative at both the national and regional levels, available as secondary data. There were five administrative regions in the data set from the survey carried out in 1998. Following a national administrative re-districting in July of 2001, the 2003 and 2010 surveys each contained information for 13 regions. For consistency, we conducted comparison over time by administrative regions using only the last two surveys (2003 and 2010). However, comparisons over time were performed using all three surveys by stratifying by place of residence (urban vs. rural). In this respect, it is worth stressing that Burkina Faso is a predominantly rural country, with only 23% of the population living in urban areas. Except for the Centre (15%) and Hauts-Bassins (62%) regions, which contain the two largest cities (Ouagadougou and Bobo-Dioulasso), the percentage of rural population in the other regions ranges between 81 and 93%; the Sahel and Est regions are the most rural (24). In Burkina Faso, about 60% of citizens are Muslims, in rural areas as well as urban areas. Muslims are more prevalent in the Nord (80%) and Sahel (96%) regions while the biggest proportions of Christians are in the Centre-Ouest (38%) and Centre (42%) regions (24). There are about 60 ethnic groups in Burkina Faso, but almost half of the population belongs to the Mossi group, who primarily live in the Plateau Central, Centre, Centre-Nord, Nord, and Centre-Ouest regions. The Peulh/Fulfulde (mainly in the Sahel and Nord regions), Bobo (in the Hauts-Bassins region), and Gourmantche (in the Est region) are the other important ethnic groups (2).

The primary outcome of interest was the use of any modern contraceptive method at the time of the survey (current use). According to the definition commonly used in the DHS, modern contraceptive methods include the following: female sterilisation, male sterilisation, use of the contraceptive pill, intrauterine contraceptive devices, injectables, implants, female condoms, male condoms, diaphragms, contraceptive foam, contraceptive jelly, lactational amenorrhoea method, or other country-specific modern methods (25). The population base for assessing the coverage of these methods was women in a union (married or living with a partner at the time of the survey), aged 15–49 years old.

Concerning children, many studies have stressed the impact of birth spacing and, indirectly, of modern contraceptive use on perinatal outcomes, neonatal mortality, and deaths among children in the first year of life (26, 27). However, both infancy (<1 year) and early childhood periods (1–4 years) have been the major focus of other studies concerning birth interval and child survival relationships (4, 28). According to an analysis by Cleland et al. (13), the risk of death would fall by 10 and 21 percentage points in infancy and early childhood, respectively, if all childbirths were spaced by at least 2 years. In view of this evidence, we used the entire under-five group for analysis in this paper.

To examine the distribution of high-risk births, we explored the subset of women who had had at least one child within the 5 years preceding the survey. Births were assigned according to their risk factors (mother's age at the time of each childbirth, birth order, and birth spacing in relation to the previous birth) into the 11 categories commonly used (2, 14). These different categories are summarised in Table 1. Births can also be grouped into broader categories (i.e. single high risk or multiple high risk); combining single high risk and multiple high risk gives the category ‘any high risk’. For analyses where we considered the last childbirth for each woman in the preceding 5 years, birth order naturally corresponded to parity of births as well.

**Table 1:** Categories of risks related to fertility behaviour

Category	Definition	Abbreviation
No risk		
Not in any risk category	Second and third birth order born to mother between age 18 and 34 years and birth interval >23 months	No risk
Unavoidable risk		
First birth at age 18–34 years	First child born to mother between ages 18 and 34 years	1st birth+MA=18–34 y
Single high risk		
Mother's age <18 years	Mother's age at birth <18 years, birth order <4, and birth interval >23 months	MA<18 y+BO<4+BI>23 m
Mother's age >34 years	Mother's age at birth >34 years, birth order <4, and birth interval >23 months	MA>34 y+BO<4+BI>23 m
Birth interval <24 months	Birth interval <24 months, mother's age at birth 18–34 years, and birth order <4	BI<24 m+MA=18–34 y+BO<4
Birth order >3	Birth order >3, mother's age at birth 18–34 years, and birth interval >23 months	BO>3+MA=18–34 y+BI>23 m
Multiple high risk		
Mother's age <18 years and birth interval <24 months	Mother's age at birth <18 years and birth interval <24 months or mother's age at birth <18 years and birth order >3	MA<18 y+(BI<24 m∣BO>3)
Mother's age >34 years and birth interval <24 months	Mother's age at birth >34 years, birth interval <24 months and birth order <4	MA>34 y+BI<24 m+BO<4
Mother's age >34 years and birth order >3	Mother's age at birth >34 years, birth order >3, and birth interval >23 months	MA>34 y+BO>3+BI>23 m
Birth interval <24 months and birth order >3	Birth interval <24 months, birth order >3, and mother's age at birth 18–34 years	BI<24 m+BO>3+MA=18–34 y
Mother's age at birth >34 years, birth interval <24 months, and birth order >3	Mother's age at birth >34 years, birth interval <24 months, and birth order >3	MA>34 y+BI<24 m+BO>3

MA, mother's age at childbirth (y, years); BI, birth interval (m, months); BO, birth order.

In addition, covariates at the individual, household, and ecological levels were considered as potential confounders in regression analyses (see Supplementary file). These variables included households’ wealth quintile, mothers’ education level, mothers’ ethnicity, mothers’ religion, and mothers’ age at the time of the survey. Ecological covariates were place of residence (urban vs. rural) and the 13 administrative regions. We also used, as a covariate, the time since last childbirth, corresponding to the age of the youngest child at the time of the survey.

To look for a possible influence of gender preference on contraceptive use, we had covariates related to the number of surviving male and female children born to each woman in the 5 years preceding the survey. Contact with the health system regarding contraceptive use was reflected by indicators such as 1) whether a woman undertook a postnatal health facility visit within 2 months after the last birth and 2) whether a woman had received information about FP during a health facility visit within the last 12 months.

### Analysis

We analysed data using Stata 13.1 software (29). According to the survey design, all the analyses were run on weighted data where the Taylor linearisation series (TLS) (30, 31) was used for variance estimation. Descriptive statistics were used to describe patterns and trends of contraceptive use, distribution of high-risk births and under-five child mortality, and the interrelationships between these variables. To explore the relationship between contraceptive use, distribution of high-risk births, and under-five mortality at the regional level, we performed a time-series analysis where we first calculated women's modern contraceptive prevalence rates (mCPR) by region in 2003. Using 2003 as the reference year, fertility was observed for the next 5 years (until 2008). The regional distribution of high-risk births and the distribution of under-five mortality rates were computed for this same time period (see Figs. 4–6). A variance-weighted regression of TFRs was used to estimate the average annual rate of change (AAR) in fertility.

These analyses were carried out using the three surveys and considered both the national and sub-national levels. From the latest DHS (2010), we used multiple variable logistic regression models, adjusting for other covariates, to explore how high-risk birth recently experienced by women may have influenced modern contraceptive use afterwards. The high-risk birth factors were related to the characteristics of the latest childbirth for each woman between 2005 and 2010. Three regression models were developed, corresponding to rural areas, urban areas, and the entire population, for which adjusted odds ratios (aOR), 95% confidence intervals (95% CI), and *p*-values were reported.

## Results

### Descriptive analyses

#### Patterns and trends of modern contraceptive use

In Burkina Faso, the mCPR by women in a union with at least one child tripled from 1998 to 2010 (5.0–15.4%), yet still remained low (see Fig. 1). Women in rural areas had lower mCPR than those in urban areas across all time points. The mCPR in rural areas rose from 2.7% in 1998 to 11.3% in 2010. The largest absolute increase occurred in urban areas between 1998 and 2003, where there was an increase of nearly 10 percentage points. Indeed, 3 out of 10 urban women used a modern method in 2003, compared with 2 out of 10 in 1998. Nevertheless, we did not observe a significant change in modern contraceptive use in urban areas between 2003 and 2010, although the latest survey indicated that urban mCPR remained higher compared with the prevalence in rural areas.

**Fig. 1:** Modern contraceptive use by women in a union by year and by location of residence in Burkina Faso.

At the regional level (looking at administrative regions), we observed great disparities in modern contraception use between regions (see Table 2). In the latest survey (2010), mCPR was less than 10% in 4 out of 13 regions: Sahel (7.5%), Centre-Est (9.3%), Centre-Nord, and Centre-Ouest (9.8%). Generally speaking, the regions with the lowest mCPRs were more likely to be rural, poorer, have higher illiteracy rates, and belong to the Muslim or animist faiths. The highest mCPRs were observed in the Centre (31.3%) and Hauts-Bassins (28.1%) regions, which host Burkina Faso's political and economic capitals. From 2003 to 2010, contraception prevalence changed significantly in only four regions: Hauts-Bassins, Centre-Sud, Plateau Central, and Est. In Hauts-Bassins, mCPR increased by 16.0 percentage points during that time, while Centre-Sud, Plateau Central, and Est recorded gains of 11.5, 9.8, and 8.9 percentage points, respectively. Nationally, modern contraceptive use increased by 6.1 percentage points.

**Table 2:** Distribution of women in a union with at least one child by regional modern contraceptive prevalence rates and change over time

	mCPR 2003		mCPR 2010		mCPR change 2003–2010		Unweighted number	
				
Region	%	95% CI	%	95% CI	%	Sig.	2003	2010
Boucle du Mouhoun	11.0	(7.5–16.0)	12.1	(9.0–16.1)	1.1	ns	697	1,429
Cascades	13.8	(9.9–19.0)	18.8	(15.3–22.9)	5.0	ns	293	495
Centre	27.1	(21.6–33.4)	31.3	(27.5–35.4)	4.2	ns	791	1,337
Centre-Est	5.5	(3.4–8.9)	9.3	(7.0–12.2)	3.8	ns	747	964
Centre-Nord	7.3	(5.1–10.3)	9.8	(7.7–12.5)	2.5	ns	814	965
Centre-Ouest	6.5	(4.7–9.0)	9.8	(6.9–13.9)	3.3	ns	631	987
Centre-Sud	5.6	(4.2–7.5)	17.1	(13.8–20.9)	11.5	**	517	587
Est	2.5	(1.3–4.9)	11.4	(9.3–14.1)	8.9	**	724	1,199
Hauts-Bassins	12.1	(8.3–17.2)	28.1	(24.3–32.3)	16.0	**	1,111	1,409
Nord	8.9	(6.0–13.1)	10.8	(8.2–14.0)	1.9	ns	924	935
Plateau Central	5.1	(3.2–8.0)	14.9	(11.8–18.6)	9.8	**	453	610
Sahel	3.5	(1.6–7.2)	7.5	(5.1–10.8)	4.0	ns	656	1,014
Sud-Ouest	7.5	(4.0–13.7)	10.6	(8.0–13.9)	3.1	ns	431	537
Total	9.3	(8.0–10.7)	15.4	(14.2–16.7)	6.1	**	8,788	12,467

** Change significant at *p*<0.05; ns, change not significant at *p*<0.05; mCPR, modern contraceptive prevalence rates; CI, confidence interval; ns, not significant; sig., significance.

#### TFR and distribution of births by high-risk factors

Burkina Faso, with a TFR of 6.0 children per woman in 2010 (see Table 3), is among the countries with the highest fertility rates in the world. From 1998 to 2010, the AAR showed a significant but very small decrease in TFRs, barely −0.06 children per woman a year. The annual rate of fertility decline was greater over the 1998–2003 period (−0.11 children per woman a year), compared to the 2003–2010 period, where the AAR was not significant. Contrary to the change over time, there was substantial variation in the TFR according to place of residence and administrative region. In the 2010 survey, the TFR in rural areas (6.8 children per woman) was nearly double that of urban areas (3.9 children per woman). On the basis of administrative region (see Fig. 3), the TFR ranged from a low of 3.7 children per woman (Centre) to a high of 7.5 children per woman in the Sahel and Est regions. As expected, there was a negative relationship between contraception use and TFRs, where regions with the highest mCPRs had the lowest TFRs and vice versa.

**Table 3:** Trends of total fertility rates and standard errors of total fertility rates by location of residence in Burkina Faso

	Rural areas		Urban areas		Nationwide	
			
Year	TFR	SE-TFR	TFR	SE-TFR	TFR	SE-TFR
1998	7.31	(0.10)	4.05	(0.16)	6.80	(0.11)
2003	6.86	(0.10)	3.74	(0.20)	6.24	(0.13)
2010	6.85	(0.07)	3.92	(0.13)	6.09	(0.10)

TFR, total fertility rates; SE-TFR, standard errors of TFR.

The distribution of births by high-risk categories showed that 38% of births occurring between 2005 and 2010 were characterised by a single high-risk fertility behaviour, while 20.3% of births had multiple high-risk factors. In other words, nearly 6 out of 10 births (58.3%) had at least one high-risk factor (see Table 4). High parity birth (birth order >3) was the most substantial high risk factor in the single risk category, consistent with the observation that Burkina Faso has a high TFR. Short birth spacing (<24 months) was an important factor too, but it appeared more commonly as part of multiple high-risk factors. Within the multiple high-risk category, the most common combination was late birth (mother's age >34) combined with short birth spacing (13.8% of births), followed by the combination of short birth spacing with high parity birth (4.8%). The distribution of factors for high-risk birth exhibited no significant changes in the rate of single high-risk factors over time (2010 vs. 1998). Within multiple high-risk categories, only the category combining late birth with high parity birth recorded a slight significant decrease in rate (−2.0 percentage points) during this period.

**Table 4:** Distribution of births by risk category, changes over time, and relationships to under-five child mortality

	Births in the 5 years preceding each survey (%)			Under-five mortality rates in the 5 years preceding each survey (per 1,000)		Unweighted number of births	
			
Birth risk categories	2010	Change	1998–2010	2010	Change 1998–2010	1998	2010
Not in any high risk category	28.2	4.7	**	101.7	−94.7	1,410	4,177
Unavoidable risk category							
First birth between ages 18 and 34 years	13.4	1.2	ns	106.0	−143.3	730	1,985
Single high risk category							
Mother's age <18 years	4.6	−0.9	ns	161.7	−123.5	330	677
Mother's age >34 years	0.5	−0.2	ns	91.8	91.8	15	79
Birth interval <24 months	3.8	−0.9	ns	190.6	−43.1	280	569
Birth order >3	29.0	0.1	ns	123.6	−57.5	1,737	4,296
*Subtotal*	*38.0*	−*1.3*	ns	*134.9*	−*68.7*	2,363	5,620
Multiple high risk category							
Age <18 years and birth interval <24 months	[0.2]	[−0.5]	**	[291.6]	[75.3]	41	31
Age >34 years and birth interval <24 months	#	#	#	#	#	2	5
Age >34 years and birth order >3	13.8	−1.7	ns	112.1	−85.1	932	2,045
Birth interval <24 months and birth order >3	4.8	−2.0	**	212.4	−78.6	410	714
Age >34 years and birth interval <24 months and birth order >3	1.4	−0.6	ns	270.1	−5.4	119	212
*Subtotal*	*20.3*	−*4.7*	**	*155.1*	−*79.9*	1,504	3,006
In any high-risk category	58.3	−6.1	**	142.0	−73.9	3,867	8,626
Total	100	–		126.2	−89.4	6,008	14,789

** Change significant at *p*<0.05; ns, change not significant at *p*<0.05; [*x*], estimates based on unweighted cases between 25 and 50; #, estimates based on fewer than 25 unweighted cases.

Figure 2 presents the distribution of births classified as high risk, by place of residence and the change over time. For each year, we noted that the proportion of high-risk births was larger in rural areas than urban areas. Nearly 48.7% of births in 1998 in urban areas were in at least one high-risk category, while this proportion dropped to 40.7% in 2010. Conversely, 66% of rural births in 1998 were high risk compared with 61.7% in 2010. The national trends are more reflective of the rural trends, given that 84.5 and 77.3% of the population was rural in 1996 and 2006, respectively (24). The regional distribution of birth risk factors was examined (see Fig. 3). As expected, regions with a low TFR had a higher percentage of births with no risk compared with regions having a higher TFR. The percentage of high-risk births was lowest in the Centre and Hauts-Bassins regions and highest in the Sahel and Est regions. Consistent with expectations, the regions with the highest mCPRs had the lowest percentages of high-risk births.

**Fig. 2:** Distribution of high-risk births by location of residence over time in Burkina Faso.

**Fig. 3:** Distribution of births by risk factors and by region (total fertility rate: TFR) in 2010 in Burkina Faso.

#### Relationships between modern contraceptive use, high-risk births, and under-five child mortality

In general, there was a strong association between factors for high-risk birth and under-five mortality. Births belonging to any high-risk category had an under-five mortality rate of 142.0 deaths per 1,000 live births compared to 106.0 for births in the ‘unavoidable risk’ category and 101.7 for births classified as ‘not in any high-risk category’ (see Table 4). Among the high-risk categories, the mortality rate was 155.1 for multiple high-risk births compared with 134.9 for single high-risk births. The births that combined the risk factors of late birth, short birth spacing, and high birth parity had an under-five mortality rate of 270.1 per 1,000 live births compared with 212.1 for births that had short birth interval and high birth parity. Short birth spacing had the highest under-five mortality rate (190.0 per 1,000 live births) of any single high-risk factor. This factor also contributed to mortality in four out of the five multiple high-risk categories.

Figures 4–6 show a strong negative relationship of modern contraceptive use with the distribution of high-risk births and under-five mortality, where the regions characterised by low contraceptive use had the highest level of high-risk births and deaths of children, and vice versa. The Sahel region, with an mCPR of 3.5% in 2003, had 72.8% of births between 2003 and 2008 identified as high-risk and an under-five mortality rate of 205.0 per 1,000 live births. The mCPR, high-risk birth rates, and child mortality rates showed similar patterns in the Est (2.5%, 70.4%, and 157.8 per 1,000, respectively) and Sud-Ouest (7.5%, 65.0%, and 217.6 per 1,000, respectively) regions. The situation was reversed in the more urban Centre and Hauts-Bassins regions, where the comparatively higher mCPRs were associated with lower percentages of high-risk births and lower under-five mortality rates.

**Fig. 4:** Modern contraceptive prevalence rates by region in 2003 in Burkina Faso.

**Fig. 5:** High-risk births distribution by region among children born between 2003 and 2008 in Burkina Faso.

**Fig. 6:** Under-five mortality rates by region among children born between 2003 and 2008 in Burkina Faso.

### Multivariate analyses

#### Extent to which high-risk birth factors are associated with modern contraceptive use

After adjusting for all covariates, high birth parity was not a significant predictor of contraceptive use in rural areas, but it was a predictor in urban areas and at the national level (see Table 5). In urban areas, the odds of women with parity higher than three children using contraception was more than twice that of women with just one child (aOR=2.17, 95% CI: 1.10–4.27), while national aOR was 1.49 (95% CI: 1.02–2.17). Rural women who experienced a short birth spacing for the latest childbirth were more likely to use modern contraception (aOR=1.61, 95% CI: 1.20–2.17) afterwards than those who had births spaced by more than 35 months. In urban areas, the latter group of women reported significantly greater contraceptive use compared to women who had birth intervals of between 24 and 35 months. We did not find any significant association between modern contraceptive use and the mother's age at childbirth, after adjusting the odds ratios.

**Table 5:** Adjusted odds ratios from logistic regression models of modern contraceptive use by women in a union with a child born in the preceding 5 years in Burkina Faso

	Model 1 – rural area			Model 2 – urban area			Model 3 – nationwide		
			
Risk birth factors and other background characteristics	aOR	95% CI		aOR	95% CI		aOR	95% CI	
Mother's age at birth									
<18	0.82	(0.41–1.63)	ns	1.08	(0.33–3.57)	ns	0.91	(0.51–1.62)	ns
18–34	1.12	(0.78–1.61)	ns	1.42	(0.69–2.92)	ns	1.19	(0.86–1.66)	ns
35–49	1.00	(ref.)		1.00	(ref.)		1.00	(ref.)	
Parity/birth order									
1	1.00	(ref.)		1.00	(ref.)		1.00	(ref.)	
2–3	1.22	(0.85–1.76)	ns	1.88	(1.25–2.84)	***	1.42	(1.08–1.87)	**
>3	1.21	(0.78–1.89)	ns	2.17	(1.10–4.27)	**	1.49	(1.02–2.17)	**
Birth interval									
First birth	n/a			na			na		
9–23 months	1.61	(1.20–2.17)	***	0.94	(0.50–1.77)	ns	1.41	(1.07–1.85)	**
24–35 months	0.98	(0.79–1.21)	ns	0.70	(0.52–0.95)	**	0.89	(0.75–1.06)	ns
36+ months	1.00	(ref.)		1.00	(ref.)		1.00	(ref.)	
Missing	2.63		ns	0.67	(0.10–4.56)	ns	0.75	(0.09–6.06)	ns
Sons born within preceding 5 years									
0	1.00	(ref.)		1.00	(ref.)		1.00	(ref.)	
1	1.34	(1.07–1.67)	**	1.09	(0.77–1.56)	ns	1.24	(1.02–1.49)	**
2–3	1.16	(0.79–1.73)	ns	0.88	(0.47–1.64)	ns	1.06	(0.76–1.49)	ns
Daughters born within preceding 5 years									
0	1.00	(ref.)		1.00	(ref.)		1.00	(ref.)	
1	1.16	(0.91–1.47)	ns	1.11	(0.79–1.56)	ns	1.13	(0.93–1.38)	ns
2–3	1.20	(0.80–1.80)	ns	0.95	(0.49–1.86)	ns	1.09	(0.77–1.55)	ns
Time since last birth									
<1 year	0.20	(0.15–0.28)	***	0.26	(0.16–0.40)	***	0.22	(0.17–0.29)	***
1–2 years	0.83	(0.67–1.03)	*	0.75	(0.55–1.01)	*	0.81	(0.68–0.97)	**
3+ years	1.00	(ref.)		1.00	(ref.)		1.00	(ref.)	
Told about FP at health facility?									
No	1.00	(ref.)		1.00	(ref.)		1.00	(ref.)	
Yes	3.17	(2.52–3.99)	***	2.21	(1.63–3.00)	***	2.81	(2.34–3.38)	***
Did not visit health facility	0.76	(0.58–0.99)	**	0.77	(0.53–1.12)	ns	0.75	(0.60–0.93)	***
Postnatal check at health facility?									
No	1.00	(ref.)		1.00	(ref.)		1.00	(ref.)	
Yes	1.04	(0.80–1.36)	ns	1.19	(0.81–1.74)	ns	1.10	(0.89–1.37)	ns
Missing	1.01	(0.60–1.71)	ns	0.84	(0.43–1.63)	ns	0.98	(0.66–1.47)	ns
Location of residence									
Urban	–			–			1.26	(0.99–1.60)	*
Rural	–			–			1.00	(ref.)	
Administrative region									
Boucle du Mouhoun	0.38	(0.23–0.62)	***	1.34	(0.65–2.74)	ns	0.69	(0.46–1.03)	*
Cascades	0.36	(0.18–0.68)	***	0.71	(0.41–1.21)	ns	0.58	(0.36–0.93)	**
Centre	1.00	(ref.)		1.00	(ref.)		1.00	(ref.)	
Centre-Est	0.38	(0.21–0.67)	***	0.69	(0.44–1.09)	ns	0.60	(0.40–0.88)	***
Centre-Nord	0.44	(0.28–0.71)	***	0.82	(0.43–1.57)	ns	0.69	(0.46–1.03)	*
Centre-Ouest	0.36	(0.20–0.66)	***	0.78	(0.48–1.27)	ns	0.60	(0.38–0.94)	**
Centre-Sud	0.73	(0.48–1.12)	ns	0.92	(0.51–1.65)	ns	1.09	(0.77–1.55)	ns
Est	0.50	(0.31–0.82)	***	1.89	(0.75–4.79)	ns	0.85	(0.56–1.29)	ns
Hauts-Bassins	0.86	(0.54–1.39)	ns	1.17	(0.73–1.85)	ns	1.24	(0.86–1.80)	ns
Nord	0.40	(0.26–0.62)	***	0.96	(0.57–1.64)	ns	0.66	(0.46–0.95)	**
Plateau Central	0.45	(0.28–0.72)	***	0.54	(0.27–1.10)	*	0.67	(0.44–1.01)	*
Sahel	0.65	(0.33–1.28)	ns	1.25	(0.58–2.71)	ns	0.94	(0.53–1.67)	ns
Sud-Ouest	0.34	(0.12–1.01)	*	0.84	(0.45–1.57)	ns	0.64	(0.35–1.17)	ns
Wealth quintile									
Poorest	1.00	(ref.)		1.00	(ref.)		1.00	(ref.)	
Poorer	1.31	(1.01–1.69)	**	1.83	(0.95–3.51)	*	1.30	(1.02–1.66)	**
Middle	1.25	(0.96–1.61)	*	2.16	(0.97–4.84)	*	1.30	(1.02–1.66)	**
Richer	1.95	(1.48–2.56)	***	2.96	(1.59–5.51)	***	2.08	(1.62–2.68)	***
Richest	3.93	(2.71–5.70)	***	4.88	(2.55–9.32)	***	3.55	(2.63–4.80)	***
Educational attainment									
No education	1.00	(ref.)		1.00	(ref.)		1.00	(ref.)	
Primary	1.77	(1.38–2.28)	***	1.12	(0.82–1.53)	ns	1.48	(1.21–1.80)	***
Secondary or greater	5.53	(3.43–8.93)	***	2.24	(1.57–3.21)	***	3.00	(2.24–4.02)	***
Ethnicity									
Mossi	1.00	(ref.)		1.00	(ref.)		1.00	(ref.)	
Bobo	2.15	(1.43–3.23)	***	1.29	(0.82–2.03)	ns	1.88	(1.37–2.58)	***
Peulh	0.54	(0.33–0.87)	**	0.94	(0.52–1.69)	ns	0.62	(0.42–0.90)	**
Gourmantche	0.74	(0.47–1.16)	ns	0.58	(0.17–1.98)	ns	0.74	(0.49–1.10)	ns
Gourounsi	0.72	(0.38–1.37)	ns	1.51	(0.74–3.06)	ns	0.85	(0.53–1.34)	ns
Lobi/Dagara	1.53	(0.55–4.28)	ns	1.26	(0.69–2.29)	ns	1.28	(0.74–2.23)	ns
Bissa	1.30	(0.80–2.09)	ns	1.13	(0.65–1.95)	ns	1.24	(0.88–1.76)	ns
Other ethnicity	1.08	(0.68–1.72)	ns	0.92	(0.52–1.62)	ns	0.96	(0.67–1.36)	ns
Other country	0.71	(0.16–3.03)	ns	1.01	(0.40–2.55)	ns	0.87	(0.37–2.05)	ns
Religion									
Muslim	1.00	(ref.)		1.00	(ref.)		1.00	(ref.)	
Catholic	1.16	(0.92–1.46)	ns	1.44	(1.02–2.03)	**	1.28	(1.06–1.55)	**
Protestant	2.22	(1.54–3.21)	***	0.97	(0.59–1.58)	ns	1.78	(1.30–2.45)	***
Traditional	0.55	(0.37–0.82)	***	0.54	(0.24–1.19)	ns	0.56	(0.39–0.81)	***
Mother's age at time of survey	1.03	(0.91–1.18)	ns	0.95	(0.76–1.18)	ns	0.99	(0.89–1.11)	ns
Mother's age squared at time of survey	1.00	(1.00–1.00)	ns	1.00	(1.00–1.00)	ns	1.00	(1.00–1.00)	ns

* *p*<0.10,

** *p*<0.05,

*** *p*<0.01. ns, not significant; na, not applicable; aOR, adjusted odds ratio; CI, confidence interval; ref., value of reference.

#### Modern contraception use in relation to gender preference, time since childbirth, and contact with the health system

Overall, the analyses demonstrated that the number of daughters born and still alive in the 5 years preceding the time of survey was not associated with the probability of using modern contraception (see Table 5). However, in rural areas and nationally, women who had one son during the last 5 years were more likely (aOR=1.34, 95% CI: 1.07–1.67 in rural areas; aOR=1.24, 95% CI: 1.02–1.49 nationally) to use a contraceptive method than those without a male child.

The odds of using modern contraception were significantly lower for women who had given birth within the previous year (aOR=0.22, 95% CI: 0.17–0.29 nationally; aOR=0.20, 95% CI: 0.15–0.28 in rural areas; aOR=0.26, 95% CI: 0.16–0.40 in urban areas), as well as for women whose most recent childbirth was 1 to 2 years ago (aOR=0.81, 95% CI: 0.68–0.97 nationally).

Post-natal visits within 2 months of childbirth was not significant in predicting use of contraception. In contrast, whether the woman visited a health facility within the last 12 months and was told about FP was significantly associated (*p*<0.01), nationally as well as in urban and rural areas, with odds of using contraception, two to three times higher than that of women who were not told about FP at a health facility. Moreover, modern contraceptive use was lower for women who did not have a health facility visit within the last 12 months nationally and in rural areas.

#### Place of residence and cultural and socio-economic characteristics as determinants of modern contraception use

Modern contraceptive use was more likely in urban areas (aOR=1.26, 95% CI: 0.99–1.60) compared to rural areas in Burkina Faso (see Table 5). Considering the total population in each region, modern contraceptive use was lower in Centre-Est (aOR=0.60, 95% CI: 0.40–0.88), Centre-Ouest (aOR=0.60, 95% CI: 0.38–0.94), and Nord (aOR=0.66, 95% CI: 0.46–0.95), compared with the Centre region. After controlling for the urban/rural designation, modern contraceptive use was lower in the rural parts of most regions (excepting Hauts-Bassins, Sahel, and Centre-Sud) with significantly lower use in Centre-Ouest (aOR=0.36, 95% CI: 0.20–0.66), Centre-Est (aOR=0.38, 95% CI: 0.21–0.67), and Nord (aOR=0.40, 95% CI: 0.26–0.62). For the model using urban data, modern contraceptive use was not significantly different between regions.

Modern contraceptive use generally increased with increasing household wealth and women's education attainment in all three models – urban, rural, and national. For instance, the odds of using modern contraception were more than three times higher for women in the richest household wealth quintile compared to women in the poorest wealth quintile in all models. Similarly, contraceptive use was significantly higher in women with at least a secondary education compared to women with no education, while nationally the odds of a more educated woman using modern contraception were about three times higher than those of an uneducated woman. While both were statistically significant (*p<*0.01), it was noted that the odds ratio for women with at least a secondary education in rural areas (aOR=5.53, 95% CI: 3.43–8.93) was higher than that of women in urban areas (aOR=2.24, 95% CI: 1.57–3.21), suggesting that the relationship between education and contraception use may be stronger in rural than urban areas. It should be noted that only 1.8% of the rural women were identified as having secondary or higher education, compared with 22.8% in urban areas and 5.5% overall.

Women from the Bobo ethnic group were more likely to use modern contraception (aOR=1.88, 95% CI: 1.37–2.58 nationally) than women from the Mossi ethnic group (the reference ethnic group making up 51.6% of the total women in the dataset nationally). In contrast, women in the Peulh ethnic group were much less likely to use modern contraception (aOR=0.62, 95% CI: 0.42–0.90 nationally). Religious affiliation was also a significant predictor of modern contraceptive use nationally. Compared to the Muslim religion group (63.9% of the total women in the data set), Catholic (aOR=1.28, 95% CI: 1.06–1.55) and Protestant (aOR=1.78, 95% CI: 1.30–2.45) women were more likely to be users. Women associated with traditional religions were less likely to be using modern contraception (aOR=0.56, 95% CI: 0.39–0.81).

## Discussion

Even though modern contraceptive use represents one of the most studied issues in reproductive health, few things are known about its associations with birth risk and child mortality at both the national and sub-national levels. In this respect, our results clearly showed a strong relationship between modern contraceptive use and the distribution of high-risk births, as low contraceptive use was associated with the highest rates of birth risk and child mortality. Although these findings applied to most regions in Burkina Faso, the issue was more noticeable in the Sahel, Est, and Sud-Ouest regions. We also found a strong relationship between child mortality and factors for high-risk birth, such as short birth intervals (<24 months). This may indicate that programmes for improving the percentage of demand satisfied for modern methods of contraception in this country may result not only in a decline in unplanned pregnancies, but may also change the percentage and distribution of high-risk births. Improving modern contraceptive use can result in greater birth spacing and may lead to reductions in child mortality. This opportunity exists throughout Burkina Faso, as the percentage of demand for modern contraceptive methods satisfied was only 37.2% nationally, ranking between 25.2 and 53.2% in various regions in the 2010 DHS survey.

While women generally had a high level of knowledge about modern methods of contraception (98%) (2), the overall usage in 2010 was comparatively low at 15.4%, maybe a reflection of combined lack of demand and barriers in supply and demand. Similar to previous studies (32, 6, 12), we confirmed nevertheless that modern contraceptive use was more likely in urban areas compared to rural areas in Burkina Faso. In addition, there was no significant increase in mCPR, especially where the prevalence was already higher in 2003. Data from the next DHS in Burkina Faso may facilitate understanding of whether this was just a short-term stall in mCPR increase.

The observation that modern contraceptive use was higher among wealthier households in both urban and rural areas after controlling for factors such as education may reflect issues of supply, demand, or affordability. Given DHS limitations for examining institutional and economic barriers to usage, further investigations are needed to determine what is driving these issues. A key insight from this research involves the critical relationship between women's interactions with the health system and the use of modern contraception. The importance of the health system was emphasised by the observation that the odds of using modern contraceptives were more than doubled for women who had been informed about FP during a health facility visit in the last 12 months, as compared to women who had visited a facility but were not informed. This difference may also reflect the current model of delivery of FP services, which are mainly provided through health facilities as opposed to being community-based. The results suggest, however, that programming that integrates FP information into antenatal and postnatal care health facility visits is likely to have a substantial impact on contraceptive prevalence rates. In the light of that, it should be worthwhile to take account of and leverage this integration opportunity in future programmes.

The results suggested that ethnic group and religious affiliation were significant cultural factors in predicting the use of modern contraception. After controlling for other covariates, lower contraceptive use was observed for women belonging to the Peulh ethnic group and among those who were Muslim or in traditional/animist religions. Qualitative analysis of data on perceptions, values, and cultural beliefs may need to be performed to better understand the barriers and the potential unmet needs for FP among those populations. It is important that FP messaging be sensitive to the diverse cultural and ethnic groups within Burkina Faso.

As previously mentioned, high-risk births may represent a potential increased mortality risk to both mothers and children, which we hoped to address specifically. However, available data did not permit analysis of the relationship between mCPR or birth risk and maternal mortality, since there were no detailed data on women who died. The key limitations of this study related to the data source itself, the DHS and, more generally, retrospective surveys. Much of the data for this survey was based on recall, including the enumeration of births, reporting of early neonatal deaths, recollection of timing of facility visits, and corresponding exposure to FP information. Recall bias and error may have resulted in underreporting of early neonatal death cases or childbirths, or in inaccuracy of timing related to reported events. In addition to recall issues, this analysis focused on births that occurred within the 5 years preceding the survey. As a result, it is possible that changes occurred to the mother's individual, household, or ecological characteristics during the time gap between the survey and the birth being used for the analysis. Specifically, one could envision situations such as the birth being referenced occurring during a time when the mother was living in a rural area but, at the time of the survey, she was recorded as living in an urban area. Future research could explore the influence of this issue through sensitivity analysis around the time window used for previous births.

## Conclusion

In Burkina Faso, modern contraception prevalence remains low, despite a high level of knowledge about FP. Moreover, the regional heterogeneity highlighted can reflect an issue of equity in terms of FP coverage. Increasing the demand satisfied by modern contraception would reduce the percentage of births that are high risk and probably result in reducing child mortality through a variety of mechanisms, including achieving a lower percentage of short-interval births. Contact with the health system is a critical opportunity for promoting modern contraceptive use and, more broadly, for FP programming. FP programming that is integrated into routine care as well as antenatal and postnatal visits is likely to have significant impact, enabling Burkina Faso to reduce the unmet need for FP and enabling women to fully exercise their reproductive rights.

## Supplementary Material

Trends and patterns of modern contraceptive use and relationships with high-risk births and child mortality in Burkina Faso Click here for additional data file.

## References

[1] WHO (2014). Trends in maternal mortality: 1990 to 2013 estimates by WHO, UNICEF, UNFPA, The World Bank and the United Nations Population Division.

[2] INSD, ICF International (2012). Enquête Démographique et de Santé et à Indicateurs Multiples du Burkina Faso 2010.

[3] Liu L, Oza S, Hogan D, Perin J, Rudan I, Lawn JE (2015). Global, regional, and national causes of child mortality in 2000–13, with projections to inform post-2015 priorities: an updated systematic analysis. Lancet.

[4] Fotso JC, Cleland J, Mberu B, Mutua M, Elungata P (2013). Birth spacing and child mortality: an analysis of prospective data from the Nairobi Urban Health and Demographic Surveillance System. J Biosoc Sci.

[5] Kembo J, Van Ginneken JK (2009). Determinants of infant and child mortality in Zimbabwe: results of multivariate hazard analysis. Demogr Res.

[6] Kirk D, Pillet B (1998). Fertility levels, trends, and differentials in sub-Saharan Africa in the 1980s and 1990s. Stud Fam Plann.

[7] Caldwell JC, Caldwell P (1987). The cultural context of high fertility in sub-Saharan Africa. Popul Dev Rev.

[8] Shapiro D, Gebreselassie T (2011). Fertility transition in sub-Saharan Africa: falling and stalling. Afr Popul Stud.

[9] Bongaarts J, Frank O, Ron L (1984). The proximate determinants of fertility in sub-Saharan Africa. Popul Dev Rev.

[10] Hubacher D, Mavranezouli I, McGinn E (2008). Unintended pregnancy in sub-Saharan Africa: magnitude of the problem and potential role of contraceptive implants to alleviate it. Contraception.

[11] Cleland JG, Ndugwa RP, Zulu EM (2011). Family planning in sub-Saharan Africa: progress or stagnation?. Bull World Health Organ.

[12] Lesthaeghe R (2014). The fertility transition in sub-Saharan Africa into the 21st century.

[13] Cleland J, Conde-Agudelo A, Peterson H, Ross J, Tsui A (2012). Contraception and health. Lancet.

[14] Stover J, Ross J (2013). Changes in the distribution of high-risk births associated with changes in contraceptive prevalence. BMC Public Health.

[15] Cramer JC (1987). Social factors and infant mortality: identifying high-risk groups and proximate causes. Demography.

[16] Adebowale SA, Palamuleni ME (2014). Determinants of unmet need for modern contraception and reasons for non-use among married women in rural areas of Burkina Faso. Etude Popul Afr.

[17] Campbell M, Sahin-Hodoglugil NN, Potts M (2006). Barriers to fertility regulation: a review of the literature. Stud Fam Plann.

[18] Konje JC, Ladipo OA (1999). Barriers to uptake and use of modern methods of contraception in developing countries. Int J Gynecol Obstet.

[19] Hamid S, Stephenson R (2006). Provider and health facility influences on contraceptive adoption in urban Pakistan. Int Fam Plann Perspect.

[20] Asekun-Olarinmoye EO, Adebimpe WO, Bamidele JO, Odu OO, Asekun-Olarinmoye IO, Ojofeitimi EO (2013). Barriers to use of modern contraceptives among women in an inner city area of Osogbo metropolis, Osun state, Nigeria. Int J Women's Health.

[21] Ochako R, Mbondo M, Aloo S, Kaimenyi S, Thompson R, Temmerman M (2015). Barriers to modern contraceptive methods uptake among young women in Kenya: a qualitative study. BMC Public Health.

[22] Stephenson R, Baschieri A, Clements S, Hennink M, Madise N (2007). Contextual influences on modern contraceptive use in sub-Saharan Africa. Am J Public Health.

[23] Kragelund Nielsen K, Nielsen SM, Butler R, Lazarus JV (2012). Key barriers to the use of modern contraceptives among women in Albania: a qualitative study. Reprod Health Matters.

[24] INSD (2009). Recensement général de la population et de l'habitation 2006 (RGPH 2006) – Rapport d'analyse du thème 2: Etat et structure de la population.

[25] Rutstein SO, Rojas G (2006). Guide to DHS statistics.

[26] Cecatti JG, Correa-Silva EPB, Milanez H, Morais SS, Souza JP (2008). The associations between inter-pregnancy interval and maternal and neonatal outcomes in Brazil. Matern Child Health J.

[27] Conde-Agudelo A, Rosas-Bermúdez A, Kafury-Goeta A (2006). Birth spacing and risk of adverse perinatal outcomes: a meta-analysis. J Am Med Assoc.

[28] Rutstein SO (2005). Effects of preceding birth intervals on neonatal, infant and under-five years mortality and nutritional status in developing countries: evidence from the demographic and health surveys. Int J Gynecol Obstet.

[29] StataCorp (2013). Stata: Release 13 statistical software.

[30] StataCorp (2013). Stata survey data reference manual release 13.

[31] Verma V, Betti G (2011). Taylor linearization sampling errors and design effects for poverty measures and other complex statistics. J Appl Stat.

[32] Cohen B (1998). The emerging fertility transition in sub-Saharan Africa. World Dev.

# Sub article: Trends in contraceptive use and distribution of births with demographic risk factors in Ethiopia: a sub-national analysis

**DOI:** 10.3402/gha.v8.29720

**Published:** 2015-11-09

**Authors:** Solomon Shiferaw, Muna Abdullah, Yared Mekonnen, Abdoulaye Maïga, Akanni Akinyemi, Agbessi Amouzou, Howard Friedman, Aluisio J. D. Barros, Sennen Hounton

**Affiliations:** 1 Department of Reproductive Health and Management, School of Public Health, Addis Ababa University, Addis Ababa, Ethiopia; 2 UNFPA – East and Southern Africa Regional Office, Johannesburg, South Africa; 3 Mela Research PLC, Addis Ababa, Ethiopia; 4 Institut Supérieur des Sciences de la Population, Ouagadougou University, Ouagadougou, Burkina Faso; 5 Department of Demography and Social Statistics, Obafemi Awolowo University, Ile Ife, Nigeria; 6 UNICEF, New York, NY, USA; 7 United Nations Population Fund (UNFPA), New York, NY, USA; 8 Federal University of Pelotas, Capão do Leão, Brazil

**Keywords:** Ethiopia, distribution of birth risks, modern contraceptive use, total fertility

## Abstract

**Background:**

Evidence shows that family planning contributes to the decline in child mortality by decreasing the proportions of births that are considered high risk. The main objective of the present analysis was to examine the trends in use of modern contraceptives and their relationship with total fertility rate (TFR) and distribution of births by demographic risk factors as defined by mother's age, birth interval, and birth order at the sub-national level in Ethiopia.

**Design:**

Analyses used data from three Demographic and Health Surveys in Ethiopia (2000, 2005, and 2011), which are nationally representative data collected through questionnaire-based interviews from women 15–49 using a stratified, two-stage cluster sampling. First, we examined the trends of and relationship between TFR (in the 3 years before each survey) and modern contraceptive use among currently married women in all administrative regions over the time period 2000–2011 using linear regression analysis. We also examined the relationship between birth risks and under-five mortality using the no-risk group as a reference. Finally, multiple logistic regression analysis was performed to estimate the relationship between the effect of being a resident in one of the regions and having an avoidable birth risk (which includes births to mothers younger than 18 and older than 34 years, birth interval of less than 24 months and birth order higher than third) after adjusting for select covariates including wealth, educational status, residence, religion and exposure to family planning information.

**Results:**

Sub-national-level regression analysis showed an inverse relationship between modern contraceptive use among married women and the TFR, with an average decrease of TFR by one child per woman associated with a 13 percentage point increase in modern contraceptive use between 2000 and 2011. A high percentage of births in Ethiopia (62%) fall in one of the risk categories (excluding first births), with wide regional variation from 55% in Gambela to 72% in the Somali region. The multivariate analysis showed women living in the Somali, Afar and Benishangul-Gumuz regions had significantly higher odds of having avoidable birth risk compared to those in Addis Ababa after controlling for observed covariates. The trend analysis showed there was a significant drop in the proportion of births from women above 34 years between 2000 and 2011. There was no significant decline in births to women less than 18 years between 2000 and 2011.

**Conclusions:**

A majority of births in Ethiopia fall in one of the risk categories, with substantial region-to-region variation in the percentage of births with avoidable risk factors, Somali and Afar having the highest burden. The analysis indicated that births in the three regions had significantly higher odds of having one of the avoidable risk factors compared to Addis Ababa, and we suggest family planning programmes need to identify differentials of modern contraceptive use at the sub-national level in order to better address coverage and equity issues.

**Paper context**

Family planning contributes to the decline in child mortality by decreasing the proportions of births that are considered high risk. A majority of births in Ethiopia (62%) fall in one of the risk categories, with substantial region-to-region variation. Births in Somali, Afar, and Benishangul-Gumuz regions had significantly higher odds of having one of the avoidable risk factors compared to Addis Ababa. Family planning programmes need to identify differentials of modern contraceptive use at the sub-national level in order to better address coverage and equity issues.

Family planning programmes contribute to improving child survival by reducing the proportion of ‘high-risk’ births (1–5). Generally, the three demographic groups of children who are considered to be at a higher risk of mortality are those born to very young mothers (less than 18 years of age), those born to older women (past 40 years of age), and those born too soon after a previous birth (less than 2 years) (2).

Evidence shows that there is a strong negative correlation between levels of contraceptive use and levels of high-risk births and infant mortality. High-risk births account for large proportions of births in developing regions. For example, narrowly spaced births and births to the youngest and oldest mothers account for up to 25 and 36% of births, respectively, in many sub-Saharan countries. Similarly, births to adolescents account for at least 15% of births in sub-Saharan African and Latin American countries (5). Like many low-income countries, Ethiopia has a high adolescent birth rate at 79 per 1,000, with more than one-third (34%) of women 20–49 years having given birth by age 18 and 20% of births occurring within 24 months of the previous birth (6).

With an average annual increase of modern contraceptive prevalence rate among married women (mCPR) of nearly 2 percentage points since 2000, the country has registered significant progress (6). Modern contraceptive use among currently married women nearly quadrupled from 6.3% in 2000 to 27.3% in 2011.

However, with an unmet need of 25% and demand satisfied only for 53% of women in 2011, there is substantial unfulfilled demand for modern contraceptives. In addition, the data show that progress is unevenly distributed. For example, the mCPR in the Somali region (which is the region of Ethiopia bordering Somalia) in 2011 was 3.8%, a figure well below the national average 11 years earlier. The capital city, Addis Ababa, has the highest mCPR among married women at 56%, followed by Gambela and Amhara regions at 33% each. Unmet need is highest in Oromia at 30% and lowest in Addis Ababa at 11%. With unmet need of 16% and met need of 10%, Afar has the lowest demand at 26%, compared to Addis Ababa, which has a demand of 73% (6).

The same region-to-region variation exists with regard to total fertility rate (TFR), which is the average number of children that would be born per woman if all women lived to the end of their childbearing years and gave birth to children according to a given fertility rate at each age (7). The TFR in 2011 ranged from 1.5 in Addis Ababa (below replacement level) and 4.0 in the Gambela region to 7.1 in the Somali region, while the national average was 4.8. Indeed, the Somali region saw an increase in TFR between 2000 (5.7) and 2011 (7.1) (6).

The rapid increase in access to and use of contraceptives was also associated with decreasing infant and under-five mortality rates, partly attributable to the launch of the health extension programme, which has deployed more than 30,000 government-salaried health extension workers in every sub-district (or *kebele*) of the country (8–10).

The aim of the present analysis is to examine 1) the trends in the use of modern contraceptives and their relationship with TFR, 2) distribution of birth risk, 3) trends in birth risk categories across regions, and 4) the relationship between the distribution of birth risk and under-five mortality in Ethiopia.

By showing the relative contribution of contextual factors (mainly residence in one of the regions) in reducing avoidable birth risks, the analysis will generate crucial information that will be useful to advocate for prioritisation of family planning programmes, particularly in regions with performance below the national average.

## Methods

### Design and study period

The analysis uses data from three Demographic and Health Surveys (DHS) in Ethiopia (2000, 2005, and 2011), which are repeat cross-sectional surveys by design. DHS collects questionnaire interview-based nationally representative data from women 15–49 using a stratified, two-stage cluster sampling that allows for specific indicators, such as modern contraceptive use, to be collected. By design, the survey allows the estimation of regional-level indicators. Questions asked include, among others, background characteristics such as age and education, as well as information about key indicators of maternal and child health including birth history, childhood mortality, and use of family planning methods. The surveys are carried out under the aegis of the Ministry of Health and are implemented by the Central Statistical Agency (CSA). ICF International provides technical assistance as well as funding to the project through the MEASURE DHS project, a project funded by the US Agency for International Development providing support and technical assistance in the implementation of population and health surveys in countries worldwide. The data are freely available from the CSA or the MEASURE DHS project (6, 11, 12).

First, we examined the trends of and relationship between TFR (in the 3 years before the survey) and mCPR (including method mix) in the 11 administrative regions over the time period 2000–2011 using linear regression analysis. We also examined the relationship between birth risks and under-five mortality by calculating the risk ratio with 95% confidence interval, using the ‘no-risk’ group (which is actually defined as births not associated with any specific risk) as a reference.

### Data processing

The main outcome of interest in this study was the percentage of births with avoidable birth risk for births in the 5 years preceding each survey. Birth risk categories were defined by three main variables: mother's age at birth, grouped into less than 18 years, 18–34 years, and 35–49 years; preceding birth interval, which is the length in months between previous birth and the index child, grouped into less than 18 months, 18–23 months, and 24 or more months; and birth order, grouped into first births, second or third births, and fourth or greater births. As described by Ross and Stover (2), birth risk categories include the following 11 mutually exclusive groups of children based on the percentage of children born in the 5 years preceding the survey. *Avoidable birth risk* refers to categories numbered 3 to 11 below (i.e. excluding first births in the optimal age range of 18–34, which is considered an unavoidable risk).

1. Not in any risk category
2. First birth to mother 18–34 years age
3. Mother's age <18 years
4. Mother's age >34 years
5. Birth interval <24 months
6. Birth order >3
7. Mother's age <18 years and birth interval <24 months
8. Mother's age >34 years and birth interval <24 months
9. Mother's age >34 years and birth order >3
10. Birth interval <24 months and birth order >3
11. Mother's age >34 years, birth interval <24 months and birth order >3

We used births in the preceding 5 years to have sufficient numbers for the analysis of births by all demographic risk factors (maternal age, inter-birth interval, and higher order births).

Independent variables include region (Addis Ababa, Afar, Amhara, Oromia, Somali, Benishangul-Gumuz, Southern Nations and Nationalities People [SNNP], Tigray, Harari, Gambela, and Dire Dawa), wealth quintile, educational status (no education, primary, secondary, higher) residence (urban, rural), and discussion about family planning in health facilities (yes, no).

Modern contraceptive use in this study refers to current use of modern family planning methods among currently married (or in union) women, which include pills, intrauterine devices, injectables, condoms, female sterilisation, vasectomy, implants, and the lactational amenorrhoea method.

Finally, multiple logistic regression analysis was performed to estimate the relationship between the effect of being a resident in one of the regions and having avoidable birth risk, after adjusting for select covariates including wealth, educational status, residence, and exposure to family planning information. Stata 12.1 was used for data management and analysis.

## Results

### Trends and regional variation in TFR and mCPR among married women and women in union

Overall, the Ethiopian DHS in 2000, 2005, and 2011 surveyed 15,367, 14,070, and 16,515 women aged 15–49 in 14,072, 13,721, and 16,702 households, respectively, with close to 70% of the sample coming from rural areas in all rounds. Distribution of the sample by region is shown in Table 1.

**Table 1:** Trends in TFR for births in the preceding 3 years by region and residence across time, Ethiopia DHS 2000 to 2011

Characteristic	TFR in 2000 (95% CI)	*n*	TFR in 2011 (95% CI)	*N*	Absolute change DHS 2000–2011
Total	5.5 (5.3, 5.7)	15,306	4.8 (4.5, 5.1)	16,438	−0.7
Residence					
Urban	3.0 (2.6, 3.4)	4,530	2.6 (−0.6, 5.9)	5,310	−0.4
Rural	6.0 (5.8, 6.3)	10,776	5.5 (5.1, 5.8)	11,128	−0.5
Region					
Addis Ababa^a^	1.8 (1.2, 2.5)	2,010	1.5 (0.9, 2.1)	1,738	−0.3
Dire Dawa^a^	3.5 (2.6, 4.3)	1,048	3.4 (2.6, 4.0)	1,086	−0.1
Harari	4.2 (3.1, 5.3)	906	3.8 (3.1, 4.6)	1,094	−0.4
Gambela	4.4 (2.3, 6.5)	873	4.0 (2.9, 5.0)	1,122	−0.4
Afar	4.4 (3.4, 5.4)	855	4.6 (4.1, 5.1)	1,283	0.2
Benishangul-Gumuz	5.0 (4.3, 5.8)	991	5.2 (4.3, 6.1)	1,257	0.2
Somali	5.1 (3.9, 6.3)	837	7.1 (5.9, 8.2)	913	2.0
Tigray	5.3 (4.7, 6.0)	1,300	5.0 (4.3, 5.6)	1,717	−0.4
Amhara	5.5 (5.0, 6.0)	1,903	4.2 (3.7, 4.6)	2,077	−1.4^b^
SNNP	5.6 (5.2, 5.9)	2,017	4.9 (4.3, 5.4)	2,027	−0.7
Oromia	6.1 (5.6, 6.5)	2,566	5.6 (4.9, 6.3)	2,124	−0.4

a City administration councils;

b statistically significant. *n*=number of women (un-weighted). TFR, total fertility rate; CI, confidence interval; DHS, Demographic and Health Survey; SNNP, Southern Nations and Nationalities People.

As shown in Table 1, the TFR in the 3 years preceding each survey declined significantly by 0.7 children per woman, from 5.5 (95% CI: 5.3, 5.7) to 4.8 (95% CI: 4.5, 5.1) between 2000 and 2011 at the national level. The region with the highest and statistically significant drop in TFR was Amhara (by 1.4 children per woman). TFR also showed relatively higher decline in Tigray (by 0.4 children per woman) and SNNP (by 0.7 child per woman), though not significantly different. On the other hand, the Somali region saw a reverse trend, with TFR increasing by two children per woman during the same period, although marginally significant (TFR in 2000=5.1, 95% CI: 3.9, 6.3 vs. TFR in 2011=7.1, 95% CI: 6.0, 8.2). There was no statistically significant change in TFR for the rest of the regions.

Modern contraceptive use among currently married women significantly increased by 21 percentage points, from 6.3 to 27.3% (*p*<0.05). As expected, the region with the highest drop in TFR (Amhara) also showed the highest increase in mCPR in 2000–2011, at 26.4 percentage points, while the Somali and Afar regions had the smallest increases in mCPR, at 1.4 and 1.7 percentage points, respectively, showing no statistically significant change over time. The changes in mCPR in Dire Dawa and Harari regions were not statistically significant, apparently because of small sample size, as detailed in Table 2.

**Table 2:** Trends in mCPR by region and residence across time, Ethiopia DHS 2000 to 2011

Characteristic	mCPR in 2000 (95% CI)	*n*	mCPR in 2011 (95% CI)	*n*	Absolute change DHS 2000–2011
Total	6.3 (5.2, 7.6)	9,380	27.3 (24.9, 29.9)	10,204	21.0^a^
Residence					
Urban	28.3 (24.4, 32.5)	1,843	49.5 (45.6, 53.3)	2,422	21.2^a^
Rural	3.3 (2.6, 4.1)	7,537	22.5 (20.1, 25.1)	7,782	19.2^a^
Region					
Addis Ababa^b^	34.3 (30.7, 38.1)	670	56.3 (52.4, 60.2)	634	22.0^a^
Dire Dawa^b^	23.5 (12.6, 27.7)	526	31.7 (24.6, 39.8)	626	8.2
Harari	19.0 (12.6, 27.7)	482	31.5 (25.4, 38.3)	635	12.5
Gambela	12.3 (7.8, 18.9)	656	33.2 (24.2, 43.6)	768	20.9^a^
Afar	7.4 (3.9, 13.6)	619	9.1 (5.4, 14.8)	960	1.7
Benishangul-Gumuz	8.5 (5.8, 12.3)	711	26.3 (19.9, 33.9)	904	17.8^a^
Somali	2.4 (0.5, 10.3)	569	3.8 (1.6, 8.6)	664	1.4
Tigray	9.3 (5.5, 15.3)	868	21.2 (17.1, 26.0)	984	11.9^a^
Amhara	6.6 (4.7, 9.3)	1,315	33.0 (28.6, 37.8)	1,331	26.4^a^
SNNP	5.0 (3.0, 8.3)	1,312	24.7 (20.1, 29.9)	1,295	19.7^a^
Oromia	4.3 (2.8, 6.6)	1,652	24.9 (20.6, 29.8)	1,403	20.6^a^

a Statistically significant;

b city administration councils. *n*=number of women (un-weighted). mCPR, modern contraceptive prevalence rate; CI, confidence interval; SNNP, Southern Nations and Nationalities People; DHS, Demographic and Health Survey.

### Trends in the distribution of method mix

In terms of method mix, the 3-month injectable (medroxyprogesterone acetate) is the most commonly used modern contraceptive method in Ethiopia, accounting for 72.6% of the method mix in 2011, followed by implants and pills at 12 and 7.4%, respectively. Trend analysis showed that implant use increased (from 1 to 12%) while pill use decreased (from 31 to 7.4%), as did traditional methods (from 1.7 to 1.3%). However, the pattern is slightly different in the Somali region, where pill use is as high as 20% of the method mix and injectables constituted 47% in 2011. Details are shown in Supplementary Table 1.

### Relationship between modern contraceptive use and TFR

As shown in Fig. 1, there was a significant negative linear relationship [TFR=−0.08235 (mCPR)+6.169; *p*<0.001] between the levels of modern contraceptive use among married women and TFR across the different regions of Ethiopia between 2000 and 2011. The analysis suggests that the TFR declined on average by one child per woman for every increase in the mCPR of about 13 percentage points. It also shows that the TFR would be around 6.2 if there were no modern contraceptive use.

**Fig. 1:** Relationship between changes in mCPR and TFR for all regions between 2000 and 2011.

### Levels and trends of birth risk categories for births in the preceding 5 years by region

As shown in Fig. 2, the proportion of births with avoidable risk (any risk factor other than first birth) was highest in Somali at 72% and Afar at 68%, followed by Benishangul-Gumuz (66%), Oromia (63%), and Amhara (62%). Addis Ababa and Dire Dawa (both cities with better access to health services) had lower percentages of births with any risk factors, at 34 and 54%, respectively. Interestingly, Gambela (a relatively underdeveloped region) also had a lower percentage of births with avoidable birth risk, at 55%.

**Fig. 2:** Distribution of births in the preceding 5 years by risk factor and TFR across regions, Ethiopia DHS2011.

As Fig. 3 shows, the most common single risk factors in 2011 were narrowly spaced births (22%) and births to mothers older than 34 years of age (21%), followed by births to mothers younger than 18 years of age (11%). Birth risk associated with higher parity (birth order greater than three) was the least common, at 8%. The main single risk factor that was consistently higher in the two regions with higher fertility (Somali and Afar) compared to the national average was the proportion of births with short birth interval, at 40 and 32%, respectively.

**Fig. 3:** Distribution of births in the preceding 5 years by single risk factor, Ethiopia DHS 2011.

Addis Ababa had the lowest percentage of births with short birth interval at 10%, followed by Tigray (15%) and Amhara (15%). As expected, Addis Ababa and Dire Dawa (both city administrations) also had higher proportions of first births, at 38 and 23%, respectively, compared to the national average of 15%.

Supplementary Table 2 shows that the proportion of births to mothers older than 34 years was the only single risk factor that showed a statistically significant drop between 2000 and 2011 at the national level, moving from 9.4% (95% CI: 8.7, 10.0) to 7.6% (95% CI: 7.1, 8.0). Looking at regional variations, there was a significant decline in the percentage of births with less than a 24-month birth interval in Addis Ababa, from 17.2% (95% CI: 14.8, 20.0) to 9.9% (95% CI: 7.3, 13.4), while the percentage of first births increased significantly from 26.5% (95% CI: 23.3, 30.1) to 37.9 (95% CI: 32.9, 43.2). It is important to note that the Gambela and Amhara regions had a significantly higher percentage of births to mothers younger than 18 years, at 15.6% (95% CI: 12.7, 19.0) and 15.2% (95% CI: 14.1, 16.3), respectively, compared to the national average of 11% (95% CI: 10.4, 11.8) in 2011.

### Levels and trends in distribution of births in the preceding 5 years with avoidable risk

As shown in Table 3, the Somali region had the highest proportion of avoidable birth risk (82.5%) in 2011, compared to the national average of 72.6%. While the changes were not statistically significant, the Somali and Afar regions showed a slight increase in the proportion of avoidable birth risk from 2000 to 2011.

**Table 3:** Trends in proportion of births in the preceding 5 years for which the birth risk is avoidable by region, Ethiopia DHS 2000 to 2011

	2000			2011		
		
Region	*n*	%	95% CI	*n*	%	95% CI
Tigray	1,365	68.8	(67.0, 70.7)	1,984	68.1	(66.1, 70.0)
Afar	192	74.2	(71.2, 77.1)	2,046	78.1	(75.9, 80.2)
Amhara	6,037	71.3	(69.7, 72.7)	2,443	70.6	(68.3, 72.8)
Oromia	8,761	75.3	(73.5, 77.0)	2,914	74.2	(72.3, 76.1)
Somali	288	81.6	(79.8, 83.3)	2,024	82.5	(80.9, 84.0)
Benishangul-Gumuz	204	73.4	(70.9, 75.8)	1,857	75.7	(73.1, 78.2)
SNNP	4,411	71.5	(69.8, 73.2)	2,868	72.4	(70.7, 73.9)
Gambela	40	66.5	(63.3, 69.6)	1,241	67.1	(64.3, 69.7)
Harari	42	78.3	(74.5, 81.6)	913	74.2	(69.9, 78.1)
Addis Ababa^a^	190	65.0	(61.6, 68.3)	240	54.2	(46.9, 60.8)^b^
Dire Dawa^a^	54	70.4	(66.7, 73.8)	903	71.8	(66.1, 74.6)
Total	22,000	72.8	(71.9, 73.7)	19,433	72.6	(71.5, 73.7)

a City administration councils;

b statistically significant. CI, confidence interval; SNNP, Southern Nations and Nationalities People; DHS, Demographic and Health Survey.

Our analysis did not show a significant decline in the distribution of avoidable birth risk distribution within regions, except in Addis Ababa, where it dropped from 70.9% (95% CI: 68.7, 73.1) in 2000 to 61.1% (95% CI: 57.6, 64.5) in 2011.

### Birth risk categories and under-five mortality

As shown in Table 4, among the different birth risk categories, the highest risk ratio for under-five mortality was for children who had the combined risk of short birth intervals and mothers under age 18 and/or over the age of 34, compared to the reference group of births with no risk factor, although there fewer cases in those categories. Among the single risk factors, births to mothers aged less than 18 (adolescent pregnancy, which often happens due to child marriage) and births with an interval of less than 24 months had higher odds of under-five mortality (risk ratio=1.77, 95% CI: 1.63, 1.92). The risk ratio represents the ratio of the proportion of births in the last 5 years in each risk category that had died to the proportion dead among births not in any risk category (reference category).

**Table 4:** Risk ratios for under-five mortality by distribution of birth risks in the 5 years preceding the survey, Ethiopia DHS 2011

Birth risk category	Number of births	Number of deaths	Under-five mortality	Risk ratio (95% CI)
Not in any risk category (reference)	37,738	388	76.9	1.0
Mother's age >34 and birth order >3	19,927	235	86.4	1.11 (1.01, 1.22)*
Birth order >3	40,918	482	87.7	1.13 (1.02, 1.23)*
First birth to mothers age 18–34	22,448	275	88.7	1.14 (1.04, 1.25)*
Mother's age >34	961	17	118.0	1.48 (1.36, 1.61)*
Birth interval <24 months and birth order >3	18,133	500	197.3	2.30 (2.13, 2.49)*
Mother's age <18	11,454	223	146.7	1.77 (1.63, 1.92)*
Birth interval <24	12,970	257	144.7	1.77 (1.63, 1.92)*
Mother's age >34 and birth interval <24 months and birth order >3	5,122	121	170.2	2.04 (1.88, 2.20)*
Mother's age <18 and birth interval <24 months	1,606	45	211.9	2.45 (2.26, 2.65)*
Mother's age >34 and birth interval <24 months	65	3	325.0	3.43 (3.19, 3.70)*

### Multivariate analysis: determinants of avoidable risk births

Table 5 presents the relationship between avoidable birth risk and region after adjusting for selected covariates including wealth quintile, educational status (no education, primary, secondary, higher), residence (urban, rural), discussion about family planning in health facilities (yes, no), and religion (Christian, Muslim, traditional/other).

**Table 5:** Results from multiple logistic regression analysis of predictors of avoidable birth risk for births in the preceding 5 years, Ethiopia DHS 2011

		95% CI	
		
Characteristics	AOR	Lower limit	Upper limit
Region (Addis Ababa^a^ – ref)			
Afar	1.68	1.11	2.53
Amhara	1.00	0.69	1.44
Oromia	1.18	0.82	1.69
Somali	2.16	1.33	3.51
Benishangul-Gumuz	1.48	1.01	2.17
SNNP	1.18	0.82	1.70
Gambela	1.12	0.78	1.61
Harari	1.28	0.84	1.93
Tigray	0.97	0.67	1.40
Dire Dawa^a^	1.10	0.74	1.65
Women's education (no education – reference)			
Primary	0.84	0.71	1.00
Secondary	0.59	0.40	0.88
Tertiary	0.51	0.28	0.91
Told of family planning at health facility (*yes* – reference)			
No	1.18	1.03	1.35
Residence (urban – ref)			
Rural	1.54	1.19	1.98

a City administration councils. AOR, adjusted odds ratio (adjusted for wealth quintile and religion); CI, confidence interval; SNNP, Southern Nations and Nationalities People; DHS, Demographic and Health Survey.

As shown in Table 5, births in the Somali region (adjusted odds ratio [AOR]=2.16, 95% CI: 1.33, 3.51), Afar region (AOR=1.68, 95% CI: 1.11, 2.53), and Benishangul-Gumuz region (AOR=1.48, 95% CI: 1.01, 2.17) had significantly higher likelihoods of having avoidable risk compared to the reference region, Addis Ababa. Similarly, rural residence was significantly associated with avoidable birth risk (AOR=1.54; 95% CI: 1.19, 1.98). Women with any form of formal education (primary, secondary, or tertiary) had significantly lower odds of having avoidable risk, with the highest reduction for women with tertiary education (AOR=0.51, 95% CI: 0.28, 0.91). Women who reported not having had any discussion about family planning in health facilities were also significantly more likely to have avoidable birth risks (AOR=1.18, 95% CI: 1.03, 1.35). Wealth quintile and religion were not associated with avoidable birth risk.

## Discussion

Over the 11-year study period, a majority of births in Ethiopia fell into one of the risk categories, with substantial region-to-region variation, the Somali and Afar regions having the highest burden. The analysis indicated that births in the Somali, Afar, and Benishangul-Gumuz regions had significantly higher odds of having one of the avoidable risk factors compared to Addis Ababa. This is consistent with previous studies showing low contraceptive use in Somali and Afar regions (13–15).

The present analysis suggests that the TFR declined on average by one child per woman for every increase in the mCPR of about 13 percentage points, which is lower compared to developing countries as a whole (average decrease of TFR by one child per woman for every increase in the mCPR of 17 percentage points) (2). This difference may indicate that factors other than contraception (such as better access to education and health services) are playing important roles in bringing down fertility. The observed inverse relationship between changes in TFR and mCPR points to the fact that regions with low modern contraceptive use have higher fertility, which in turn is associated with higher prevalence of births with risk factors (2).

The majority of births in Ethiopia (62%) fall into one of the high-risk categories (excluding first births), compared to the average for developing countries of 56% (2). The most common single risk factor is short birth interval (less than 24 months), which is particularly true for the Somali and Afar regions, where nearly 4 and 3 out of 10 births, respectively, have birth intervals of less than 24 months.

The trend analysis showed there was a significant drop in the proportion of births to mothers older than 34 years between 2000 and 2011 at the national level, while there was a significant decrease in the percentage of births with short birth intervals in Addis Ababa. There was no significant decline in births to women younger than 18 years between 2000 and 2011, so this remains an important risk factor, particularly in regions such as Amhara and Gambela where child marriage is a significant problem (16). Our findings add to the evidence that preventing child marriage and offering adolescents the possibility to protect themselves from unplanned pregnancy could contribute to reducing high-risk births and under-five mortality.

On the other end of the spectrum are cities like Addis Ababa and Dire Dawa, where the proportions of first births are higher, at 30 and 21%, respectively. These figures are in contrast to the national average of 13%. Increasing proportion of first births is a global trend as countries transition from high to low fertility. Studies show the relative impact of contraceptive use in reducing child mortality arising from high parity decreases as countries transition to low fertility, partly because of the increase in the proportion of high-risk first births (2, 17).

Among the single risk factors, births to women younger than 18 years and short birth intervals were associated with higher odds of under-five mortality, which is consistent with previous studies in Ethiopia (18, 19) and elsewhere (17, 20, 21).

The analysis also revealed that the trend in method mix in regions with relatively low modern contraceptive use (e.g. the Somali region) has been characterised by an increase in the use of pills [which have a higher rate of discontinuation (6)], a decrease in the use of injectables, and almost non-existent use of long-term methods such as implants. Hence, family planning programmes need to prioritise provision of wider method choices, including those with better effectiveness and convenience.

The multivariate analysis indicated that women in the Somali, Afar, and Benishangul-Gumuz regions had significantly higher likelihood of having avoidable risk births compared to the reference region (Addis Ababa) regardless of residence, education, religion, wealth, or discussion about family planning in health facilities. Being a rural resident and having no education were significantly associated with higher likelihood of avoidable birth risk, which can be partly explained by the fertility preferences of families of similar background (22). For example, the mean ideal number of children for all women in the Somali and Afar regions was 9.7 and 7.4, respectively, in 2011, compared to the national average of 4.3 (6). These data indicate the need to target context-specific beliefs and traditions around fertility preference, as achieving the goal of meeting current demand may not result in significant fertility decline in the foreseeable future.

There are a few limitations to this study. First, fertility is affected by multiple factors including fertility preference and intention, which may not be affected directly by a mere improvement in access to contraceptives. Second, the cross-sectional nature of the surveys precludes making temporal associations between contraceptive use and birth risk distributions, as both are measured at the same time. Finally, although we have attempted to account for variations in important individual-level factors, we must be cautious in making inferences at the regional level as there is substantial diversity in socio-cultural practices and beliefs within regions.

Nevertheless, the consistency of the relationship between the levels of contraceptive use, fertility behaviour of women, and the regional distribution of births with demographic risk factors indicate the possibility of cofounding as an alternative explanation is unlikely.

## Conclusions

The analyses revealed that there was a significant negative correlation between the level of modern contraceptive use among married women and TFR across the different regions of Ethiopia between 2000 and 2011, suggesting that the TFR declined on average by one child per woman for every increase in the mCPR of about 13 percentage points.

A majority of births in Ethiopia (62%) fell into one of the risk categories (excluding first births). There was substantial variation in the percentage of births with avoidable risk factors, however, from 55% in Gambela to 72% in the Somali region and 70% in Afar. The analysis indicated that the Somali region also has a slightly different pattern of method mix in favour of short-acting methods (pills), which could limit impact.

The trend analysis showed there was a significant drop in the proportion of births to women older than 34 years between 2000 and 2011 at the national level, while there was a significant decrease in the percentage of births with short birth interval only in Addis Ababa. There was no significant decline in births to women younger than 18 years between 2000 and 2011, so this remains an important risk factor, particularly in regions such as Amhara and Gambela where there are high rates of child marriage.

Living in the Somali and Afar regions is significantly associated with having higher odds of avoidable birth risk after controlling for observed covariates including wealth status, residence, education, religion, and discussion about family planning in health facilities.

Although Ethiopia has made significant progress in improving modern contraceptive uptake and decreasing high fertility, substantial regional variations persist, pointing the need to refocus family planning programmes to promote more equitable access to a broader choice of effective contraceptive methods throughout the country. Effort should also be made to prevent child marriage, which is often a precursor for teenage pregnancy and high-risk births.

## Supplementary Material

Trends in contraceptive use and distribution of births with demographic risk factors in Ethiopia: a sub-national analysis Click here for additional data file.

## References

[1] Cleland J, Conde-Agudelo A, Peterson H, Ross J, Tsui A (2012). Contraception and health. 2012. Lancet.

[2] Stover JR (2013). Changes in the distribution of high-risk births associated with changes in contraceptive prevalence. BMC Public Health.

[3] Kozuki N, Lee AC, Silveira MF, Sania A, Vogel JP, Adair L (2013). The associations of parity and maternal age with small-for-gestational-age, preterm, and neonatal and infant mortality: a meta-analysis. BMC Public Health.

[4] Lisonkova S, Janssen PA, Sheps SB, Lee SK, Dahlgren L (2010). The effect of maternal age on adverse birth outcomes: does parity matter?. J Obstet Gynaecol Can.

[5] The Alan Guttmacher Institute (2002). Family planning can reduce high infant mortality levels. Issues in Brief 2002 Series, No. 2.

[6] CSA, MEASURE DHS ICF Macro (2011). Ethiopia Demographic and Health Survey 2011.

[7] United Nations, Department of Economic and Social Affairs, Population Division (2014). World population prospects: the 2012 revision, methodology of the United Nations Population Estimates and Projections. ESA/P/WP.235.

[8] Ashenafi A, Karim AM, Ameha A, Erbo A, Getachew N, Betemariam W (2014). Effect of the health extension program and other accessibility factors on care-seeking behaviors for common childhood illnesses in rural Ethiopia. Ethiop Med J.

[9] Karim AM, Admassu K, Schellenberg J, Alemu H, Getachew N, Ameha A (2013). Effect of Ethiopia's health extension program on maternal and newborn health care practices in 101 rural districts: a dose-response study. PLoS One.

[10] Accorsi S, Bilal NK, Farese P, Racalbuto V (2010). Countdown to 2015: comparing progress towards the achievement of the health millennium development goals in Ethiopia and other sub-Saharan African countries. Trans Roy Soc Trop Med Hyg.

[11] CSA, ORC Macro (2000). Ethiopia Demographic and Health Survey 2000.

[12] CSA, ORC Macro (2005). Ethiopia Demographic and Health Survey 2005.

[13] Hogan DP, Biratu B (2004). Social identity and community effects on contraceptive use and intentions in southern Ethiopia. Stud Fam Plann.

[14] Tiruneh FN, Chuang KY, Ntenda PA, Chuang YC (2015). Factors associated with contraceptive use and intention to use contraceptives among married women in Ethiopia. Women Health.

[15] Lakew Y, Reda A, Tamene H, Benedict S, Deribe K (2013). Geographical variation and factors influencing modern contraceptive use among married women in Ethiopia: evidence from a national population based survey. Reprod Health.

[16] Boyden J, Pankhurst A, Tafere Y (2012). Child protection and harmful traditional practices: female early marriage and genital modification in Ethiopia. Dev Pract.

[17] Saha UR, van Soest A (2013). Contraceptive use, birth spacing, and child survival in Matlab, Bangladesh. Stud Fam Plann.

[18] Lindstrom DP, Berhanu B (2000). The effects of breastfeeding and birth spacing on infant and early childhood mortality in Ethiopia. Soc Biol.

[19] Susuman AS (2012). Child mortality rate in Ethiopia. Iran J Public Health.

[20] Raj A, McDougal L, Rusch ML (2014). Effects of young maternal age and short interpregnancy interval on infant mortality in South Asia. Int J Gynaecol Obstet.

[21] Houle B, Stein A, Kahn K, Madhavan S, Collinson M, Tollman SM (2013). Household context and child mortality in rural South Africa: the effects of birth spacing, shared mortality, household composition and socio-economic status. Int J Epidemiol.

[22] Bongaarts J (2011). Can family planning programs reduce high desired family size in sub-Saharan Africa?. Int Perspect Sex Reprod Health.

# Sub article: Contraceptive use and distribution of high-risk births in Nigeria: a sub-national analysis

**DOI:** 10.3402/gha.v8.29745

**Published:** 2015-11-09

**Authors:** Akanni Akinyemi, Sunday Adedini, Sennen Hounton, Ambrose Akinlo, Olanike Adedeji, Osarenti Adonri, Howard Friedman, Solomon Shiferaw, Abdoulaye Maïga, Agbessi Amouzou, Aluisio J. D. Barros

**Affiliations:** 1 Demography and Social Statistics Department, Obafemi Awolowo University, Ile Ife, Nigeria; 2 Demography and Population Studies Programme, University of Witwatersrand, Johannesburg, South Africa; 3 United Nations Population Fund, New York, NY, USA; 4 School of Research and Postgraduate Studies, Northwest University, Mafikeng, South Africa; 5 United Nations Population Fund, Abuja, Nigeria; 6 School of Public Health, Addis Ababa University, Addis Ababa, Ethiopia; 7 Institut Supérieur des Sciences de la Population, Ouagadougou University, Ouagadougou, Burkina Faso; 8 UNICEF, New York, NY, USA; 9 International Center for Equity in Health, Federal University of Pelotas, Capão do Leão, Brazil

**Keywords:** sub-national, estimates, contraceptive use, high-risk births, Nigeria

## Abstract

**Background:**

Family planning expansion has been identified as an impetus to harnessing Nigeria's demographic dividend. However, there is a need for data to address pockets of inequality and to better understand cultural and social factors affecting contraceptive use and health benefits. This paper contributes to addressing these needs by providing evidence on the trends and sub-national patterns of modern contraceptive prevalence in Nigeria and the association between contraceptive use and high-risk births in Nigeria.

**Design:**

The study utilised women's data from the last three Demographic and Health Surveys (2003, 2008, and 2013) in Nigeria. The analysis involved descriptive, bivariate, and multivariate analyses. The multivariate analyses were performed to examine the relationship between high-risk births and contraceptive use. Associations were examined using Poisson regression.

**Results:**

Findings showed that respondents in avoidable high-risk birth categories were less likely to use contraceptives compared to those at no risk [rate ratio 0.82, confidence interval: 0.76–0.89, *p*<0.001]. Education and wealth index consistently predicted significant differences in contraceptive use across the models.

**Conclusions:**

The results of this study suggest that women in the high-risk birth categories were significantly less likely to use a modern method of contraception relative to those categorised as having no risk. However, there are huge sub-national variations at regional and state levels in contraceptive prevalence and subsequent high-risk births. These results further strengthen evidence-based justification for increased investments in family planning programmes at the state and regional levels, particularly regions and states with high unmet needs for family planning.

**Paper context**

Using the last three Nigerian Demographic and Health Survey datasets, we examined the trends and sub-national patterns of modern contraceptive prevalence, as well as associations between contraceptive use and high-risk births in Nigeria. Findings showed that women in high-risk birth categories had lower uptake of modern contraceptive methods compared to those who were not in any high-risk birth category. The study further established huge sub-national variations in contraceptive prevalence and high-risk births, thus underscoring the need for increased investments in family planning programmes at the state and regional levels, particularly in areas with high unmet needs for family planning.

Nigeria is ranked among the 10 fastest growing populations in the world, is the most populous country in Africa, and the eighth most populous country in the world. The population is estimated to have grown from 56 million in 1970 to over 176 million in 2014 and is projected to be 184 million in 2015 with a growth rate of 3.2% (1). The total fertility rate (TFR) has been persistently high, from 5.7 in 2003 and 2008 to 5.5 in 2013, with regional estimates ranging from 4.3 in the South South region to 6.7 in the North West region. According to the 2013 Nigeria Demographic Health Survey (DHS), about 8.3% of births were unwanted and the adolescent birth rate was estimated at 122 per 1,000 women aged 15–19 years (only half the rate of women 25–29 years), with unmet need for contraception currently estimated at almost 50% among married women (2). These present significant challenges to harnessing the demographic dividend and creating sustainable development.

Although there has been a small increase in modern contraceptive use, from 8.9% in 2003 to 10.5% in 2008 and 11.1% in 2013 at the national level, the rate is still very low compared to the level of modern contraceptive prevalence in many other developing nations, which can be as high as 70% (3). Contraceptive use, particularly modern contraceptives, remains the main proximate determinant of fertility. According to the 2013 DHS, overall contraceptive prevalence among married women in Nigeria was estimated at 15.1%. However, there were wide variations at the sub-national level. Previous studies and the DHS documented reasons for non-use of contraceptive methods (2, 4–6), which included demographic and socioeconomic factors, fertility-related factors, opposition by partner, lack of knowledge, and family planning method–related reasons (2, 4–6). The need to improve access to and voluntary use of contraceptive methods is considered as a very important priority, particularly in a bid to harness the potential demographic dividends and improve the quality of life of individuals and the well-being of families.

Research has shown the benefit of expanding family planning services for improving maternal and child health, as well as increasing the well-being of individuals, families, and communities (7–12). Family planning promotion has been identified as a very important factor in addressing high-risk births and three major streams of preventable child and maternal deaths and in creating an AIDS-free generation (13). Research has shown that there is an inverse relationship between contraceptive use and high-risk births and that high-risk births are directly related to maternal and child mortality rates (8, 14–17). The estimates of high-risk births in Nigeria according to the 2013 DHS showed that two-fifths of all births to women and 32% of births to currently married women are high-risk births. About 23% of births to all women and 48% of all births to currently married women are categorised as multiple-risk births (2).

However, for a country like Nigeria with heterogeneous configurations, there is a need for a broader understanding of family planning and reproductive health issues, taking into account particular sectorial and sub-national differences. Even within one state, qualitative evidence confirmed variations in family planning adoption across local districts and communities in Nigeria (6). In addition, there is a need for evidence-based policy by demonstrating, at the state level, potential gains to maternal and child health associated with increasing contraceptive use. There are wide regional- and state-level variations in family planning and reproductive health indicators in Nigeria; providing a trend analysis at regional and state levels of the nexus between family planning and distribution of high-risk births may provide information for family planning intervention programmes that will respond to these diverse contexts.

This paper assesses the linkage between contraceptive prevalence, fertility levels and trends, and distribution of high-risk births in Nigeria at the sub-national level and uses the results to formulate recommendations for policy and programming. Sub-national divisions in Nigeria by region and state are shown in Fig. 1.

**Fig. 1:** Map of Nigeria, showing regions and states.

## Methods and Data

We utilised datasets from the last three DHSs in Nigeria (2003, 2008, and 2013) (2, 4, 18). Each of these surveys employed a cross-sectional design to collect nationally representative data from women of reproductive age (15–49 years). The indicators used include the following: high-risk births, modern contraceptive prevalence rate (mCPR), TFR, infant mortality rate, birth interval, and mother's age. Apart from the descriptive analysis, bivariate and multivariate analyses were performed to examine the influence of high-risk births on the outcome measure (contraceptive use). At the multivariate level of analysis, using only the 2013 DHS, Poisson regression was used to model the relationship between modern contraceptive use and high-risk births, controlling for education, wealth index, and region of residence. Wealth index is a proxy measure for standard of living because information on incomes is subjective and unreliable. Principal component analysis was used to generate wealth index from information based on each household's ownership of consumer goods, dwelling characteristics, and other household characteristics. Multivariate results were presented as rate ratios and respective 95% confidence intervals (CIs). Our outcome variable (modern contraception, meaning current use of at least one modern method of contraception at the time of the survey by married women or those in a union, aged 15–49 years) is a dichotomous variable. Nonetheless, preference was given to Poisson regression over binary logistic regression for this analysis, as many studies have argued and shown that Poisson regression is a better alternative for the analysis of cross-sectional studies with binary outcomes than logistic regression (19–21).

The main predictor variable was high-risk births. The births were categorised as 1) no risk, 2) unavoidable high-risk, and 3) avoidable high-risk. Births in the category of ‘no risk’ were second or third births to mothers between the ages of 18–34 years and with a preceding birth interval not less than 24 months, strictly representing lowest possible risk. ‘Unavoidable high-risk’ births were first births to mothers between the ages of 18–34 years (2). ‘Avoidable high-risk’ births were those to women aged 17 or younger, to women aged 35 or older, with a preceding birth interval of less than 24 months, and/or parity of four or higher (2). The high-risk birth variable was generated from the birth recode dataset to ensure that all births to interviewed women in the last 5 years were captured in the computation of high-risk births. This dataset was then merged with the women's individual recode dataset, since women are the main unit of analysis. The merged dataset thus excludes women who had not given birth in the last 5 years preceding the survey. Other explanatory variables selected include education, household wealth status, and region of residence. The main outcome variable was the use of modern contraception; we adopted the DHS categorisation of modern methods. Contraceptive use was categorised as 1) currently using modern method or 2) not currently using modern method. These variables have been reported as important factors influencing child survival (22, 23).

All analyses were performed using Stata 13 (24), taking into account the complex design of the DHS surveys.

## Results

### Fertility trends and dynamics

The national estimate of TFR showed a minimal decline from 5.7 in the periods 2003 and 2008 to 5.5 in 2013. Across the six regions as presented in Fig. 2, there are noticeable variations in trends within this period. Although the rates across the years remained highest in the North East and North West regions, there was a slight decline in the rates in the two regions between 2008 and 2013, from 7.2 to 6.3 in the North East region and 7.3 to 6.7 in the North West region. Except for the North Central region, there was a slight increase in the rates across the regions between 2003 and 2008. In addition, with the exception of only the South West region, there was a general decline between the 2008 and 2013 estimates across the region.

**Fig. 2:** Fertility trends, national and regional estimates.

However, within the regions, the states exhibited wide variations. For instance, in the North East region, comprised of six states, there were wide variations in the TFR as well as the trend over time. Three states showed a decline in TFR between 2003 and 2013 while two of the states showed an increase. In Bauchi State, for instance, the TFR increased from 7.2 in 2003 to 8.1 in 2013, whereas in Borno State, there was a decline from 7.0 in 2003 to 4.7 in 2013. In 2003, the range of TFR across the states was 7.8 in Yobe compared with 5.8 in Adamawa; it was 8.1 in Bauchi and 5.9 in Taraba in 2008; and 8.1 in Bauchi and 4.7 in Borno in the year 2013. Figure 3 presents the details.

**Fig. 3:** Fertility trends across the states in North East Nigeria, 2003–2013.

The age-specific fertility rates (ASFRs) presented in Fig. 4 show that across the three surveys, the ASFR was about 125 births per thousand young women below 20 years, while the number of births per thousand women aged 40–44 and 45–49 increased from 50 in year 2003 to 60 in 2008 and soared to about 80 in 2013. The ASFRs among women aged 20–24 and 45–49 have increased over the 10-year period and this rise portends serious consequences for the profile of high-risk births in Nigeria.

**Fig. 4:** Trends in age-specific fertility rate.

The age at which women begin childbearing may influence other fertility-related variables. Nationally, the mean age of first birth across the surveys was 18 years. However, this varies from 17 years in the North West to 22 years in the South East and South West regions. The TFR for women who started childbearing early (aged 18 years or below) is presented in Fig. 5. In the North West and North East regions, women who had their first birth before 18 years had a TFR of between 7 and 8 children, compared with 5.3 in the South West and 6.8 in the South East region. Figure 5 shows the relationship between TFR and mCPR across the regions; the distribution shows an inverse relationship between TFR and mCPR. Regions with considerably lower mCPR have very high fertility levels, ranging from 6.2 to 7.5 as shown in Fig. 6.

**Fig. 5:** Sub-national trends in total fertility rate for women who had their first birth before 18 years.

**Fig. 6:** Relationship between total fertility rate and modern contraceptive prevalence rate in Nigeria.

### Trends and regional distribution of high-risk births by fertility and contraception use

The distribution of births by risk factors varies considerably across the regions and by the level of TFR in each region. Figure 7 shows the distribution of births by risk factors and regional estimates of TFR. The proportion of births associated with avoidable risk is higher in the North West (72%) and North East (69%) regions compared with the South South (57%) and South West (45%) regions. The proportion of avoidable risk for all births is higher in regions with relatively higher levels of TFR (North West and North East) compared with the two regions with relatively lower levels of TFR (South West and South South). For the two regions with the highest level of TFR (North West and North East), more than 12% of births were to women less than 18 years, whereas only about 4% of births in the South West region and 7% of births in the South South region were among young women 18 years or less. The two regions (North West and North East) with the highest level of TFR have 15% of multiple risk factors (closely spaced birth interval, high birth order, and age of mothers) compared with 8% in South South Nigeria and 5% in South West Nigeria.

**Fig. 7:** Distribution of high-risk births by region, Nigeria 2013.

The proportion of women with high birth order was higher in North West and North East Nigeria, which constituted over 20% of all births in those regions, compared with less than 15% of all births in the South West and South South regions. South East Nigeria, however, had the highest proportion of closely spaced births, which constituted almost one-tenth of all births in the region, compared with about 6% of all births in North West Nigeria.

Further analysis to examine the patterns and trends of the distribution of births by risk factors in two regions (South South, with the lowest TFR, and North West, with the highest TFR) was also done (analysis not shown). In the South South region, the general pattern of the distribution of high-risk births shows that between 15 and 25% of all births across the states in three surveys are high parity. Bayelsa State, with the highest TFR in the year 2003, had the highest proportion of avoidable high-risk births, estimated at 65% in 2003, 63% in 2008, and 60% in 2013. However, there was no regular pattern in distribution of high-risk births and fertility levels in the states across the three surveys. In North West Nigeria, however, a major spotlight is on Kaduna State with a sharp decline in TFR from 7.3 in 2003 to 4.1 in 2013 and a corresponding decline in the proportion of high-risk births considered avoidable, which also dropped from 67% in 2003 to 60% in 2013. In contrast, Sokoto State experienced an upward trend in TFR from 5.2 in 2003 to 7.0 in 2013. The proportion of high-risk births categorised as avoidable also increased from 69% in 2003 to 74% in 2013. Generally, across the states in North West Nigeria, high-parity births constituted between 20 and 30% of all high-risk births, except for Sokoto in 2008. In addition, about 15–20% of all births across the states, except Sokoto in 2008, were among young women less than 18 years.

Figure 8 presents the distribution of high-risk births by modern contraceptive prevalence for all the surveys across regions and states. The graph shows the spread of the dots that reflects an inverse relationship between mCPR and the distribution of high-risk births. The graph shows that the proportion of births in high-risk categories is relatively higher in regions/states with lower levels of modern contraceptive prevalence.

**Fig. 8:** Distribution of proportion of high-risk birth by modern contraceptive prevalence, for regions (blue) and states (orange), 2003–2013.

### Multivariate analysis

Using Poisson regression, the relative risks (adjusted and unadjusted) of using a modern method of contraception are presented in Table 1. The results show that relative to women who were not in any high-risk birth category, the odds of using a modern method of contraception were significantly higher for women whose last births were unavoidable high-risk births (rate ratio 0.82, CI: 0.76–0.89, *p*<0.001). Adjusting for education and wealth index in Model 1, as shown in Table 1, results indicated that women in avoidable risk categories had a slightly higher rate of modern contraceptive use (rate ratio: 1.13, CI: 1.05–1.22, *p*<0.01) compared to those at no risk. In addition, rates of contraceptive use increased with higher levels of education and wealth status. For instance, results (Model 1) showed that women from the richest households (rate ratio 8.43, CI: 6.52–10.90, *p*<0.001) and women who had higher education (rate ratio 6.02, CI: 5.09–7.13, *p*<0.001) were more likely to use contraception than those in the reference categories. Adjusting for region of residence (Model 2) did not considerably alter these results. The relationship between region of residence and contraceptive use was significant, as results (Model 2) revealed significantly lower rates of contraceptive use in all of the other five regions compared to the South West region, the reference category.

**Table 1:** Poisson regression analysis showing rate ratios for modern contraceptive use in relation to high-risk births (2013 NDHS)

Characteristics	Unadjusted model	Adjusted model (Model 1)	Adjusted model (Model 2)
High-risk birth			
No risk	1	1	1
Unavoidable high risk	1.10 (0.99–1.23)	0.81 (0.73–0.90)***	0.81 (0.73–0.99)***
Avoidable high risk	0.82 (0.76–0.89)***	1.13 (1.05–1.22)**	1.20 (1.11–1.29)***
Wealth quintiles			
Poorest	1	1	1
Poorer	3.81 (2.96–4.90)***	2.59 (2.00–3.34)***	2.17 (1.68–2.81)***
Middle	9.19 (7.23–11.68)***	4.25 (3.30–5.48)***	3.06 (2.37–3.95)***
Richer	15.18 (12.00–19.20)***	5.59 (4.33–7.22)***	3.86 (2.99–5.00)***
Richest	27.7 (22.00–34.91)***	8.43 (6.52–10.90)***	5.54 (4.27–7.18)***
Education			
None	1	1	1
Primary	6.67 (5.83–7.64)***	4.10 (3.54–4.75)***	2.98 (2.55–3.49)***
Secondary	10.87 (9.58–12.33)***	5.01 (4.31–5.82)***	3.61 (3.08–4.24)***
Higher	16.77 (14.60–19.26)***	6.02 (5.09–7.13)***	4.25 (3.56–5.08)***
Region			
South West			1
North Central			0.90 (0.83–0.99)*
North East			0.37 (0.32–0.43)***
North West			0.33 (0.28–0.38)***
South East			0.67 (0.60–0.75)***
South South			0.82 (0.75–0.89)***

*** Significant at 0.05;

** significant at 0.01;

* significant at 0.001.

## Discussion

Expanding family planning programmes has been identified as a major prerequisite in order to harness the benefits of the demographic dividend in Nigeria (25–27). Part of realising this dividend requires a significant reduction in fertility as well as unrestricted access to voluntary and safe contraception through an effective family planning programme. Understanding the needs of individuals as well as sub-national variations in family planning promotion in Nigeria has been identified as a major gap in the low uptake of family planning.

In a bid to connect the present analyses with sub-national information at the state level, a review of information showed that the variations presented were confirmed and supported by evidence across the states. For instance, in the South South and South East regions, there are states whose fertility and family planning indicators are similar to those in the North West region, and this presents a wide dispersion from the regional estimates. Bayelsa and Ebonyi states are two examples of states in South South and South East Nigeria where family planning and maternal indices are relatively poor and similar to those in northern Nigeria. The geographic terrain of Bayelsa State, for instance, makes family planning and maternal health interventions challenging because about 90% of the communities are located in hard-to-reach areas in the riverine, core waterlogged Niger Delta. This is compounded by the religious beliefs of the people and general apathy towards modern family planning methods. In addition, the differentials in fertility, mCPR, and high-risk births are more pronounced in regions like the South East and South South. As a result of this, much is required in terms of investments in family planning programmes in such states as Bayelsa, Ebonyi, and others in Nigeria where contraceptive prevalence is low and high-risk births, as well as childhood and maternal mortality, remain high.

Further, Kaduna State in the North Central region of Nigeria showed family planning and maternal health indicators levels similar to those in South West Nigeria – the region with, relatively, the best reproductive health indicators in Nigeria. We also noted (analysis not shown) that Kaduna State, over the years, experienced a drastic drop in fertility rates. This decrease is perhaps partly due to the intensive efforts by the State Ministry of Health in collaboration with the National Urban Reproductive Health Initiative and United Nations Population Fund, which adopted an evidence-based approach to programming interventions.

The evidence of improvement of family planning and maternal health indicators in Kaduna State further illustrates the importance of state interventions in programmes and policies targeted towards expanding family planning outreach. This process requires a significant investment in positive directions including those targeted towards women's empowerment through education and direct investment in healthcare. This could be a good approach for more state investments in health and education.

The TFR of 5.5 at the national level and of over 7 in some regions is unacceptably high and thus may hinder reaping the gains of the demographic dividend. It is apparent that improving provision of family planning supplies and services, and the expansion of education and job opportunities for girls and women (25), have direct potential for improving quality of life by reducing the burden of high-risk births. The policy issues from this analysis further reiterate that expanding family planning is important in improving maternal and child health. There is a need to improve funding by state governments and stakeholders in expanding the reach of family planning services.

## Conclusions

As established in this study, expanding family planning services will undoubtedly lead to reduction in the burden of high-risk births in the various regions and states of Nigeria. Currently, there are huge state and regional differentials in the supply and demand for modern contraception. In addition, fertility remains significantly high and mCPR remains considerably low in many states across the country. Worse still, high-risk births remain very high in many states and regions. Hence, the sub-national analyses presented in this paper suggest that the demographic dividend possibility remains a mirage in many areas and regions of the country. There is therefore the need for sub-national investment and policies to improve family planning services, taking into account regional- and state-level particularities. Such policies and programmes need to recognise socio-cultural variations in the country.

## References

[1] United Nations (2012). World population prospects: the 2012 revision.

[2] National Population Commission (NPC) [Nigeria] and ICF International (2014). Nigeria demographic and health survey 2013.

[3] (2015). Family Planning and Reproductive Health Indicators Database.

[4] National Population Commission (NPC) [Nigeria] and ICF Macro (2009). Nigeria demographic and health survey 2008.

[5] Akinyemi AI, Aransiola JO, Banjo O, Bamiwuye O, Fadeyibi O, Adewuyi A (2010). Influence of independent and proximate variables on condom use in selected states in Nigeria. Afr J Reprod Health.

[6] Aransiola JO, Akinyemi AI, Fatusi AO (2014). Women's perceptions and reflections of male partners and couple dynamics in family planning adoption in selected urban slums in Nigeria: a qualitative exploration. BMC Public Health.

[7] Cleland J, Bernstein S, Ezeh A, Faundes A, Glasier A, Innis J (2006). Family planning: the unfinished agenda. Lancet.

[8] Cleland J, Conde-Agudelo A, Peterson H, Ross J, Tsui A (2012). Contraception and health. Lancet.

[9] Ahmed S, Li Q, Liu L, Tsui AO (2012). Maternal deaths averted by contraceptive use: an analysis of 172 countries. Lancet Global Health.

[10] Rutstein S (2005). Effects of preceding birth interval on neonatal, infant and under-five years mortality and nutritional status in developing countries: evidence from the Demographic and Health Surveys. Int J Gynaecol Obstet.

[11] Glasier A, Gulmezoglu AM, Schmid GP, Moreno CG, Van Look PF (2006). Sexual and reproductive health: a matter of life and death. Lancet.

[12] UNFPA (2008). Family planning and the environment: stabilizing population would help sustain the planet.

[13] United Nations (2013). A new global partnership: eradicate poverty and transform economies through sustainable development- the report of the High level Panel of Eminent Persons on the Post-2015 Development Agenda.

[14] Blank AK, Winfrey W, Ross J (2013). New finding for maternal mortality age patterns: aggregated results for 38 countries. PLoS One.

[15] Ross J (2005). How does contraceptive use affect infant and child mortality?.

[16] Stover J, Ross J (2009). How increased contraceptive use has reduced maternal mortality rates. Maternal Child Heath.

[17] Stover J, Ross J (2013). Changes in the distribution of high-risk births associated with changes in contraceptive prevalence. BMC Public Health.

[18] National Population Commission (NPC) [Nigeria] and ORC Macro (2004). Nigeria demographic and health survey 2003.

[19] Barros AJ, Hirakata VN (2003). Alternatives for logistic regression in cross-sectional studies: an empirical comparison of models that directly estimate the prevalence ratio. BMC Med Res Methodol.

[20] Greenland S (2004). Model-based estimation of relative risks and other epidemiologic measures in studies of common outcomes and in case-control studies. Am J Epidemiol.

[21] Zou G, Donner A (2013). Extension of the modified Poisson regression model to prospective studies with correlated binary data. Stat Methods Med Res.

[22] Adedini SA, Odimegwu C, Imasiku EN, Ononokpono DN (2015). Ethnic differentials in under-five mortality in Nigeria. Ethn Health.

[23] Antai D (2011). Regional inequalities in under-5 mortality in Nigeria: a population-based analysis of individual- and community-level determinants. Popul Health Metr.

[24] StataCorp LP (2013). Stata statistical software, release 13.

[25] Bloom D, Finlay J, Humair S, Mason A, Olaniyan O, Soyibo A (2010). Prospects for economic growth in Nigeria: a demographic perspective.

[26] Akinyemi A, Abanihe UI (2014). Demographic dynamics and development in Nigeria. Etude Popul Afr.

[27] Akinyemi A (2014). Opportunities and challenges for demographic dividend in Nigeria: bridging the gap between knowledge and use of family planning.

# Sub article: Patterns and trends of contraceptive use among sexually active adolescents in Burkina Faso, Ethiopia, and Nigeria: evidence from cross-sectional studies

**DOI:** 10.3402/gha.v8.29737

**Published:** 2015-11-09

**Authors:** Sennen Hounton, Aluisio J. D. Barros, Agbessi Amouzou, Solomon Shiferaw, Abdoulaye Maïga, Akanni Akinyemi, Howard Friedman, Desmond Koroma

**Affiliations:** 1 United Nations Population Fund, New York, NY, USA; 2 International Center for Equity in Health, Federal University of Pelotas, Capão do Leão, Brazil; 3 UNICEF, New York, NY, USA; 4 Department of Reproductive Health and Health Service Management, School of Public Health, Addis Ababa University, Addis Ababa, Ethiopia; 5 Centre for Demographic Research, Université Catholique de Louvain, Louvain-la-Neuve, Belgium; 6 Institut Supérieur des Sciences de la Population, Ouagadougou University, Ouagadougou, Burkina Faso; 7 Department of Demography and Social Statistics, Obafemi Awolowo University, Ile-Ife, Nigeria

**Keywords:** adolescents, childbearing, modern contraception, sub-Saharan Africa

## Abstract

**Background:**

The benefits of universal access to voluntary contraception have been widely documented in terms of maternal and newborn survival, women's empowerment, and human capital. Given population dynamics, the choices and opportunities adolescents have in terms of access to sexual and reproductive health information and services could significantly affect the burden of diseases and nations’ human capital.

**Objectives:**

The objectives of this paper are to assess the patterns and trends of modern contraception use among sexually active adolescents by socio-economic characteristics and by birth spacing and parity; to explore predictors of use of modern contraception in relation to the health system; and to discuss implications of the findings for family planning policy and programmes.

**Design:**

Data are from the last three Demographic and Health Surveys of Ethiopia, Burkina Faso, and Nigeria. The descriptive analysis focused on sexually active adolescents (15- to 19-year age group), used modern contraception as the dependent variable, and a series of contact points with the health system (antenatal care, institutional delivery, postnatal care, immunisation) as covariates. The multivariate analysis used the same covariates, adjusting for socio-economic variables.

**Results:**

There are two different groups of sexually active adolescents: those married or in a union with very low use of modern contraception and lower socio-economic status, and those unmarried, among whom nearly 50% are using modern contraception. Younger adolescents have lower modern contraceptive prevalence. There are significant inequality issues in modern contraception use by education, residence, and wealth quintile. However, while there was no significant progress in Burkina Faso and Nigeria, the data in Ethiopia point to a significant and systematic reduction of inequalities. The narrowing of the equity gap was most notable for childbearing adolescents with no education or living in rural areas. In the three countries, after adjusting for socio-economic variables, the strongest factors affecting modern contraception use among childbearing adolescents were marriage and child immunisation.

**Conclusions:**

Addressing child marriage and adopting effective policies and strategies to reach married adolescents are critical for improving empowerment and human capital of adolescent girls. The reduction of the equity gap in coverage in Ethiopia warrants further studies and documentation. The results suggest a missed opportunity for maternal and newborn and family planning integration.

**Paper context**

The choices and opportunities adolescents have in terms of access to sexual and reproductive health information and services could significantly impact nations’ human capital. We assessed patterns and trends of modern contraception use among sexually active adolescents in three countries (Burkina Faso, Ethiopia, and Nigeria) and explored health system predictors. The strongest predictors of contraception use among sexually active adolescents were marriage and child immunisation. There were pervasive inequality issues in contraception use by education, residence, and wealth quintile among sexually active adolescents; however, in Ethiopia, the data point to a significant and systematic reduction of inequalities, warranting further studies.

In the last few years, there has been a renewed effort from the global community to deliver contraception information and services to millions of women and adolescent girls who need them. The effects of family planning on maternal, perinatal, infant, and child health have been established through a systematic review of causal mechanisms (1). Population dynamics are central to sustainable development, and adolescents (defined here as 15–19 years old) are one of the fastest growing cohorts. Thus, understanding the patterns of contraception use among adolescents at the national and subnational levels could assist in designing strategies and programmes to better address coverage, quality, and equity issues at the country level (2–5). According to the 2014 State of the World Population Report (6), the world population includes the largest cohort of adolescents ever. The choices and opportunities that this adolescent group will have in terms of sexual and reproductive health information and services will determine, for example, the burden of adolescent pregnancies, unwanted pregnancies, abortions, HIV infections, and high school dropouts and may affect the ability of countries to harness the demographic dividend. Moreover, preventing adolescents from accessing comprehensive sexual and reproductive health information and services including family planning is a violation of their rights. Effectively addressing these burdens requires a better understanding of current patterns and trends of contraceptive use.

This paper examines data from Demographic and Health Surveys (DHS) in Burkina Faso, Ethiopia, and Nigeria. These countries were selected because of availability of survey data as part of the latest DHS rounds and because they provide a mix of characteristics, including geography, culture, religion, political decentralisation, population size, contraceptive prevalence, language, region, and economic indicators, representing some of the diversity in sub-Saharan Africa. In this paper, we assess sexually active adolescents in terms of their current use of modern contraception and trends in use. We also explore some subgroups, so that we can build a comprehensive picture of the dynamics of adolescent contraception use in these countries.

## Data and methods

We used data from publicly available national surveys (DHS) of Burkina Faso, Ethiopia, and Nigeria where information on adolescents’ sexual activity and contraception use were available. Burkina Faso is a landlocked country in West Africa with a low average contraception use and ranks 181 on the 2014 Human Development Index. Nigeria is also in West Africa, the most populous country in Africa with over 180 million inhabitants, a very low average contraceptive prevalence, and ranking 152 on the 2014 Human Development Index. Ethiopia is located in the Horn of Africa, with an estimated population of 80 million inhabitants, a federal government system similar to that in Nigeria, a recent increase in modern contraception use, and a ranking of 173 on the 2014 Human Development Index. Two surveys were included from Burkina Faso (2003 and 2010), three from Ethiopia (2000, 2005, and 2011), and three from Nigeria (2003, 2008, and 2013). The analyses focused on the use of modern methods of contraception among sexually active adolescents. Descriptive statistics on demographic factors (age and marriage), socio-economic factors (education, location, and wealth quintile), and birth risks (parity and birth spacing) were presented. Age was disaggregated into two groups (15–17 years and 18–19 years) to assess any differences for younger adolescents. Geographic differential effects were assessed at both urban and rural levels and also by states and regions. *Short spacing* was defined for women with at least two births as within a period of less than 24 months; parity was categorised as either zero, one, two, or more than two; and education was divided into no education, primary school completion, secondary level, or higher than secondary. Wealth quintiles were computed using household asset ownership and principal component analysis as described by Filmer and Pritchett (7).

We explored the determinants of modern contraception use among adolescents using the latest DHS in a multivariate analysis, with special interest in the effect of contact with the health system. The indicators we used as proxies for contact with the system included the number of antenatal care (ANC) visits, institutional delivery (yes or no), a postnatal care visit for the mother in the 2 months following delivery (yes or no), child immunisation (three doses of DTP3 used as a proxy and categorised as yes or no), visit to the household by a family planning health worker (yes or no), visit to a health facility by the mother in the past 12 months (yes or no), and whether or not information and counselling on family planning was received during a visit to a health facility. For this analysis, a logistic regression was used, adjusting for the potential confounding effect of socio-economic characteristics (education, residence, wealth quintiles). All the analyses were performed with Stata 13.0 statistical software (8), taking into account the design characteristics of the surveys. Ethical clearances were secured by the organisations that carried out the original surveys.

## Results

The cumulative sample sizes were 17,087 for Burkina Faso, 16,515 for Ethiopia, and 38,948 for Nigeria.

### Distribution of modern contraception use among sexually active adolescents over time, by marital status and age

Using the latest available DHS, most sexually active adolescents in the three countries studied were either married or in a union (henceforth referred to as *in union*): 88% (*N*=1,127), 96% (*N*=846), and 86% (*N*=2,480) in Burkina Faso, Ethiopia, and Nigeria, respectively. The prevalence of modern contraception use among sexually active adolescents was low in the three countries, the latest estimates being 11.2% for Burkina Faso, 24.1% for Ethiopia, and 7.8% for Nigeria (Fig. 1). There was a substantial difference in terms of modern contraception use between adolescents in union and those who were not in union (Fig. 2). The latest prevalence of use in the latter group was 7.8 times, 2.3 times, and 43.65 times higher than those in union in Burkina Faso, Ethiopia, and Nigeria, respectively. Overall, the proportion of modern contraception use among sexually active adolescents has remained almost unchanged (Fig. 1) in both Burkina Faso (at 11%, from 2003 to 2010) and in Nigeria (at around 8%, from 2008 to 2013). In contrast, in Ethiopia, the prevalence of use has been increasing sharply over the last decade, from 5 to 24%. Figure 2 shows that this increase has happened mostly among women in union (96% of sexually active adolescents). In Nigeria, we observed the opposite – a steep increase in contraceptive use among women not in union (14% of sexually active adolescents), with a decrease of use among those in union.

**Fig. 1:** Trends in modern contraceptive use (% mCPR) among sexually active adolescents in Burkina Faso, Ethiopia, and Nigeria. Source: Demographic and Health Surveys.

**Fig. 2:** Trends in modern contraception use (% mCPR) among sexually active adolescents by marital status in Burkina Faso, Ethiopia, and Nigeria. Source: Demographic and Health Surveys.

There was no statistically significant difference between the adolescents in the 15- to 17-year-old and the 18- to 19-year-old age groups in the use of modern methods of contraception at specific points in time (the last three iterations of the DHS).

### Disaggregation by socio-economic status

Table 1 presents the trends in modern contraceptive use among sexually active adolescents by education, place (rural or urban), and wealth quintiles in Burkina Faso, Ethiopia, and Nigeria. For Burkina Faso and Nigeria, there was a consistent disparity in the distribution of use of modern contraception among sexually active adolescents by education level, with the more educated adolescents more likely to be users compared to those adolescents who had not been to school. There was no statistically significant change in the prevalence of modern contraception use in Burkina Faso and Nigeria. This is in contrast to Ethiopia, where there has been a large and positive average annual rate of change over the years across all education levels. In all three countries, there was a huge gap in use between those with primary-level education and all levels above. The latest prevalence of use among adolescents with a secondary-level education or above was 2.4 times, 1.9 times, and 5.9 times higher in Burkina Faso, Ethiopia, and Nigeria, respectively, than those who had completed only primary-level education.

**Table 1:** Trends in modern contraception use among sexually active adolescents in Burkina Faso, Ethiopia, and Nigeria, by education, geographic location, and wealth quintile

	Burkina Faso			Ethiopia				Nigeria			
			
Variables	2003	2010	AAR*	2000	2005	2011	AAR*	2003	2008	2013	AAR*
Education											
None	7.4	5.5	−2.1	3.1	5.4	14.2	10.1	1.0	0.8	0.2	−12.6
Primary	19.9	18.0	−0.7	12.7	14.1	28.8	5.6	8.6	5.6	4.6	−4.5
Secondary or greater	52.1	43.6	−1.3	9.8	59.0	54.6	11.3	27.8	27.3	27.0	−0.2
Place											
Urban	38.4	35.3	−0.6	18.7	46.7	49.0	6.5	11.7	20.6	22.9	4.6
Rural	5.8	5.1	−0.9	3.4	6.6	20.9	11.9	6.6	6.1	4.0	−3.6
Wealth quintiles											
Lowest	4.1	2.9	−2.5	3.2	3.4	16.7	11.0	3.3	1.8	0.3	−19.0
Second	4.1	4.5	0.6	1.0	4.7	18.4	18.2	2.1	5.0	2.6	1.3
Middle	6.5	4.2	−3.2	1.0	3.2	19.9	19.1	4.4	8.5	10.4	5.8
Fourth	10.7	10.0	−0.5	0.8	11.9	24.3	21.0	16.5	18.7	19.5	1.2
Highest	34.2	36.7	0.5	20.9	31.0	52.3	6.2	20.9	36.1	39.2	4.3

AAR*: average annual percent rate of change between the latest and earliest DHS in the series considered for the analysis; none: no education; primary: primary completed; secondary or greater: above primary school.

Residence follows a pattern similar to education, with a greater gap between urban and rural areas. The latest prevalence of use among adolescents in urban areas was almost 7 times, 2.3 times, and 5.7 times greater than those who lived in rural areas in Burkina Faso, Ethiopia, and Nigeria, respectively. In addition, in Ethiopia, it is important to note an almost 2.6-fold and 6.1-fold increase in modern contraception use by adolescents from 2000 to 2011, in urban and rural areas, respectively. Moreover, the equity gap between urban and rural areas has been decreasing over time, and a similar pattern was observed to some extent in the differences in educational level and wealth quintile. Systematic analyses of the reasons for success in reduction in equity gaps will be important in order to understand the results and share lessons with other countries with prevailing inequities.

### Subnational variations in modern contraception use among sexually active adolescents in Burkina Faso (2010), Ethiopia (2011), and Nigeria (2013)

As reflected by the distribution of modern contraception use among adolescents in rural and urban areas, there were large differences in the proportions of sexually active adolescents using modern contraception in the three countries (Figs. 3–5). As reflected in the differentials between urban and rural areas, the highest prevalence of use of modern contraception was recorded in the regions hosting the two main cities in Burkina Faso (Centre and Hauts-Bassins); the Addis Ababa region in Ethiopia; and Osun, Enugu, and Lagos States in Nigeria.

**Fig. 3:** Mapping of prevalence of modern contraception use among sexually active adolescents by region, Burkina Faso. Source: 2010 Demographic and Health Survey.

**Fig. 4:** Mapping of prevalence of modern contraception use among sexually active adolescents by region, Ethiopia. Source: 2011 Demographic and Health Survey.

**Fig. 5:** Mapping of prevalence of modern contraception use among sexually active adolescents by state, Nigeria. Source: 2013 Demographic and Health Survey.

There were similarities across the three countries in the effect of region: the lowest percentages of use were recorded in predominantly Islamic regions and states (the Sahel, Nord, and Centre-Nord regions in Burkina Faso; the Afar and Somali regions in Ethiopia; and northern Nigeria). In Nigeria, it was striking to see almost no use of modern contraception among sexually active adolescent girls in Sokoto, Yobe, Zamfara, Kano, Katsina, Kebbi, Jigawa, Nasarawa, Borno, and Bauchi, which are all in northern Nigeria. Although there is homogeneity in northern Nigeria, significant differences between states could be observed within other regions. For example, although the average modern contraception use estimate among sexually active adolescents in the south-east in 2013 was 32.6%, Enugu State recorded 50.7% while Imo State recorded 13.2%. Similarly, although the use estimate among sexually active adolescents in the south-west in 2013 was 33.9%, Osun State recorded 65% (95% CI: 45–81%), while Oyo State recorded 15% (95% CI: 4.8–38%).

### Distribution of modern contraception use by parity and birth spacing

In the latest DHS, the proportions of sexually active adolescents without a child, with one child, and with two or more children was 53.2, 39.8, and 7% in Burkina Faso, 55.3, 33.6, and 11.1% in Ethiopia, and 54.8, 35, and 10.2% in Nigeria, respectively. The effect of parity on the pattern of modern contraception use among sexually active adolescents over the years was similar in Burkina Faso and Nigeria (Table 2). There was no significant trend in modern contraception use among sexually active adolescents with one child and two or more children as compared to those with no children. In Ethiopia, however, there was an increasing trend in modern contraception use over the years, irrespective of parity. Modern contraception use had increased among first-time young mothers from 12% in 2005 to 24% in 2011.

**Table 2:** Patterns of modern contraception use by parity and marital status among sexually active adolescents in Burkina Faso, Ethiopia, and Nigeria

Countries	Burkina Faso, 2010 DHS		Ethiopia, 2011 DHS		Nigeria, 2013 DHS	
			
Covariates	In union	Not in union	In union	Not in union	In union	Not in union
Parity	Prevalence	Prevalence	Prevalence	Prevalence	Prevalence	Prevalence
0	6.2	47.3	23.7	56	0.1	51.8
(95% CI)	3.3–11.2	37–57.8	17.4–31.6	32.8–76.8	0.0–0.4	45.3–58.3
1	5.7	65.3	24	72	2.2	33.1
(95% CI)	3.7–8.5	21.8–92.7	17.3–32.2	16.2–97.2	1.3–3.6	18.7–51.6
2 or more	9.0	0	15	0	1.9	43.3
(95% CI)	4.5–17.3	−	7.3–29.7	–	0.6–5.5	5.9–88.8

CI: confidence interval; DHS: Demographic and Health Survey. Missing values are due to very small sample sizes.

The relationship between modern contraception use and parity varied significantly by marital status. Using the latest DHS in each of the three countries, the prevalence of modern contraception use among sexually active adolescents with one child, who were not in union, was 65.3, 72.2, and 33.1% in Burkina Faso, Ethiopia, and Nigeria, respectively. These are 11.5 times, 3.0 times, and 15.3 times the prevalence for those in union in these countries, respectively.

Similarly, for adolescents with no child, prevalence of modern contraception use among adolescents not in union was 47.3, 56, and 41.3%, which was 7.7 times, 2.4 times, and 411 times the prevalence for those in union in Burkina Faso, Ethiopia, and Nigeria, respectively. These differences across countries are staggering and may point to a strong negative effect of marriage on the ability of adolescent girls to control their own fertility, irrespective of the level of modern contraceptive prevalence. It is also possible that, once married, adolescents want to conform to social norms and do not want to use contraceptives, although the data on unmet need for modern contraception among adolescents support the latter hypothesis less (6).

Regarding birth spacing, there was an inherent limitation for adolescents 15–19 years in the data sets for the analysis in terms of sample size, given that birth spacing can only be reliably explored for mothers with at least one child and a lag time of 24 months after each birth. For example, if an adolescent girl has a child at age 18, we do not have enough lag time in our sampling frame (limited to her 20th birthday) to observe whether or not she would have had short birth spacing or normal birth spacing as she moves out of the adolescent age group. For the purpose of this analysis, we decided to look mainly at any differences in the prevalence of modern contraception use among short birth spacing versus spacing of at least 24 months (normal spacing). There was no significant difference in the prevalence of modern contraception use by adolescent mothers by birth interval (spacing). The very small sample sizes after disaggregation did not allow for a comparative analysis of spacing by marital status.

### Effects of selected health services on modern contraception use among sexually active adolescents

We explored the effects on the use of modern contraception of ANC visits, institutional delivery, a postnatal care visit in the 2 months following delivery, child immunisation (DTP3), visit to the household by a family planning health worker, visit to a health facility by the mother in the past 12 months, and receipt of information and counselling on family planning during a visit to a health facility. We explored the variables individually and in a model, for all sexually active adolescents as well as for childbearing adolescents. The overall results underscore that, regardless of modern contraceptive prevalence rate, more contacts with health services are associated with increased modern contraception use.

More specifically, in Burkina Faso, the prevalence of modern contraception use among sexually active adolescents was 9.2% for four ANC visits, 7.9% for childbearing adolescents with postnatal care for the baby, 10.3% for adolescents whose babies completed DTP3, and 8.6% for adolescents who delivered in health facilities. These were approximatively 1.5 times, 13 times, and 7.3 times the prevalence for those with only one ANC visit, no postnatal care visit, no DTP3, and no institutional delivery, respectively. We did not observe a significant effect of household visit by a family planning health worker, visit to a health facility by the mother in the past 12 months, or receipt of information and counselling on family planning during a visit to a health facility.

In Ethiopia, there was a positive association of contraceptive use with all of the variables. The prevalence of modern contraception use among sexually active adolescents was 51.6% for four ANC visits, 48.5% for childbearing adolescents who used postnatal care services, 43.3% for adolescents whose babies completed DTP3, 22.1% for adolescents who delivered in health facilities, 37.6% for those who had a household visit by a family planning health worker, 37.4% for those who had visited a health facility in the past 12 months, and 37.5% for those who received information and counselling on family planning during a visit to a health facility. These values were approximately 3.6, 2.5, 2.5, 1.1, 1.6, 2, and 16.3 times the prevalence for those with only one ANC visit, no postnatal care visit, no DTP3, no institutional delivery, no visit by family planning health worker, no visit to a health facility in the past 12 months, and no counselling on family planning during a visit to a health facility, respectively.

In Nigeria, the pattern was very similar to that observed in Burkina Faso, with a positive association of use with four ANC visits, postnatal care for the baby, completion of DTP3, and institutional delivery but no effect of household visit by a family planning health worker, visit to a health facility by the mother in the past 12 months, or receipt of information and counselling on family planning during a visit to a health facility. Subsequently, we elected to assess variance in modern contraception use among sexually active adolescents that could be explained by opportunistic contacts with the health system, such as ANC, institutional delivery, and child immunisation, adjusting for socio-economic variables (education, residence, and wealth quintile) and marital status. The results are presented in Table 3.

**Table 3:** Univariate and multivariate logistic regression of the effect of health service contacts on modern contraception use among adolescents in Burkina Faso, Ethiopia, and Nigeria

Variables	Crude OR (95% CI)	Adjusted OR (95% CI)
Burkina Faso		
Antenatal care (4 visits)	1.6 (0.8–3.2)	1.1 (0.5–2.4)
Child immunisation^a^ (completion of DTP3)	8.0 (1.8–35.9)	8.9 (3.0–26.7)
Institutional delivery (yes)	8.5 (1.1–63.2)	2.9 (0.3–26.3)
Marital status (married)	0.07 (0.04–0.11)	0.2 (0.0–2.4)
Education		
Primary	3.8 (2.2–6.5)	0.9 (0.3–2.8)
Secondary or above	13.3 (7.9–22.5)	1.2 (0.3–3.4)
Residence (urban)^a^	10.2 (6.6–15.7)	3.7 (1.5–9.3)
Wealth quintiles		
Second	1.6 (0.6–4.1)	2.4 (0.3–20.6)
Middle	1.5 (0.5–3.9)	1.6 (0.2–15.2)
Fourth	3.7 (1.5–9.4)	3.4 (0.4–31.3)
Highest	19.4 (8.2–4.6)	6.7 (0.8–56.7)
Ethiopia		
Antenatal care		
1 visit	0.6 (0.2–1.4)	0.9 (0.3–2.4)
4 visits	3.7 (1.3–10.3)	2.8 (0.9–8.7)
Child immunisation^a^ (completion of DTP3)	3.7 (1.6–8.4)	3.5 (1.4–8.3)
Institutional delivery (yes)	2.1 (0.7–6.1)	4.9 (0.4–52.7)
Marital status (married)^a^	0.3 (0.1–0.6)	0.02 (0.00–0.74)
Education		
Primary^a^	2.5 (1.5–4.0)	3.1 (1.1–8.5)
Secondary or above	7.3 (2.8–19.2)	4.6 (0.4–52.7)
Residence (urban)	3.6 (1.8–7.2)	10.3 (0.6–164.7)
Wealth quintiles		
Second	1.1 (0.5–2.4)	0.5 (0.2–1.9)
Middle	1.2 (0.6–2.6)	0.5 (0.2–1.6)
Fourth	1.6 (0.8–11.6)	0.4 (0.1–2.0)
Highest	5.5 (2.6–11.6)	1.3 (0.2–11.8)
Nigeria		
Antenatal care		
1 Visit	0.5 (0.1–2.5)	1.4 (0.3–7.8)
4 Visits	4.3 (1.1–16.8)	2.4 (0.6–9.5)
Child immunisation (completion of DTP3)	8.6 (3.8–19.2)	1.9 (0.7–5.0)
Institutional delivery (yes)	3.5 (1.6–7.7)	0.7 (0.3–1.7)
Marital status (married)^a^	0.01 (0.00–0.02)	0.1 (0.03–0.40)
Education		
Primary	25.5 (5.5–128.8)	4.3 (0.7–27.8)
Secondary or above^a^	196.8 (45.5–851.5)	7.7 (1.5–40.5)
Residence (urban)	7.1 (5.1–9.9)	1.1 (0.4–2.7)
Wealth quintiles		
Second	9.2 (2.8–30.9)	2.4 (0.3–18.5)
Middle	40.5 (12.7–129.3)	2.5 (0.4–14.2)
Fourth	85.1 (26.8–270.3)	5.3 (0.9–31.5)
Highest	226.0 (70.1–728.5)	2.6 (0.3–22.4)

a Significant at the 5% level. Crude and adjusted odds ratio (OR) estimates with their 95% confidence interval (95% CI) of the effect of selected health services on modern contraception use among sexually active adolescents in Burkina Faso (2010 DHS), Ethiopia (2011 DHS), and Nigeria (2013 DHS). Crude OR calculated from a univariate logistic regression. Adjusted OR calculated from a multivariate logistic regression adjusting for other variables in the model.

In a simple, non-adjusted multivariate analysis, there were several variables that were associated with use of modern contraception among sexually active adolescents in all three countries. These included child immunisation, marital status, education (all levels), residence, and wealth quintile (least poor). After adjusting for socio-economic variables, there were fewer variables with a statistically significant association with modern contraceptive use among sexually active adolescents. These included child immunisation and residence in Burkina Faso; child immunisation, marital status, and education (primary level) in Ethiopia; and marital status and education (secondary level and above) in Nigeria.

## Discussion

### Demographic and socio-economic variations across countries

There were significant variations in the use of modern contraception by demographic and socio-economic characteristics in Burkina Faso, Ethiopia, and Nigeria, despite being in the same geographic region of sub-Saharan Africa. While in Burkina Faso and Nigeria there has been no significant progress over the last three surveys at the national level, the data from the 2011 DHS in Ethiopia indicate a sharp increase in the use of modern contraceptives among sexually active adolescents. It will be important to identify the policies and strategies being implemented in Ethiopia and share these findings with other countries. Regarding age, although the difference was not statistically significant, the use of modern contraception tended to be lower for younger adolescents. This finding is confirmed by a recent study in Kenya, Tanzania, and Uganda (9) where the authors found the relationship between adolescent motherhood and lack of contraception use was strongest among births within the youngest age group (<16 years old). For marital status, the main finding in our study, also confirmed by other authors (9, 10), is that adolescents in union constitute a vastly different group from adolescents not in union. One of our main questions was whether adolescent women in union would be more likely to use contraception after having their first child. The data in our study suggest that first-time, married young mothers are not using modern contraception. They are also more likely to be less educated, poorer, and to be in rural areas as compared to those that are not married. This situation increases their vulnerability for increased fertility and higher risks of mortality and fistula.

The data on socio-economic variables allow us to analyse equity issues. Across all three countries, there is a significant equity gap in modern contraception use. Adolescents who have a high school level education or above, who are in urban areas, and who are in the highest wealth quintiles use significantly more modern contraception as compared to their peers who have primary-level education, live in rural areas, or who belong to the lowest wealth quintiles. These findings are similar to the trends and patterns observed in sub-Saharan Africa or at the global level by other authors (11, 12). However, the countries differ in the direction and type of changes observed over time. In Nigeria, the equity gap has worsened over the years, with a reduction in the average annual rate in modern contraceptive prevalence of 12.6, 3.6, and 19% for childbearing adolescents with no education, living in rural areas, or belonging to the lowest wealth quintile, respectively. The lowest quintile is the only category within the wealth quintiles to have a huge increase in the equity gap as compared to the other quintiles. The data also suggest a similar pattern in Burkina Faso, where the worst increase in the equity gap affecting modern contraception use among childbearing adolescents was observed for the lowest (first), second, and third quintiles, as well as for the group with no education.

In contrast, data from Ethiopia revealed a significant and systematic reduction in the equity gap over the years in terms of education, geographic location, and wealth quintiles. The narrowing of the equity gap was notable for childbearing adolescents with no education or living in rural areas, with an average annual increase in the rate of modern contraception use of 10 and 12%, respectively. The narrowing of the equity gap was greatest for the second, third, and fourth quintiles, with an average of 20% annual increase in the rate of modern contraception use among all three groups combined. These results are worth noting, given that Ethiopia has been cited as one of the countries with the highest inequities in health services among many other sub-Saharan countries (13, 14). Ethiopia has made massive investments in primary healthcare and a national community health-worker programme (referred to as the *Health Extension Workers Programme*), with community workers on the government payroll who are trained, supervised, and equipped to provide modern contraception at the last mile, including implants (15). The data also suggest a need for further research to investigate if the same reduction in the equity gap is observed across other maternal and newborn interventions. Systematic analyses of the reasons for success in reducing inequity will be important to document and share with other countries with greater inequities.

### Subnational variations

With increasing decentralisation and empowerment of local governments at subnational levels (states, regions, and counties are increasingly setting priorities and budget allocations across development sectors), it is critical to provide disaggregated data for evidence-based policy making and programme design. The data show some similarities across the three countries, with the lowest modern contraception rates recorded among sexually active adolescents in the poorest regions and states and in rural areas. It is also interesting to note that across the three countries there is very low contraceptive prevalence in largely Islamic populations, although predominantly animist regions in Burkina Faso also had very low prevalence rates. It is not surprising that these states and regions with very low contraception use also have the highest prevalence of child marriage and lowest literacy rates. In fact, child marriage has shown to be associated with unintended pregnancy, low levels of contraceptive use, and limited use of maternal health services (16), which result in increased vulnerability for negative maternal outcomes.

These factors alone, however, do not account for all the variance in contraception use across states and regions. Even within the same geographic areas, we observed significant variations among neighbouring states, such as is the case in Nigeria for the states of Osun and Oyo in the south-west or between the states of Imo and Abia in the south-east. These differences point to possible differences in policy, strategies, and investment by local governments in women's, children's, and adolescents’ health, as well as in cultural and societal norms and values with regards to keeping adolescent girls in school, curtailing child marriage, and increasing access to modern contraception for all women of reproductive age.

### Determinants of modern contraceptive use among sexually active adolescents

Using the latest DHS data, the multivariate analysis of potential factors explaining the variance in modern contraception use among childbearing adolescents was very revealing and points to some commonalities, as well as missed opportunities, for integrating family planning services with maternal and newborn health across the three countries. There was a very strong and negative impact of marriage, even after adjusting for all other variables. The adjusted odds of a childbearing adolescent using modern contraception when she is not in union are 5 times, 50 times, and 10 times that of married adolescents for Burkina Faso, Ethiopia, and Nigeria, respectively. It is therefore critical to reach those childbearing adolescents in union in order to improve their maternal and newborn outcomes (16). Having at least a high school education, living in urban areas, and belonging to the highest wealth quintiles may just reflect the same group of adolescents who are better off as compared to others and who have more access to modern contraceptive information and services.

The pattern and strength of change in these factors also varies across countries after adjusting for socio-economic variables (education, residence, wealth quintiles). While in Burkina Faso the strongest associations with contraceptive use were observed for child immunisation and residence, in Ethiopia it was child immunisation, marital status, and education (primary school), and in Nigeria marital status and education (secondary school). Why ANC and institutional delivery did not significantly affect use of contraception after adjusting for socio-economic variables would be interesting to understand. A possibility is that these factors are influenced by residence in Burkina Faso, where health services are more accessible and available in urban areas; the same could be said for adolescents with primary-level education or above in Ethiopia and Nigeria, who may best represent those living in cities with more access to ANC and institutional delivery.

One can clearly see the importance of integration of maternal and newborn health services (17), particularly child immunisation, the strongest and most significant modifier of modern contraceptive use among childbearing adolescents. Are the childbearing adolescents more likely to become users of modern contraception when their children complete DTP3, or are the adolescents who ensure completion of their children's immunisation more likely to hear about contraception and services and adopt family planning?

Regardless, child immunisation appears to be a critical factor and thus a missed opportunity for improving the coverage of family planning among childbearing adolescents. The odds that a childbearing adolescent will use modern contraception when her newborn completes DTP3 (which corresponds roughly to a period of 3 months after birth) is 9 times, 3.5 times, and 2 times the odds in the absence of exposure to child immunisation in Burkina Faso, Ethiopia, and Nigeria, respectively. Finally, the results of the multivariate analysis, after adjusting for socio-economic and demographic factors, may hide the effects of other variables such as cultural norms. Policies and strategies to provide information and services for contraception, which are critical for the empowerment and rights of adolescent girls and women, need to build on the most contextually relevant drivers for use of modern contraception.

### Limitations

Some limitations of the study are related to the small sample sizes. Another limitation in the analysis of birth spacing is the short period available to observe birth spacing during adolescence, given the age restriction (15–19 years) and the definition of a normally spaced birth (24 months). These limitations often resulted in very wide confidence intervals, which limited statistical analysis and the conclusions that could be made.

## Conclusions

This paper highlights the importance of understanding subnational variations and differences in modern contraceptive use among sexually active adolescents in order to better address their needs. Although there are prevailing equity issues, the data from Ethiopia are an indication that, when proper policies and investments are made (e.g. the Health Extension Workers Programme and availability of modern contraceptives at the last mile) (15), it is possible to significantly reduce the equity gap in a short period of time. Marriage remains, by far, the major bottleneck for childbearing adolescents’ use of modern contraception. Improving modern contraception use among sexually active adolescents will require capitalising on missed opportunities for contact with the health system, particularly during child immunisation. Banning child marriage, ensuring comprehensive sexuality education and connecting childbearing adolescents with information and services during routine health services such as ANC, institutional delivery, and child immunisation are critical to meeting their needs and improving adolescent girls’ well-being, empowerment, and human capital.

## References

[1] Conde-Agudelo A, Rosas-Bermudez A, Castaño F, Norton MH (2012). Effects of birth spacing on maternal, perinatal, infant, and child health: a systematic review of causal mechanisms. Stud Fam Plan.

[2] Darroch JE, Singh S (2013). Trends in contraceptive need and use in developing countries in 2003, 2008, and 2012: an analysis of national surveys. Lancet.

[3] Bongaarts J (2014). How exceptional is the pattern of the fertility decline in sub-Saharan Africa?. UN Population Division, Expert paper No. 2013/4.

[4] Bongaarts J, Frank O, Lesthaeghe R (1984). The proximate determinants of fertility in sub-Saharan Africa. Popul Dev Rev.

[5] Cleland JG, Ndugwa RP, Zulu EM (2011). Family planning in sub-Saharan Africa: progress or stagnation?. Bull World Health Organ.

[6] UNFPA (2014). State of the World Population Report.

[7] Filmer D, Pritchett LH (2011). Estimating wealth effects without expenditure data – or tears: an application to educational enrolments in states of India. Demography.

[8] STATA 13.0 statistical software.

[9] Neal SE, Chandra-Mouli V, Chou D (2015). Adolescent first births in East Africa: disaggregating characteristics, trends and determinants. Reprod Health.

[10] Neal S, Matthews Z, Frost M, Fogstad H, Camacho AV, Laski L (2012). Childbearing in adolescents aged 12–15 in low resource countries: a neglected issue. New estimates from demographic and household surveys in 42 countries. Acta Obstet Gynecol Scand.

[11] Nove A, Matthews Z, Neal S, Camacho AV (2014). Maternal mortality in adolescents compared with women of other ages: evidence from 144 countries. Lancet Global Health.

[12] Chandra-Mouli V, Carraher D, Phillips S, Williamson N, Hainsworth G (2014). Contraception for adolescents in low and middle income countries: needs, barriers, and access. Reprod Health.

[13] Boerma JT, Bryce J, Kinfu Y, Axelson H, Victora CG, on behalf of Countdown Equity Analysis Group (2008). Mind the gap: equity and trends in coverage of maternal, newborn, and child health services in 54 Countdown countries. Lancet.

[14] Barros AJ, Ronsmans C, Axelson H, Loaiza E, Bertoldi AD, França GV (2012). Equity in maternal, newborn, and child health interventions in Countdown to 2015: a retrospective review of survey data from 54 countries. Lancet.

[15] Workie NW, Ramana GNV (2013). Ethiopia – the health extension program in Ethiopia. Universal Health Coverage (UNICO) studies series; no. 10.

[16] Godha D, Hotchkiss DR, Gage AJ (2013). Association between child marriage and reproductive health outcomes and service utilization: a multi-country study from South Asia. J Adolesc Health.

[17] Ricardo V, Foreit JR, Ottolenghi E (2006). Adding systematic screening to your program: a manual. FRONTIERS manual.

# Sub article: Patterns and trends of postpartum family planning in Ethiopia, Malawi, and Nigeria: evidence of missed opportunities for integration

**DOI:** 10.3402/gha.v8.29738

**Published:** 2015-11-09

**Authors:** Sennen Hounton, William Winfrey, Aluisio J. D. Barros, Ian Askew

**Affiliations:** 1 United Nations Population Fund, New York, NY, USA; 2 Avenir Health, Glastonbury, CT, USA; 3 International Center for Equity in Health, Federal University of Pelotas, Capão do Leão, Brazil; 4 Population Council, Nairobi, Kenya

**Keywords:** postpartum contraception, integration, maternal and newborn health services, sub-Saharan Africa

## Abstract

**Background:**

The first 12 months following childbirth are a period when a subsequent pregnancy holds the greatest risk for mother and baby, but also when there are numerous contacts with the healthcare system for postnatal care for mother and baby (immunisation, nutrition, etc.). The benefits and importance of postpartum family planning are well documented. They include a reduction in risk of miscarriage, as well as mitigation of (or protection against) low birth weight, neonatal and maternal death, preterm birth, and anaemia.

**Objectives:**

The objectives of this paper are to assess patterns and trends in the use of postpartum family planning at the country level, to determine whether postpartum family planning is associated with birth interval and parity, and to identify the health services most closely associated with postpartum family planning after adjusting for socio-economic characteristics.

**Design:**

Data were used from Demographic and Health Surveys that contain a reproductive calendar, carried out within the last 10 years, from Ethiopia, Malawi, and Nigeria. All women for whom the calendar was completed and who gave birth between 57 and 60 months prior to data collection were included in the analysis. For each of the births, we merged the reproductive calendar with the birth record into a survey for each country reflecting the previous 60 months. The definition of the postpartum period in this paper is based on a period of 3 months postpartum. We used this definition to assess early adoption of postpartum family planning. We assessed variations in postpartum family planning according to demographic and socio-economic variables, as well as its association with various contact opportunities with the health system [antenatal care (ANC), childbirth in facilities, immunisation, etc.]. We did simple descriptive analysis with tabular, graphic, and ‘equiplot’ displays and a logistic regression controlling for important background characteristics.

**Results:**

Overall, variation in postpartum use of modern contraception was not affected over the years by age or marital status. One contrast to this is in Ethiopia, where the data show a significant increase in uptake of postpartum contraception among adolescents from 2005 to 2011. There are systematic and pervasive equity issues in the use of modern postpartum family planning by education level, place of residence, and wealth quintile, especially in Ethiopia where the gaps are very large. Disaggregation of data also point to significant sub-national variations. After adjusting for socio-economic variables, the most consistent health sector services associated with modern postpartum contraception are institutional childbirth and child immunisation. ANC is less likely to be associated with the use of modern postpartum family planning.

**Conclusion:**

Postpartum use of modern family planning has remained very low over the years, including for childbearing adolescents. Our results indicate that improving postpartum family planning requires policies and strategies to address the inequalities caused by socio-economic factors and the integration of family planning with maternal and newborn health services, particularly with childbirth in facilities and child immunisation. Scaling up systematic screening, training of providers, and generation of demand are some possible ways forward.

**Paper context**

Postpartum contraception remains very low in sub-Saharan Africa. This paper investigates missed opportunities in the use of postpartum family planning in association with the health services, after adjusting for socio-economic characteristics. There are systematic and pervasive equity issues in the use of modern postpartum family planning. After adjusting for socio-economic variables, the most consistent health sector services associated with modern postpartum contraception are institutional childbirth and child immunisation. The results point to missed opportunities for better integration of maternal, newborn, and family planning services.

Achieving universal access to voluntary family planning and contraception services requires enabling policy and political environments, quality services, demand generation interventions, and integration of health systems to address missed opportunities. A key marker for high quality and integrated services is postpartum family planning (1). Postpartum family planning refers to the prevention of unintended and/or closely spaced pregnancies in the period after delivery. The first 12 months following childbirth is a period when a subsequent pregnancy holds the greatest risk for mother and baby (1), but also when there are numerous health service contacts for postnatal care for both mother and baby (for instance, child immunisation, nutrition, etc.).

The benefits and importance of postpartum family planning are well documented and include, at the individual level, reduction in the risk of miscarriage, low birth weight, neonatal death, maternal death, preterm birth, anaemia, and premature rupture of membranes (2, 3). The risks of early neonatal, neonatal, and infant deaths are high for subsequent births between the first 9 and 18 months after the end of a pregnancy (3, 4). Moreover, family planning could avert more than 30% of maternal deaths and 10% of child deaths if pregnancies were spaced more than 2 years apart (5).

It is thus critical that the health system does not miss any opportunities to offer women postpartum family planning information and services to ensure healthy outcomes for mothers and babies and to empower women to choose when to have subsequent children as well as how many. At the global level, thousands of under-five child deaths could be prevented by ensuring appropriate birth spacing (5). In recent reviews on the use of family planning in the postpartum period, antenatal care (ANC) visits and skilled birth attendance were found to be associated with postpartum family planning (6, 7). Similarly, wealth, education, and place of residence were associated with family planning in the postpartum period. These correlations may be associated with the modality of delivery of family planning services (through health facilities or household-based by community health workers) or with the populations in terms of who they are (wealthiest vs. poorest) rather than the covariates themselves. Moreover, one can expect that high-parity births would be more likely to be followed by contraceptive use. This paper assesses between- and within-country variations for three countries – Ethiopia, Malawi, and Nigeria – to determine whether postpartum family planning is associated with birth intervals and parity and to identify the health services most closely associated with postpartum family planning (adjusting for socio-economic characteristics).

## Data and methods

The data used in this study are from the most recent Demographic and Health Surveys (DHS) in Ethiopia (2005 and 2011), Malawi (2004 and 2010), and Nigeria (2008 and 2013). The fieldwork for the surveys took place between 2004 and 2013 and they contain a reproductive calendar. These countries were chosen because they have a low or moderate level of family planning use and have conducted two recent surveys with the complete reproductive health calendar, which is necessary for analysing postpartum family planning for a large sample of births. They also capture the diversity of sub-Saharan African countries in terms of geography, culture, religious groups, level of decentralisation, population size, contraceptive prevalence, languages, regions, and economic indicators. All women aged 15–49 years for whom the calendar was completed and who had given birth in the previous 60 months were included in the analysis. The complete reproductive calendar typically records, for each of the 60 months preceding the interview, all pregnancies, births, and terminations, as well as use of family planning (7). We merged the DHS women's data set (individual recode, which includes the reproductive calendar) with the DHS birth record for each of the births recorded in a survey for the last 60 months for each country.

The definition of the postpartum period in this paper is based on a period of 3 months postpartum. We used this definition to assess early adoption of postpartum family planning. In fact, the reproductive calendar is based upon the women's memory of the past 60 months. Initiation of family planning might be remembered relatively accurately if family planning was initiated immediately postpartum. Rather than needing to remember a particular month, a woman could remember that she started using family planning immediately following birth, an event easily recalled or likely to be documented. A longer postpartum period is likely to make the users of postpartum family planning similar to all users of family planning, whereas the focus in this study is on early adoption (7).

Modern family planning methods as defined in the DHS include female sterilisation, male sterilisation, pills, intrauterine devices, injectables, implants, male condoms, female condoms, diaphragms, foam/jelly, the lactational amenorrhoea method, and emergency contraception. Initiation of modern family planning (defined as involving the use of modern methods of contraception) during the first 3 months after delivery was disaggregated by demographic and socio-economic factors, parity, and birth intervals, as well as opportunistic use of the health system (ANC, institutional delivery, and child immunisation). The average prevalence in the latest DHS for ANC, skilled attendance at delivery, and child immunisation (third dose of diphtheria–tetanus–pertussis, DTP3) are: 43, 10, and 37% for Ethiopia; 95, 71, and 93% for Malawi; and 61, 38, and 38% for Nigeria, respectively.

Descriptive results are presented in tabular form, as maps, or via equiplots to best illustrate trends or disparities. Logistic regressions were also conducted for each survey analysed. This analysis explored the factors of service delivery use that were most correlated with postpartum family planning, adjusting for socio-economic factors. All of the analyses were performed with Stata 13.0 statistical software, taking into account the design characteristics of the surveys. Ethics procedures were the responsibility of the institutions that commissioned, funded, or administered the surveys.

## Results

As can be seen in Fig. 1, postpartum family planning has improved in Ethiopia and Malawi over the last two surveys, with a 160 and 55% increase in the national average from the 5 and 9.5% baseline, respectively. In contrast, in Nigeria, not only there was no increase from 2008 to 2013, but postpartum family planning prevalence has decreased from an already low level of 5.9–3.8%.

**Fig. 1:** Trends in average percent modern postpartum family planning use (% PPFP) at 3 months, all women 15–49 years old, with 95% confidence intervals in Ethiopia, Malawi, and Nigeria. Source: DHS.

### Disaggregation of modern postpartum family planning by age and marital status

Figure 2 presents the differentials by 5-year age groups in the three countries. With the exception of the 40–44 age group in Ethiopia, there were statistically significant differences across all age groups over time in both Ethiopia and Malawi. In Ethiopia, the highest increases in 2011 were observed among adolescents (15–19), youth (20–24), and women aged 45–49, while in Malawi, the highest increases were observed for women above 35 years old.

**Fig. 2:** Distribution of modern postpartum family planning at 3 months, all women 15–49 years old, by age, with 95% confidence intervals in Ethiopia, Malawi, and Nigeria. Source: DHS.

Among adolescents 15–19 years old, modern postpartum contraception has increased by 4.3 and 1.8 times in Ethiopia and Malawi, respectively. In Nigeria, there has been a systematic decrease in contraceptive use by all age groups, and there was no significant difference in the decrease across the age groups.

Regarding marital status, there was a skewed distribution towards marital status in the analysis samples, with 98.5, 96.9, and 97.3% of women in a union in Ethiopia, Malawi, and Nigeria, respectively. Births outside of unions are thus not common in these three countries. Using the latest DHS, we did not find any significant variation in postpartum family planning use by marital status in the three countries (data not presented).

### Distribution of modern postpartum family planning by socio-economic status

We explored variations in postpartum use of modern contraception by education level, place of residence (urban vs. rural), and wealth quintile. The data are presented in Table 1 and equiplots are constructed for the three countries (Fig. 2). Overall, there is a strong and statistically significant association between the use of modern postpartum family planning and education level, place of residence, and wealth quintile across all three countries. The inequalities have increased over the years in Ethiopia and Malawi between women with secondary-level education and above and women with primary-level education or lower. There was no statistically significant effect of education level in Nigeria, where the reduction appeared similar across all levels of education. The patterns of differentials by place of residence and education level were similar to one another, with increased inequalities over time between the urban and rural areas in Ethiopia and Malawi. In Nigeria, the absolute inequality in access and use of modern contraception in the postpartum period remained constant. Finally, regarding differentials by wealth quintile, the use of modern postpartum family planning was significantly higher among the richest quintiles compared to the poorest across all three countries. In Ethiopia, the inequality in the use of modern postpartum family planning increased from 2005 to 2011, from a 12.4% differential to a 28.9% differential between the lowest and highest quintiles. In Malawi and Nigeria, there did not appear to be significant differentials by wealth quintile across the last two surveys.

**Table 1:** Postpartum use of modern family planning at 3 months among women 15–49 years old disaggregated by education, place, and wealth quintile in Ethiopia, Malawi, and Nigeria

	Ethiopia				Malawi				Nigeria			
			
	2005	2005 SDV	2011	2011 SDV	2004	2004 SDV	2010	2010 SDV	2008	2008 SDV	2013	2013 SDV
Education												
No education	1.6	0.2	6.8	0.3	7.2	0.6	12.7	0.6	2.5	0.2	0.9	0.1
Primary	5.7	0.7	13.9	0.7	10.0	0.4	14.2	0.3	6.7	0.4	4.4	0.3
Secondary >	26.4	1.9	42.3	2.4	13.0	1.2	19.8	0.9	10.7	0.4	8.2	0.3
Place												
Urban	21.2	1.3	34.4	1.2	12.1	1.1	22.1	1.1	10.1	0.4	7.0	0.3
Rural	1.9	0.2	6.3	0.3	9.1	0.3	13.5	0.3	4.1	0.2	2.1	0.1
Wealth quintiles												
Lowest	0.8	0.2	2.7	0.3	6.8	0.6	11.7	0.5	2.8	0.2	0.6	0.1
Second	0.9	0.3	6.6	0.6	7.6	0.6	13.0	0.6	3.4	0.3	1.4	0.2
Middle	2.2	0.4	6.1	0.6	8.8	0.6	14.6	0.6	4.2	0.3	2.5	0.2
Fourth	2.6	0.5	9.5	0.7	10.7	0.8	16.8	0.7	7.0	0.4	6.4	0.4
Highest	13.2	0.9	31.6	1.1	14.9	1.0	18.9	0.9	14.3	0.7	10.5	0.5
All	5.0	0.2	11	0.3	9.5	0.3	14.7	0.3	5.9	0.1	3.8	0.2

SDV, standard deviation of the mean percent using modern family planning at 3 months postpartum in the latest Demographic and Health Surveys; primary, primary completed; secondary >, above primary school.

### Distribution of modern postpartum family planning by parity and birth interval

Table 2 presents the pattern of modern postpartum contraception use in the three countries by parity and birth interval. The use of modern postpartum contraception remains low in Nigeria regardless of levels of parity. There was no significant difference attributed to parity in Malawi. In Ethiopia, the use of modern postpartum family planning varied significantly by parity and was higher for low parity with a drop-off by half in the use of modern postpartum family planning for parity of four and above. For birth interval, apart from a slight increase in modern postpartum contraception use among women with short birth intervals in Malawi, there was no significant effect of birth interval.

**Table 2:** Prevalence of modern postpartum family planning at 3 months among women 15–49 years old by parity and birth interval (preceding birth)

	Ethiopia 2011 DHS		Malawi 2010 DHS		Nigeria 2013 DHS	
			
Countries Covariates	Mean postpartum family planning	SDV	Mean postpartum family planning	SDV	Mean postpartum family planning	SDV
Parity						
One	16.8	0.9	13.1	0.6	3.7	0.3
Two or three	11.8	0.6	14.7	0.5	4.7	0.2
Four or five	6.0	0.5	15.2	0.6	3.9	0.3
Six or more	6.1	0.5	15.8	0.7	2.7	0.2
Birth intervals (preceding birth)						
<24 months	8.1	0.7	18.7	0.9	3.2	0.3
≥24 months	8.3	0.4	14.5	0.3	4.1	0.2

DHS, Demographic and Health Survey; SDV, standard deviation of the mean percent using modern family planning at 3 months postpartum in the two latest DHS.

### Sub-national variations in postpartum family planning in Ethiopia (2011), Malawi (2010), and Nigeria (2013)

There were very large regional differences in the proportions of modern postpartum contraception use in the three countries (Figs. 3–5), although the low prevalence of modern postpartum family planning across the three countries mirrors a generally low level of modern contraception in sub-Saharan Africa (8). Our study revealed that in Ethiopia 55% of childbearing women adopted modern postpartum family planning in the Addis Ababa region, followed by the Dire Dawa region (a city administration similar to Addis Ababa), where 21% of childbearing women adopted modern postpartum family planning in the 3 months following childbirth.

**Fig. 3:** Equiplots of modern postpartum family planning at 3 months, all women 15–49 years old, by education, place, and wealth quintile in Ethiopia, Malawi, and Nigeria.

**Fig. 4:** Mapping of prevalence of modern postpartum family planning use at 3 months, all women 15–49 years old, by region, Ethiopia. Source: 2011 DHS.

**Fig. 5:** Mapping of prevalence of modern postpartum family planning use at 3 months, all women 15–49 years old, by district, Malawi. Source: 2010 DHS.

In Malawi, there were between and within variations across the three regions of the country. In the Central, Southern, and Northern regions, 17, 13, and 11% of childbearing women had used modern postpartum family planning, respectively. However, when we disaggregated the data by region, we saw a significant difference between the Dowa district and the Nkhotakota district in the Central region, with prevalence at 29 and 12%, respectively. The same could be observed between the Rumphi district and the Mzimba district in the Northern region, with prevalence at 17 and 8%, respectively. A similar pattern was observed in the Southern region, pointing to the importance of sub-national analysis.

In Nigeria (Fig. 6), the prevalence of modern postpartum family planning was extremely low, with some outlier regions (South South and South West) and states (Delta, Edo, Imo, Lagos, and Oyo) where the prevalence was significantly above the national average. It is interesting to note that these five states were not necessarily the top five states for overall modern contraception use in Nigeria. An inquiry with development partners in Nigeria revealed that the observed differences may be associated with the Nigeria Urban Reproductive Health Initiative programme, which targeted urban slum dwellers in five selected states including Oyo (Ibadan) and Edo from 2009 to 2014 (9). The project utilised a holistic approach for family planning programming in the cities to target communities with a high population of urban poor. Similarly, the Delta and Edo states have benefited from the ACCESS Family Planning Program, which focused on postpartum family planning from 2008 to 2014 using an innovative systematic screening strategy (10, 11). In Malawi, specific investments with a focus on postpartum family planning are fairly recent and differences across the districts may more reflect differences in the socio-economic or demographic factors of the women. In summary, in all three countries, the observed variations point more to variance in opportunities in access and supply or social and cultural behavioural norms.

**Fig. 6:** Mapping of prevalence of modern postpartum family planning use at 3 months, all women 15–49 years old, by state, Nigeria. Source: 2013 DHS.

### Effects of selected health services on adoption of postpartum family planning

Along the continuum of points of contact for postpartum family planning we also explored the association of ANC visits, institutional delivery, and child immunisation (DTP3 as proxy) with postpartum modern contraceptive use. We modelled the variables individually and in a multivariate analysis, adjusting for socio-economic factors. The results are presented in Table 3. After adjusting for covariates, there were statistically significant associations between institutional delivery and child immunisation and the use of modern postpartum family planning across all three countries.

**Table 3:** Multivariate analysis of effect of contacts with the health system on modern postpartum contraception use

Variables	Adjusted OR	Lower CI	Higher CI
Ethiopia (2011)			
Antenatal care (1–3 visits)	1.12	0.89	1.41
Antenatal care (4 visits)^a^	1.92	1.54	2.39
Institutional delivery (yes)^a^	2.04	1.63	2.56
Child immunisation^a^ (completion of DTP3)	1.85	1.56	2.20
Education (primary)^a^	1.63	1.36	1.96
Education (higher)^a^	1.34	1.03	1.75
Residence (urban)^a^	0.68	0.52	0.90
Wealth quintiles – second^a^	3.04	2.02	4.57
Wealth quintiles – third^a^	3.55	2.38	5.30
Wealth quintiles – fourth^a^	4.48	3.06	6.57
Wealth quintiles – fifth^a^	7.48	4.94	11.33
Malawi (2010)			
Antenatal care (1–3 visits)	0.96	0.75	1.23
Antenatal care (4 visits)	1.08	0.85	1.39
Institutional delivery (yes)^a^	1.31	1.16	1.47
Child immunisation^a^			
(completion of DTP3)	1.47	1.29	1.69
Education (primary)	1.05	0.92	1.20
Education (higher)	1.12	0.94	1.34
Residence (urban)^a^	0.66	0.57	0.77
Wealth quintiles – second^a^	1.16	1.01	1.34
Wealth quintiles – third	1.13	0.98	1.30
Wealth quintiles – fourth	1.14	0.98	1.33
Wealth quintiles – fifth^a^	1.20	1.01	1.43
Nigeria (2013)			
Antenatal care (1–3 visits)	0.85	0.63	1.15
Antenatal care (4 visits)	1.15	0.94	1.42
Institutional delivery (yes)^a^	1.24	1.04	1.48
Child immunisation^a^			
(completion of DTP3)	1.34	1.14	1.57
Education (primary)^a^	1.96	1.51	2.53
Education (higher)^a^	2.20	1.70	2.83
Residence (urban)	1.00	0.84	1.18
Wealth quintiles – second^a^	2.83	1.76	4.57
Wealth quintiles – third^a^	3.70	2.30	5.96
Wealth quintiles – fourth^a^	5.81	3.60	9.38
Wealth quintiles – fifth^a^	8.52	5.19	13.99

a Significant at the 5% level. OR, odds ratio; CI, confidence interval; DTP3, diphtheria–tetanus–pertussis. Adjusted OR calculated from a multivariate logistic regression adjusting for socio-economic variables. Reference categories are no antenatal care visit (antenatal care), no education (education), no health facility delivery (skilled attendance), rural area (residence), no completion of DTP3 (child immunisation), and lowest quintiles (wealth quintiles).

The odds of using modern postpartum family planning after delivering at a health facility were, on average, twice as high compared to women who did not deliver at a health facility in Ethiopia, 1.3 times higher in Malawi, and 1.2 times higher in Nigeria. In Ethiopia, the odds of using modern postpartum family planning when a woman completed DTP3 for her child were twice that of women who did not. The strength of the associations were more modest, but still statistically significant, in Malawi and Nigeria. In general, we did not find that ANC was consistently associated with the adoption of postpartum modern contraception after adjusting for socio-economic variables.

## Discussion

### Demographic and socio-economic factors

The rationale and goal of family planning is to empower women and childbearing adolescents to decide on contraceptive methods, adopt the method of choice, and use this method for 2 years or longer, depending on the reproductive intentions of the woman or couple. Although the level of use of modern postpartum family planning was low across the three countries, the positive change (particularly among adolescents) in Ethiopia and Malawi is encouraging. The level of contraception use in the immediate postpartum period was lower than the overall modern contraception use among sexually active and childbearing adolescents (2, 12). This constitutes an increase in births, which may lead to increased unwanted pregnancies, high fertility, and subsequent high risks for maternal mortality, fistula (leading to continuous urinary or faecal incontinence), and poverty. We did not observe any effect of marital status on the postpartum use of modern contraception. The socio-economic characteristics (education, place of residence, and wealth quintile) of the women were significantly associated with the use of modern contraception in the postpartum period. However, the overall results of our analysis imply that other determinants beyond socio-economic status may be at play.

### Effects of parity and birth spacing

Apart from Ethiopia, where we observed a variation by parity, it was surprising to find no significant association between parity or birth interval and modern contraception use in the postpartum period. The expectation among some that high parity births would be more likely followed by contraceptive use is not borne out in any of the three settings. It is possible that high parity is an indication that a woman is, in fact, not likely to use family planning as she has not controlled her fertility before and therefore will continue that pattern. It is also possible that couples who have unplanned pregnancies continue to have unplanned pregnancies throughout the reproductive health lifecycle or that, once married, women are under pressure to have many children. Alternatively, maybe parity and birth spacing are not among the best predictors or determinants of modern postpartum contraception use in family planning. Regardless, given the low postpartum contraceptive use, there is increased vulnerability to unplanned pregnancy for individuals and couples, and particularly adolescent girls, in the three countries after childbirth.

### Sub-national variations

We observed sub-national variations at the state, regional, and district levels across all three countries. These variations highlight the importance of disaggregated data for evidence-based policy making and programme design. The difference between rural and urban areas could partly account for some of these differences, but the results also point to other plausible explanatory factors, as several districts or states within a region have a very different prevalence of use of modern contraception in the postpartum period. These other factors may reflect differences in supply (performance of the health system) and demand (including culture and social norms within communities).

### Effect of contact points with the health system

We did not directly analyse the effect of variance in the supply of contraceptives in this paper, although the difference between urban and rural areas is a proxy for supply and access, as supply and access are generally better in urban areas and for the wealthiest women. The coverage of opportunistic contacts with the health system (ANC, skilled birth at delivery, child immunisation) varies across the three countries. When exploring the association of opportunistic contacts with the health system, we did not see a consistent relationship between ANC visits (irrespective of the number of the visits) and use of modern contraception in the postpartum period across countries. Given the association between ANC and institutional delivery (13–15), the result may reflect the absence of provision of family planning information and services during ANC. It may also indicate that a woman does not act on information unless it is given to her at a moment when she can act upon it effectively. In addition, proper ANC visits, when well conducted, could be associated with effective postnatal care. The lack of capacity and integration of maternal health services with family planning services results in missed opportunities. In this analysis, we did not assess to what extent community health workers as part of the health system may influence postpartum family planning use. This is partly because we are looking at events that occurred many months or years before such questions were asked. We also explored the effect of either a visit by a family planning health worker or a visit to a health facility by the woman in the previous 12 months and whether information and counsel on family planning were provided to the woman during a visit to a health facility; we did not find an association in another study in Burkina Faso, Ethiopia, and Nigeria (12). The fact that these variables were not found in our analysis to be key determinants for adoption of modern contraception use warrants more investigation via research or surveys that specifically address postpartum family planning.

Overall, the most consistent associations found were institutional delivery and child immunisation. This may reflect the fact that women who use such maternal and newborn health services are also more likely to use postpartum family planning services, or that by attending these services they have more opportunities to be convinced to use them and are provided with a method, or both. The use of DTP3 represents a ‘dose–response’ relationship in the postpartum period, as it corresponds to the third visit after post-delivery discharge and provides plenty of opportunity to inform, educate, and offer postpartum family planning. Giving birth in a facility and child immunisation appear to be critical factors and potentially missed opportunities for improving postpartum family planning coverage. As indicated in the World Health Organization's publication on programming strategies for postpartum family planning (16), an understanding of both the health system in terms of how it is structured, organised, staffed, and financed as well as current government policies is essential to assess existing gaps and opportunities for offering family planning to postpartum women. Using standardised instruments and tools to identify and address each client's needs for family planning services during delivery or child immunisation would be critical. The focus for integration of postpartum family planning services and other health services should be on services that are at the point of childbirth or in the period immediately following. If family planning services are not available at the time of contact with the health system, women should be referred to facilities where they could access the family planning services that could not be provided to them immediately.

### Limitations

The measurement of family planning based on the reproductive calendar likely suffers from large recall errors. In Nigeria, where the modern contraceptive prevalence was already very low, postpartum family planning was even lower, which further limits meaningful disaggregation and analysis.

## Conclusions

This paper highlights the importance of understanding the demographic and social determinants, as well as the sub-national differences in modern postpartum contraception use in Ethiopia, Malawi, and Nigeria. There were effects by age group, but no significant variation by marital status, parity (with the exception of Ethiopia), or birth interval. There are pervasive equity issues in the use of postpartum family planning by education level, place of residence, and wealth quintile, but postpartum family planning behaviours seem more connected to the nexus of service delivery. In this study, opportunistic contacts with the health system through childbirth in facilities and child immunisation were significantly associated with the adoption of postpartum family planning and thus potentially represent the greatest currently missed opportunities for integration.

## References

[1] Conde-Agudelo A, Rosas-Bermudez A, Castaño F, Norton MH (2012). Effects of birth spacing on maternal, perinatal, infant, and child health: a systematic review of causal mechanisms. Stud Fam Plann.

[2] Conde-Agudelo A, Belizán JM, Lammers C (2005). Maternal-perinatal morbidity and mortality associated with adolescent pregnancy in Latin America: cross-sectional study. Am J Obstet Gynecol.

[3] DaVanzo J, Hale L, Razzaque A, Rahmand M (2007). Effects of interpregnancy interval and outcome of the preceding pregnancy on pregnancy outcomes in Matlab, Bangladesh. BJOG.

[4] Bongaarts J (2014). How exceptional is the pattern of the fertility decline in sub-Saharan Africa?. UN Population Division, Expert Paper No. 2013/4.

[5] Cleland J, Bernstein S, Ezeh A, Faundes A, Glasier A, Innis J (2006). Family planning: the unfinished agenda. Lancet.

[6] Ross JA, Winfrey WB (2001). Contraceptive use, intention to use, and unmet need during the extended postpartum period. Int Fam Plan Perspect.

[7] Winfrey W, Kshitiz R (2014). Use of family planning in the postpartum period. DHS Comparative Report No. 36.

[8] Darroch JE, Singh S (2013). Trends in contraceptive need and use in developing countries in 2003, 2008, and 2012: an analysis of national surveys. Lancet.

[9] Nigeria Urban Reproductive Health Initiative (NURHI) http://www.nurhitoolkit.org/nurhi-overview#.VT0ZBSFViko.

[10] ACCESS-FP: addressing unmet need for postpartum family planning http://www.jhsph.edu/research/centers-and-institutes/center-for-adolescent-health/research/project_page.html?gf_id=4581&project_id=7428.

[11] Ricardo V, Foreit JR, Ottolenghi E (2006). Adding systematic screening to your program: manual. FRONTIERS Manual.

[12] Hounton S, Barros A, Amouzou A, Shiferaw S, Maiga A, Akinyemi A (2015). Patterns and trends of contraceptive use among sexually active adolescents in Burkina Faso, Ethiopia and Nigeria: a cross-sectional study. Glob Health Action.

[13] Moyer CA, Mustafa A (2013). Drivers and deterrents of facility delivery in sub-Saharan Africa: a systematic review. Reprod Health.

[14] Tsegay Y, Gebrehiwot T, Goicolea I, Edin K, Lemma H, Sebastian MS (2013). Determinants of antenatal and delivery care utilization in Tigray region, Ethiopia: a cross-sectional study. Int J Equity Health.

[15] Iyaniwura CA, Yussuf Q (2009). Utilization of antenatal care and delivery services in Sagamu, south western Nigeria. Afr J Reprod Health.

[16] World Health Organization (2013). Programming strategies for postpartum family planning.

# Sub article: Potential confounding in the association between short birth intervals and increased neonatal, infant, and child mortality

**DOI:** 10.3402/gha.v8.29724

**Published:** 2015-11-09

**Authors:** Jamie Perin, Neff Walker

**Affiliations:** 1 Institute for International Programs, Bloomberg School of Public Health, Johns Hopkins University, Baltimore, MD, USA; 2 Department of Pediatrics, Center for Child and Community Health Research, Johns Hopkins School of Medicine, Baltimore, MD, USA

**Keywords:** family planning, fertility, contraception rate, confounding, attributable fraction

## Abstract

**Background:**

Recent steep declines in child mortality have been attributed in part to increased use of contraceptives and the resulting change in fertility behaviour, including an increase in the time between births. Previous observational studies have documented strong associations between short birth spacing and an increase in the risk of neonatal, infant, and under-five mortality, compared to births with longer preceding birth intervals. In this analysis, we compare two methods to estimate the association between short birth intervals and mortality risk to better inform modelling efforts linking family planning and mortality in children.

**Objectives:**

Our goal was to estimate the mortality risk for neonates, infants, and young children by preceding birth space using household survey data, controlling for mother-level factors and to compare the results to those from previous analyses with survey data.

**Design:**

We assessed the potential for confounding when estimating the relative mortality risk by preceding birth interval and estimated mortality risk by birth interval in four categories: less than 18 months, 18–23 months, 24–35 months, and 36 months or longer. We estimated the relative risks among women who were 35 and older at the time of the survey with two methods: in a Cox proportional hazards regression adjusting for potential confounders and also by stratifying Cox regression by mother, to control for all factors that remain constant over a woman's childbearing years. We estimated the overall effects for birth spacing in a meta-analysis with random survey effects.

**Results:**

We identified several factors known for their associations with neonatal, infant, and child mortality that are also associated with preceding birth interval. When estimating the effect of birth spacing on mortality, we found that regression adjustment for these factors does not substantially change the risk ratio for short birth intervals compared to an unadjusted mortality ratio. For birth intervals less than 18 months, standard regression adjustment for confounding factors estimated a risk ratio for neonatal mortality of 2.28 (95% confidence interval: 2.18–2.37). This same effect estimated within mother is 1.57 (95% confidence interval: 1.52–1.63), a decline of almost one-third in the effect on neonatal mortality.

**Conclusions:**

Neonatal, infant, and child mortality are strongly and significantly related to preceding birth interval, where births within a short interval of time after the previous birth have increased mortality. Previous analyses have demonstrated this relationship on average across all births; however, women who have short spaces between births are different from women with long spaces. Among women 35 years and older where a comparison of birth spaces within mother is possible, we find a much reduced although still significant effect of short birth spaces on child mortality.

**Paper context**

Previous analyses have demonstrated increased neonatal, infant, and child mortality with short preceding birth spaces. This relationship has been estimated as an average or adjusted average increased risk across all births; however, women who have short spaces between births tend to be different from women with longer spaces. Among those instances where a comparison of birth spaces within mother was possible, we estimated the effect of short birth spaces on these mortalities and found a reduced, although still significant, effect.

There is an extensive literature linking family planning programmes, contraceptive use, fertility, and the health of infants and young children. Many studies have found a strong association between increasing use of contraceptives or reduced fertility and decreases in child mortality. One explanation of this relationship has been that as women have access to contraceptives, their children are born in different circumstances more favourable for survival (1).

Circumstances at birth conferring excess risk that also tend to decline with increasing contraceptive use have been broadly defined by three categories: age of mother (young and old age), birth parity (first births and higher parity births), and short intervals of time between births or pregnancies (less than 24 or 18 months between births). There has been considerable effort to quantify the risk associated with these factors. In general, analyses to estimate relative risk have proceeded by comparing birth outcomes among various groups with observational data. These different birth conditions have been repeatedly linked to increased poor birth outcomes (e.g. low birth weight, prematurity, small for gestational age) using information from trial results and analysis of facility-based data (2–4).

For most low- and middle-income countries, analyses have generally used national household survey data with full retrospective birth histories [mostly Demographic and Health Surveys (DHS), but also some Multiple Indicator Cluster Surveys (MICS)] to link birth conditions and poor maternal and child health. Surveys have reasonable accuracy when it comes to their recorded birth histories and the real timing of births (5). However, surveys do not generally measure clinical conditions well, and so these analyses primarily link birth risk categories to the risk of mortality, including neonatal, infant, and under-five mortality. For example, mortality in children born to women under 18 has been compared to mortality in children born to women aged 18–34 and shown to be generally higher; mortality in children born to women 35 or older has also been shown to be higher than for children born to mothers aged 18–34 at birth.

The association between these factors and increased child mortality is not necessarily causal or attributable. Associations could be confounded by other factors, such as differences between mothers who have births with these conditions or circumstances that cause both short interbirth intervals and child mortality, such as limited access to health services, including contraception. In addition, an association could also be explained by women being more likely to use contraceptives and reduce fertility when child mortality has dropped (replacement effect) (6).

The relationship between birth spacing and mortality is of interest for determining the attributable fraction of child mortality and predicting the impact of related interventions with the Lives Saved Tool (LiST) (7). Analyses of the association between mortality and birth conditions have often necessarily been conducted with observational data (3). These studies have attempted to address issues of selection and to control for possible confounding. In an analysis with retrospective birth histories from a selection of DHS, Rutstein and Winter (8) used Cox proportional hazards by including additional factors such as mother's education, wealth quintile, and urban or rural residence as predictors in their proportional hazard regressions of neonatal, infant, and child survival (9). DaVanzo and colleagues also used Cox proportional hazards for infant and child mortality and their relationship to interpregnancy intervals, including socio-economic status and other factors as predictors (10). In a recent systematic review, Conde-Agudelo et al. analysed this interpregnancy interval and 57 other similar observational studies examining the relationship between birth spacing and adverse outcomes for maternal, perinatal, and child health. Their inclusion criteria allowed for adjustment for socio-economic status and maternal age, excluding only studies examining the unadjusted relationship (i.e. without any additional predictors) between birth spacing and mortality or other adverse outcome (11). These analyses are not directly comparable, because their definition of short birth interval and adverse outcomes and their sample populations are not necessarily consistent. However, they share the primary statistical methodology of assessing survival with Cox proportional hazards, and they both make adjustments for potential confounders by including additional factors in these regressions. In general, these analyses have not restricted their sample of births or mothers for analysis.

While in theory this approach eliminates some confounding, such adjustment does not necessarily rule out all possible bias due to related factors. First, there may be confounders that have not been measured or have been poorly measured, for example, access to health services or women's empowerment. Second, even confounders that are known and measured may violate model assumptions and lead to biased or imprecise estimates of effect (12).

Recent work by Kozuki and Walker (13) used a different approach to control for selection issues in the analysis of birth spacing. They limited analysis to birth histories from women who were over 35 at the time of survey. Then, within those women who had three or more births, they looked for a short-spaced birth and a regular-spaced birth from the same birth history using conditional logistic regression. The purpose of this approach was to eliminate differences between mothers as each mother provided a short-spaced and regular-spaced birth. In this analysis, there was still an increased risk of mortality for the children born with a short space, however, this risk (OR=1.32) was considerably less than had been reported by other analyses. Is the observed increased mortality risk associated with short birth spacing due to differences between mothers? The analyses presented in this paper seek to extend the work of Kozuki and Walker in assessing the sensitivity of the link between short birth spacing and increased risk of mortality in children.

In this paper, we have two primary purposes. First, we investigate the background differences between women who have short birth intervals and those women who do not. Second, we directly compare the standard cross-sectional analysis of mortality risk to the within-mother analysis.

## Methods

### Data

We used complete birth histories from 145 standard, interim, or continuous DHS household surveys conducted since 1998, including DHS phases III through VI, from 66 unique countries. We included retrospective birth histories from all of these surveys in our analysis of short birth intervals and the women who have children with short birth intervals, while excluding multiple births.

We used these birth histories to assess the potential for confounding in the relationship between short birth intervals and child mortality. To describe women with short birth intervals, we grouped mothers according to their shortest birth interval in four categories (less than 18 months, 18–23 months, 24–35 months, or longer) and summarised the wealth quintile, education, age, and fertility of each group. We also described the observed mortality rates for the children of these women in each category of preceding birth interval.

In addition to assessing the potential for confounding, we also analysed these birth history data to assess mortality risk. For this primary analysis, we also excluded birth histories from women who were younger than 35 when their birth history was recorded, at the time of survey. We limited our analysis to women who were older at survey for several reasons. First, these women are nearing the end of their childbearing years, and so we can estimate their fertility, an additional potential confounder for the effect of birth spacing on mortality (14). Second, we expect women with complete or nearly complete birth histories to have more information about birth spacing, because they have had more time to have children and variety in their interbirth intervals. Although some women who are younger when surveyed have short-spaced births, there is less information about them, because their fertility cannot be estimated and because there are fewer siblings for comparison.

### Statistical analysis

We analysed the relationship between birth spacing and neonatal, infant, and under-five mortality using two approaches. First, we estimated the mortality risk ratio by interbirth interval using a standard regression adjustment for potential confounding factors. We used Cox proportional hazards for mortality (survival) outcomes based on retrospective birth histories, censored at the appropriate age and including mother's education in three categories, along with wealth quintile, partner's education, need for family planning satisfied, completed fertility, and area of residence (urban/rural) as potential confounders in the regression analysis, described in Table 1. Although the DHS include a great wealth of cross-sectional information for recent births, the same information for retrospective births is not available. Facility delivery and skilled birth attendance, for example, are available only for births in the previous 5 years. Other salient factors, like immunisation coverage or mother's HIV status, are not available for birth histories because they are measured at a single point in time. Ideally, we would like to adjust for these factors, if they were available.

**Table 1:** Factors used for adjustment when estimating the effect of birth spacing on neonatal, infant, and child mortality in retrospective birth histories from household survey data

Factor	Description
Wealth quintile	Five categories (poorest, poorer, middle, richer, richest) of a wealth index based on household assets, household construction materials, and water and sanitation facilities
Mother's education	Educational attainment of mother or caretaker, in six categories: no education, incomplete primary, complete primary, incomplete secondary, complete secondary, and higher than complete secondary
Area	Type of place of residence (urban or rural)
Partner's education	Partner's education level in five categories: no education, primary, secondary, higher, and unknown
Family planning need satisfied	Met need for family planning services (yes/no)
Fertility	The total number of children born to a woman during her lifetime
Mother's age	Age of the mother at the time of each birth: 35 and older

In addition to this first approach, we estimated the mortality risk ratio by interbirth interval in a within-mother comparison. We also used Cox proportional hazards for mortality outcomes; however, we stratified by mother to compare mortality only for women who had variety in their interbirth spacing. This second analysis was similar to conditional logistic regression, where mortality outcomes are compared for different outcomes within woman, except that conditional logistic regression estimates odds ratios, while Cox regression estimates mortality rate ratios (9, 15). We used Cox regression to estimate relative neonatal, infant, and under-five mortality.

Stratifying proportional hazards regression matches births within woman and so strictly controls for all factors that are constant for mothers over their childbearing years (16). There is still potential for confounding in factors that change over time, for example, mother's age or access to care. It is also possible that education, area of residence, or socio-economic indicators may change over time. We analysed births only for women whose full birth history was available up to age 35, and so births to women who were younger than 35 at the time of survey are excluded. This disproportionately excludes recent births and births of low parity.

We estimated the effect of birth spacing separately for each survey using these two methods. We then combined these results from multiple surveys in a meta-analysis with random survey effects, weighted by the estimated standard error of the birth spacing effect for each survey, for an overall effect estimate for each method. In addition, we assessed the potential for confounding in the within-mother analysis by examining the association between birth spacing and parity. We also described those births that did not contribute to the second within-mother analysis.

## Results

### Differences between mothers and births

Table 2 describes age, average fertility, socio-economic status, and education of mothers in all 145 surveys classified by their shortest birth interval. Birth interval appears to be correlated with factors that are also associated with child mortality.

**Table 2:** Characteristics of mothers classified by their smallest observed birth interval, as raw averages across 145 surveys in 66 countries since 1998, among women who were at least 35 years of age when surveyed

Smallest birth interval	Total number of women (thousands)	Mean (SD) age at survey	Mean (SD) fertility	% in bottom wealth quintile^a^	% with no education
Only first births	45	40.5 (0.6)	1 (na)	12.7 (6.8)	28.9 (26.0)
<18 months	205	41.7 (0.6)	6.5 (1.3)	22.7 (3.7)	41.6 (30.6)
18–23 months	151	41.1 (0.6)	5.6 (1.1)	21.1 (4.2)	38.8 (30.6)
24–35 months	134	41.0 (0.6)	4.4 (0.8)	17.5 (4.0)	33.8 (29.2)
≥36 months	113	40.6 (0.7)	2.8 (0.3)	12.3 (5.9)	28.5 (26.8)

a Wealth quintile data do not include 1999 survey in Nigeria; na, not applicable.

These analyses reveal the differences that exist between mothers who have short-spaced births compared to those who do not. In general, the women who had at least one birth that came 18 months or less after a preceding birth had lower education, were more often from the poorest wealth quintile, and had higher levels of fertility. These factors, and perhaps others that are unmeasured, have the potential to confound the association between shorter birth intervals and risk of mortality in the children of these women.

Table 3 describes births for each chosen category of preceding birth interval. There are over 5 million births recorded by these surveys. Gender and parity do not appear related to birth interval; however, mortality for short intervals is higher than regular or long intervals, as expected.

**Table 3:** Characteristics of births by preceding birth interval in four categories, across 145 DHS since 1998, among women who were at least 35 years of age when surveyed

	Total number of births (thousands)	% (SD) male	Average (SD) birth order	Average under-five mortality rate per 1,000 live births (SD)
First births	645	51.5 (1.4)	1 (na)	128.9 (68.8)
Birth interval				
<18 months	332	51.4 (1.7)	4.2 (0.7)	202.2 (83.6)
18–23 months	406	51.1 (1.6)	4.2 (0.6)	142.4 (71.3)
24–35 months	789	51.0 (1.1)	4.2 (0.6)	108.8 (58.1)
≥36 months	859	50.9 (1.2)	4.2 (0.7)	68.5 (36.6)

Multiple births are excluded; DHS, Demographic and Health Surveys; na, not applicable.

### Method comparison

We first compared the crude mortality ratios to the adjusted ratios for both the standard regression adjustment approach and the within-mother analysis using the stratified Cox proportional hazards regression. As can be seen in Fig. 1a, standard regression-adjusted estimates are similar to the crude neonatal mortality ratios when comparing births with spacing under 18 months to births with spacing of 24–35 months. However, in Fig. 1b, stratified Cox proportional hazards regression for neonatal mortality estimated a smaller effect of short intervals compared to the crude neonatal mortality ratios. Mortality risk ratios for a preceding birth interval of 18–23 months compared to 24–36 months are also shown in Fig. 1c and d. The results for these slightly larger birth intervals are less different than those for intervals less than 18 months, although there still appears to be a lower mortality ratio using the within-mother comparison.

**Fig. 1:** Comparison of crude neonatal mortality rates with those estimated by a standard regression adjustment and by stratifying within mothers. Neonatal mortality ratios are shown for comparing births with preceding interval less than 18 months to 24–35 months [(a) standard regression and (b) stratified regression] and for comparing births with a preceding interval of 18–23 months to those with a 24–35-month interval [(c) standard regression and (d) stratified regression].

In Fig. 2, we present the overall estimated risk ratios for neonatal mortality for children born with a preceding birth interval under 18 months and between 18 and 24 months as compared to a birth interval of between 24 and 35 months across 145 DHS. We do this separately for both the standard adjusted regression model and the within-mother Cox proportional hazards model. As would be expected based on the results shown in Fig. 1, the within-mother comparisons provided smaller estimates of the risk ratios for neonatal mortality than the adjusted regression analyses. For the shortest interbirth interval (less than 18 months), the additional mortality risk was reduced by almost one-third (2.27, 95% confidence interval: 2.18–2.37 vs. 1.57, 95% confidence interval: 1.52–1.58) using the within-mother technique. The estimated effects of birth interval for neonatal, infant, and child mortality are shown in Table 4 for both the standard adjustment and within-mother methods. Our interpretation is that the risk ratio estimated within mother is a more accurate measure of the direct relationship between birth intervals and mortality than the risk ratio estimated by the adjusted regression for women over 35 years, because births to the same women have more in common than can be specified by what was available in these cross-sectional survey data.

**Fig. 2:** Results of the meta-analysis for the effect of birth spacing on neonatal mortality across 145 DHS, using a standard adjustment for a Cox proportional hazard regression, and another Cox regression estimating the effect of birth spacing on neonatal mortality within mother.

**Table 4:** Average neonatal, infant, and under-five mortality risk ratios by preceding birth interval with 95% confidence limits, across 145 DHS since 1998, for women who were at least 35 at the time of survey

Birth interval	Standard regression adjustment^a^	Cox regression stratified by mother
Neonatal mortality		
<18 months	2.28 (2.18, 2.37)	1.57 (1.52, 1.63)
18–23 months	1.33 (1.27, 1.39)	1.24 (1.20, 0.27)
24–35 months	(Reference)	(Reference)
≥36 months	0.67 (0.64, 0.70)	0.68 (0.66, 0.71)
Infant mortality		
<18 months	2.31 (2.23, 2.39)	1.53 (1.49, 1.58)
18–23 months	1.36 (1.32, 1.41)	1.21 (1.19, 1.24)
24–35 months	(Reference)	(Reference)
≥36 months	0.62 (0.60, 0.64)	0.68 (0.66, 0.70)
Under-five mortality		
<18 months	1.81 (1.75, 1.88)	1.41 (1.37, 1.46)
18–23 months	1.25 (1.22, 1.28)	1.17 (1.15, 1.19)
24–35 months	(Reference)	(Reference)
≥36 months	0.75 (0.73, 0.77)	0.73 (0.71, 0.75)

a Adjusted for wealth quintile, mother's education, area (urban or rural), partner's education, family planning need satisfied, fertility, and mother's age. Birth spacing effects were estimated by Cox proportional hazard regression and separately by another Cox regression estimating the effect of birth spacing on neonatal mortality within mother. DHS, Demographic and Health Surveys.

### Investigating possible selection issues of the within-mother comparisons

Matching births within-mother controls completely for factors that are constant over a woman's childbearing years. Education and socio-economic status, for example, would not be expected to change substantially during this time. Other factors identified for their relationship with child mortality are known to vary significantly during this time period.

There are two possible selection issues that might make the within-mother comparison biased. First, if there is a difference in parity between the short-spaced and regular-spaced births, it could confound the effect of short birth spacing with the association of mortality and parity. To test this possibility, we computed the average parity for each birth in the within-mother analysis for the various interbirth intervals. Not surprisingly, average parity among all births is highly variable across the DHS in this analysis. However, the difference between average parity of short-spaced births and the average parity of regular-spaced births is very small. The average parity of births with preceding space of 24–35 months was 3.76 (range: 2.3–4.6), while the average parity for births with less than 18 months preceding space is 3.82 (range: 2.3–4.7). The average parity for births with 18–24 months preceding space is 3.76 (range: 2.3–4.5). Scatter plots of these average birth orders are shown in Fig. 3. These findings rule out parity differences as an explanation of the reduced risk ratio found with the within-mother comparison.

**Fig. 3:** Average parity by survey and for three categories of preceding birth space, for 145 household surveys in 66 countries.

A second possible limitation of the within-mother comparison is that requiring mothers to have both a short- and a regular-spaced birth to contribute to the analysis might result in a biased sample of short-spaced births. This would occur because matching births with short spaces to births with optimal space within mother may exclude some births from analysis, since short-spaced births with no optimally spaced sibling have no potential match and do not contribute to the estimated effect of short birth spacing on mortality. This scenario inevitably arises for some women, for example, women with only two births. Such a woman can only have one birth with an interbirth interval, because first births are not subject to a birth space.

In order to assess this possible source of bias, we compared the total number of short-spaced births among women who were at least 35 years old at survey to the number that were used in the within-mother comparison. Of 2.4 million births to women surveyed at 35+years, there were 0.74 million with a preceding space less than 24 months. Overall, 0.69 million (93%) of these births had siblings who were not subject to a short preceding birth interval, and so were matched within-mother and contribute to analysis.

While overall the percent of births lost due to matching within mother is low, some surveys were more likely to exclude births than others. The 2007 Ukraine Survey, for example, has the lowest percent of matched short-spaced births (45%), so that more than half of all short-spaced births to women surveyed at 35+ years from this survey were not contributing to analysis. Figure 4 shows the distribution of the percent of short-spaced births with eligible matches for all 145 surveys in our analysis.

**Fig. 4:** Percentage of short-spaced births that could be matched to another birth for the same mother, in the Cox regression analysis stratified by mother. This percentage is shown for each of 145 household surveys.

This analysis suggests that overall the within-mother technique seems to provide a strong representation of short-spaced births to women who were 35 or older at the time of survey. The vast majority of surveys found that over 90% of short-spaced births could be matched with a regular-spaced birth. The one limitation is that for countries with low fertility such as Ukraine, the percent of matches is reduced and here the risk ratios estimated by the within-mother comparison may not be representative of all short-spaced births to women surveyed at 35 or older.

## Discussion

We estimated the effect of preceding birth interval in four categories on the risk of neonatal, infant, and under-five mortality with recent household survey data from 66 countries. We found ample evidence of a potential for confounding factors when estimating the relationship between birth spacing and mortality in these survey data. We then estimated the relative neonatal, infant, and under-five mortality risks based on two different methods: first, to replicate how this relationship has been determined historically, by including available factors in a proportional hazards regression and, second, with mothers as a unit of stratification among women 35 years and older at survey. Our assumption was that using mothers as strata would be a more efficient method to control for confounders, and the resulting effect estimate would be closer to a direct effect, since this analysis controls for all factors that are constant over a woman's childbearing years. Both this and the former regression adjustment method clearly indicated a statistically significant effect of birth spacing on child mortality. However, the effect estimated by the within-mother analysis was approximately 30% smaller than that estimated with the adjusted regression for neonatal mortality, strongly indicating that there is confounding in the regression-adjusted estimate. Rutstein and Winter (8) used methods similar to this standard adjustment to estimate relative mortality rates by birth-to-conception intervals, with an overall risk ratio for under-five mortality of 3.24 (95% confidence interval: 3.12–3.36) for a birth-to-conception interval less than 6 months and 2.33 (95% confidence interval: 2.25–2.42) for an interval of 6–11 months (8). Although the intervals used in our analysis are not exactly comparable, as we used interbirth time among women aged 35 at survey, we estimate the under-five mortality risk ratio for an interbirth interval less than 18 months to be 1.41 (95% confidence interval: 1.37–1.46).

We found a similar effect for our restricted sample with a standard adjustment method (2.28 for an interbirth interval of 18 months) to that found in a similar unrestricted analysis by Rutstein and Winter (8) (2.33 for a birth-to-conception interval of 6–11 months). The within-mother analysis effectively controls for some factors, although there is still potential for confounding by factors that change for individual women over time. There is no evidence among these survey data, however, that parity is different for different categories of birth spacing, and so parity has little to no potential to confound the effect of birth spacing on mortality.

## Limitations

We restricted our sample to women who were older at the time of survey, and so some births to young women were excluded. In addition, we were not able to include all short-spaced births in the within-mother analysis, because even among women providing birth histories at 35 years or older, not all births had appropriate matches, that is, births by the same mother that were not short-spaced. This was especially true for surveys or countries with low fertility rates. However, we expect that countries with low fertility are also in general countries with lower neonatal and child mortality. Thus excluding births primarily from low-fertility areas may be biased away from the null to estimate an effect larger than the direct effect. In addition, the overwhelming majority of surveys contributed at least 90% of all short-spaced births reported in the birth histories from older women. Finally, due to the matching and restriction of birth history information to older women, we were unable to generalise our results to the entire sample of interest.

## Conclusions

An estimate of the causal relation between birth spacing and neonatal, infant, and under-five mortality is of interest for determining the attributable fraction of child mortality and predicting the impact of interventions with the LiST software. A strong and statistically significant effect has consistently been identified of short preceding spaces on increased child mortality. Using matching and restriction, our study indicates an approximately one-third reduction in the risk of neonatal mortality. We present an alternative comparison of preceding birth spaces within women 35 years and older to estimate a still significant but less strong effect.

## References

[1] Rutstein SO (2000). Factors associated with trends in infant and child mortality in developing countries during the 1990s. Bull World Health Organ.

[2] Winikoff B (1983). The effects of birth spacing on child and maternal health. Stud Fam Plann.

[3] Conde-Agudelo A, Rosas-Bermudez A, Kafury-Goeta A (2006). Birth spacing and risk of adverse perinatal outcomes: a meta-analysis. JAMA.

[4] Kozuki N, Lee ACC, Silveira MF, Victora CG, Adair L, Humphrey J (2013). The associations of birth intervals with small-for-gestational age, preterm and neonatal and infant mortality: a meta-analysis. BMC Public Health.

[5] Arnold F (1990). Assessment of the quality of birth history data in the demographic and health surveys. Report No. 1: an assessment of DHS-I data quality.

[6] Doepke M (2005). Child mortality and fertility decline: does the Barro-Becker model fit the facts?. J Popul Econ.

[7] Walker N, Tam Y, Friberg IK (2013). Overview of the lives saved tool (LiST). BMC Public Health.

[8] Rutstein S, Winter R (2014). The effects of fertility behavior on child survival and child nutritional status: evidence from the Demographic and Health Surveys, 2006 to 2012. DHS Analytical Studies No. 37.

[9] Cox DR (1972). Regression models and life-tables (with discussion). J R Stat Soc Ser B.

[10] DaVanzo J, Hale L, Razzaque A, Rahman M (2008). The effects of pregnancy spacing on infant and child mortality in Matlab, Bangladesh: how they vary by the type of pregnancy outcome that began the interval. Popul Stud.

[11] Conde-Agudelo A, Rosas-Bermudez A, Castaño F, Norton MH (2012). Effects of birth spacing on maternal, perinatal, infant, and child health: a systematic review of causal mechanisms. Stud Fam Plann.

[12] Freedman DA (2008). On regression adjustments to experimental data. Adv Appl Math.

[13] Kozuki N, Walker N (2013). Exploring the association between short/long preceding birth intervals and child mortality: using reference birth interval children of the same mother as comparison. BMC Public Health.

[14] Kozuki N, Sonneveldt E, Walker N (2014). Residual confounding explains the association between high parity and child mortality. BMC Public Health.

[15] Connolly MA, Liang KY (1988). Conditional logistic regression models for correlated binary data. Biometrika.

[16] Cox DR (1975). Partial likelihood. Biometrika.
